# Understanding extreme sea levels for broad-scale coastal impact and adaptation analysis

**DOI:** 10.1038/ncomms16075

**Published:** 2017-07-07

**Authors:** T. Wahl, I. D. Haigh, R. J. Nicholls, A. Arns, S. Dangendorf, J. Hinkel, A. B. A. Slangen

**Affiliations:** 1 Department of Civil, Environmental and Construction Engineering and Sustainable Coastal Systems Cluster, University of Central Florida, 12800 Pegasus Drive, Suite 211, Orlando, Florida 32816-2450, USA; 2 Ocean and Earth Science, National Oceanography Centre, University of Southampton, European Way, Southampton SO14 3ZH, UK; 3 Engineering and the Environment, University of Southampton, 22 University Road, Southampton SO17 1BJ, UK; 4 Research Institute for Water and Environment, University of Siegen, Paul-Bonatz Street 9-11, Siegen 57076, Germany; 5 Global Climate Forum, Neue Promenade 6, Berlin 10178, Germany; 6 Division of Resource Economics, Albrecht Daniel Thaer-Institute and Berlin Workshop in Institutional Analysis of Social-Ecological Systems, Humboldt-University, Unter den Linden 6, Berlin D-10099, Germany; 7 Department of Estuarine & Delta Systems, Royal Netherlands Institute for Sea Research and Utrecht University, PO Box 140, Yerseke 4400 AC, The Netherlands

## Abstract

One of the main consequences of mean sea level rise (SLR) on human settlements is an increase in flood risk due to an increase in the intensity and frequency of extreme sea levels (ESL). While substantial research efforts are directed towards quantifying projections and uncertainties of future global and regional SLR, corresponding uncertainties in contemporary ESL have not been assessed and projections are limited. Here we quantify, for the first time at global scale, the uncertainties in present-day ESL estimates, which have by default been ignored in broad-scale sea-level rise impact assessments to date. ESL uncertainties exceed those from global SLR projections and, assuming that we meet the Paris agreement goals, the projected SLR itself by the end of the century in many regions. Both uncertainties in SLR projections and ESL estimates need to be understood and combined to fully assess potential impacts and adaptation needs.

## Abstract (web summary)

Uncertainties in contemporary extreme sea levels (ESL) from mean sea level rise (SLR) projections have been overlooked in broad-scale risk and adaptation studies. Here, the authors quantify the uncertainties in present-day global ESL estimates and find that they exceed those from global SLR projections.

Up to 310 million people residing in low elevation coastal zones are already directly or indirectly vulnerable to ESL^1^ and coastal storms are causing damages in the order of tens of billion US$ per year^2^. These numbers could increase dramatically with SLR^3^ and other changes^4^, leading to annual damages of up to almost 10% of the global gross domestic product in 2100 if no adaptation measures are taken^5^. Hence, there is a need for assessments of potential changes in coastal flood risk and adaptation strategies to manage these risks^1^,^5^,^6^,^7^. Understanding and considering the uncertainties related to future climate and socio-economic change is thereby essential to avoid maladaptation^8^,^9^. Despite this, uncertainties in present-day and future ESL estimates have not been assessed at continental (with the exception of ref. 10 for the US coast) and global scales and hence have been ignored in all previous broad-scale coastal risk and adaptation studies. Here we quantify present-day ESL uncertainties in terms of their two key sources: models used to generate multi-decadal time series of storm surges that cover the entire global coastline, and statistical methods based on extreme value theory used to parameterize extreme events. Furthermore, we compare those two key uncertainties in contemporary ESL estimates to uncertainties in global and local SLR projections and to projected future ESL changes in Europe by the end of the century. The dynamic contribution of waves (wave setup or runup) is not considered here due to a lack of global data, but also represents an important component of extreme total water levels that has been ignored in broad-scale coastal risk assessments thus far (with the exception of ref. 7 for the European coast). We find that uncertainties in contemporary ESL estimates are at least as important as uncertainties in SLR projections, with the latter becoming more dominant when focusing on time periods farther in the future. More research is needed to assess present-day and future ESL and associated uncertainties at global scale and results need to be integrated with SLR projections and their respective uncertainties into impact and adaptation studies.

## Results

### Uncertainties from extreme value analysis

**Figure 1:**
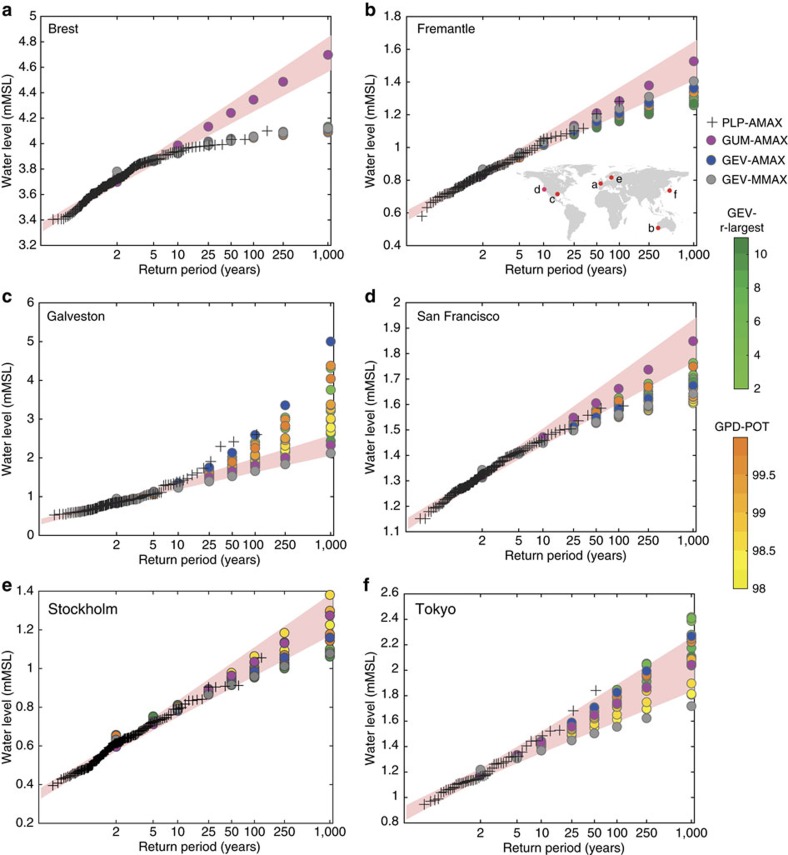
Spread from using different extreme value analysis methods. (**a**–**f**) Return water levels are shown for selected tide gauge sites (see panel **b** for locations). Plotting positions (PLPs) were obtained with the Weibull formula from the observed annual maxima time series (AMAX) and are therefore directly comparable to the Gumbel (GUM) and Generalized Extreme Value (GEV) fits to AMAX but not to results obtained with the Generalized Pareto Distribution (GPD) for varying thresholds and the GEV fits to r-largest time series. Shaded bands are 95% confidence bounds of the GUM-AMAX method.

In broad-scale impact assessments, ESL are typically represented by a set of parametric distributions that are also commonly used by engineers to design (coastal) infrastructure or to define flood zones (for example, for insurance purposes)^11^,^12^. Applying extreme value analysis (EVA) methods to observed or modelled extreme water levels allows the quantification of return periods (or return levels) that are longer than the observed records; for example, a 30-year long tide gauge record can be used to quantify the water level that is exceeded, on average, once every 100 years (that is, it has a 1% chance to be exceeded in any given year). A wide range of such statistical methods exists, and while particular approaches are preferred when certain criteria are fulfilled (for example, sufficient data length) or to meet a specific project goal (for example, localized analysis as part of infrastructure design), there is no universally accepted standard or best approach for broad-scale impact and adaptation analysis^13^. The simplest technique is to fit a Gumbel distribution with two parameters (location and scale) to a time series of annual maxima still water levels comprised of tidal and storm surge components (GUM-AMAX). This was used, for example, to obtain return water levels from a global storm surge model hindcast^14^ and to create global maps of sea level rise allowances^3^,^15^,^16^. Other methods make better use of the available data, for example, by selecting more than one extreme event per year (r-largest method) or by using a peaks-over-threshold approach to identify extremes^11^,^17^. The more advanced assessments also consider flexible distributions, typically a Generalized Extreme Value (GEV) or Generalized Pareto distribution (GPD). Both have a third parameter (shape) and in comparison to the Gumbel distribution which appears as a straight line in the double-logarithmic space they can be curved to better represent the most extreme events (Fig. 1).

The spread in the results attained by applying different EVA methods (Methods section) can be significant as shown for six examples in Fig. 1. This is particularly true for those ESL events of most interest: with low-probability but high impact potential. Extrapolating to the 1,000 year event based on the available data length is beyond the extrapolation period that is typically recommended (ie, up to approximately four times the length of the observational record)^18^. However, to be useful for broad-scale impact and adaptation analyses it is crucial to include such low-probability/high impact events. They largely determine the flood risk in highly developed and well-protected areas, such as Europe. For example, a 1-in-10,000 year design standard has been adopted for the most densely populated areas of the Netherlands and is also used in other countries to protect critical coastal infrastructure (for example, nuclear power plants).

**Figure 2:**
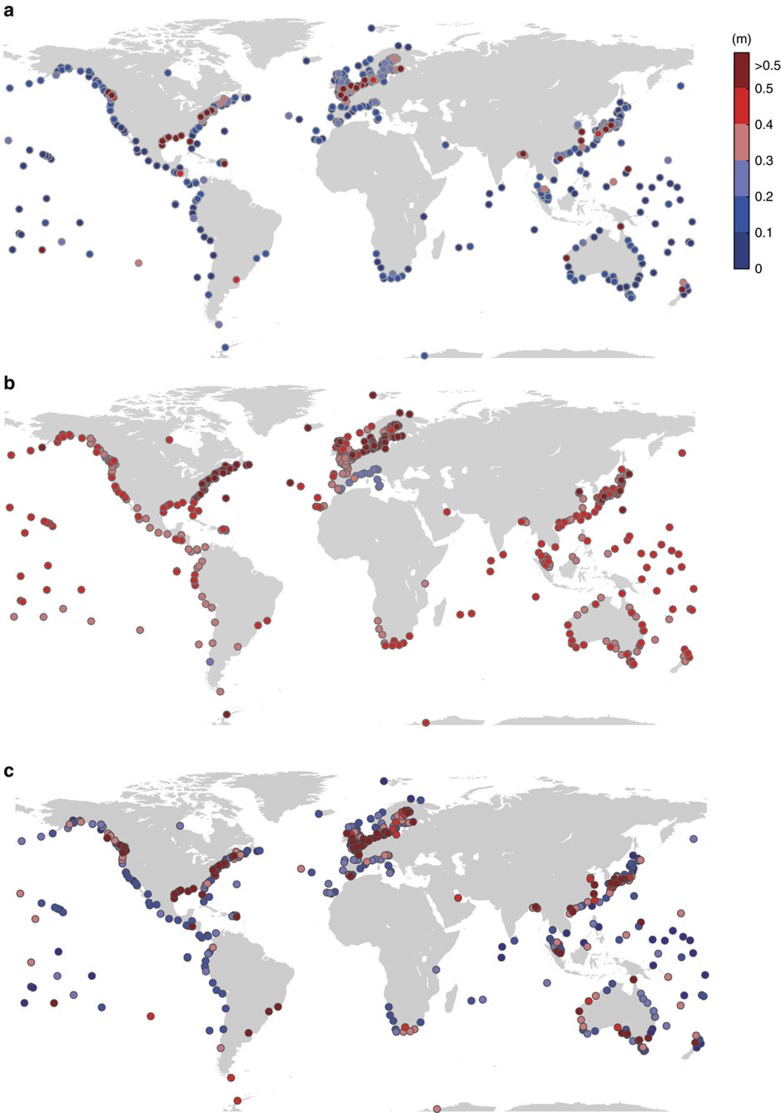
Uncertainties in present-day extreme sea level estimates and in regional sea-level rise projections. (**a**) 5 to 95% inter-model uncertainty ranges in 100-year return water levels. (**b**) 5–95% uncertainty ranges in regional sea- level rise projections under the Representative Concentration Pathway 4.5 scenario (2081–2100 mean minus 1986–2005 mean) at grid points closest to the tide gauge locations. (**c**) Combined extreme sea level uncertainties (100-year events) from summing up the 5–95% inter-model uncertainties from the extreme value analysis and (absolute) offsets found in the Global Tide and Surge Reanalysis (GTSR) model data set.

Here we use a recently assembled quasi-global tide gauge data set with high temporal resolution called GESLA-2 (ref. 19), and assess the inter-model uncertainties from a representative sample of 20 different EVA methods (this facilitates direct comparison to future SLR uncertainties; see below). From these methods we obtain local return water level estimates at 510 individual tide gauge stations, each of them providing at least 20 years of nearly complete data (Supplementary Fig. 1a). The 5–95% uncertainty ranges (Methods section) across the different extreme value models for the 100-year events (that is, events with a 1% exceedance probability in any given year) extend from less than 10 cm at many sites along the US west coast, South America, Mediterranean, and parts of Australia to more than a meter along the US east coast, East Asia and northern Europe (Fig. 2a). The average 5–95% range across all sites is 22 cm for the 100-year events and 60 cm for the 1,000-year events (that is, events with a 0.1% exceedance probability in any given year). In general, the spread is larger in regions where the range between moderate and the most extreme storm surges is large, for example, due to the rare occurrence of land falling hurricanes (US southeast and East Asia) or strong extra-tropical storms that can cause massive storm surges in the northeast Atlantic and southeastern North Sea. In regions where the variability of storm surge water levels is relatively small, the spread across EVA results is also small.

**Figure 3:**
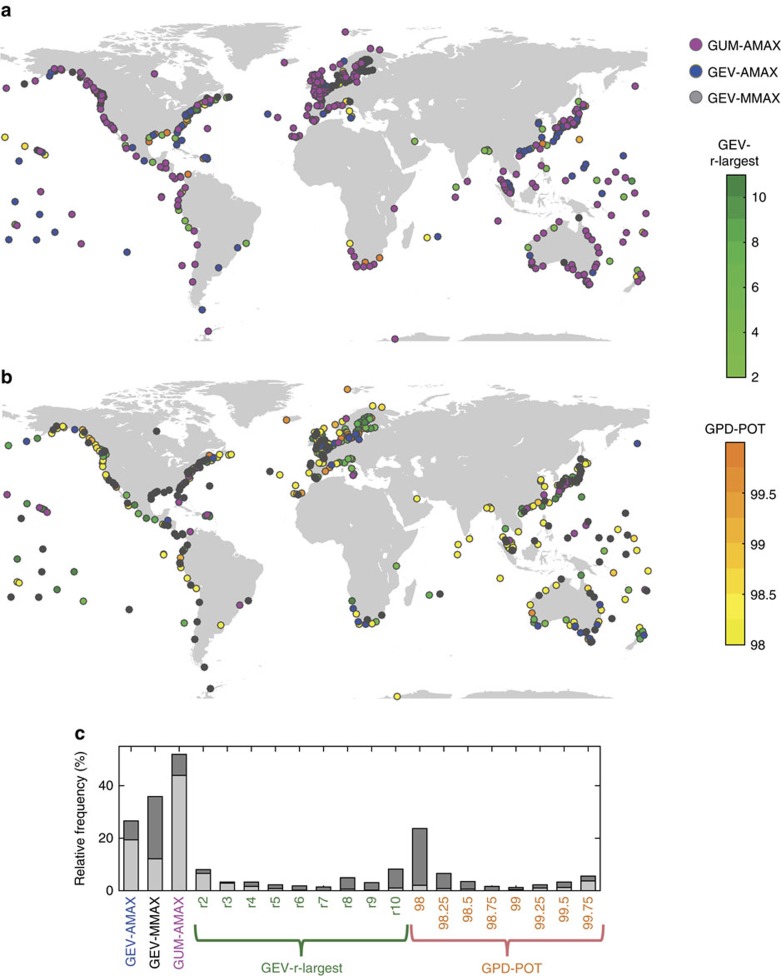
Extreme value analysis methods leading to conservative and optimistic results. (**a**) Methods leading to the highest and (**b**) methods leading to the lowest 100-year return water levels at individual sites. (**c**) Relative frequency of different methods leading to the highest (light grey) and lowest (dark grey) 100-year return water levels.

When the 95% confidence bounds of the GUM-AMAX approach are compared to the central estimates from the other EVA methods, we find a large percentage of sites where the results from all other methods lie within the GUM-AMAX confidence bounds (hereafter referred to as hit rates) for the 10-year events (82%), but much smaller ones for the 100- and 1,000-year events (35% and 4%, respectively; Supplementary Fig. 2). Among the methods considered here, the popular GUM-AMAX approach for global assessments^3^,^14^,^15^,^16^ tends to be most conservative for contemporary ESL estimates. It leads to the highest 100-year return water levels at more than 45% of the sites, followed by the GEV-AMAX approach at 19% of the sites (Fig. 3a,c). Fitting a GEV to monthly maxima (GEV-MMAX) results in the lowest 100-year levels at 25% of the sites, followed by the GPD98 method (that is, fitting a GPD to the 98th percentile threshold exceedances) at 21% of the sites (Fig. 3b,c). The GPD99 approach almost never leads to the highest or smallest return water levels and is identified here as the preferred approach to assess ESL at global scale.

Uncertainties also arise from the limited lengths of the records available for the analysis^20^. This is tested for the GPD99 method at all sites that have at least 70 complete years of data (43 sites in total; Methods section and Supplementary Fig. 3). The mean absolute errors across all sites in the 100-year return water levels are 15, 8 and 3 cm for record lengths of 20-, 35- and 50-years when compared to the results from analysing the full 70-year data sets (see also Table 1).

**Figure 4:**
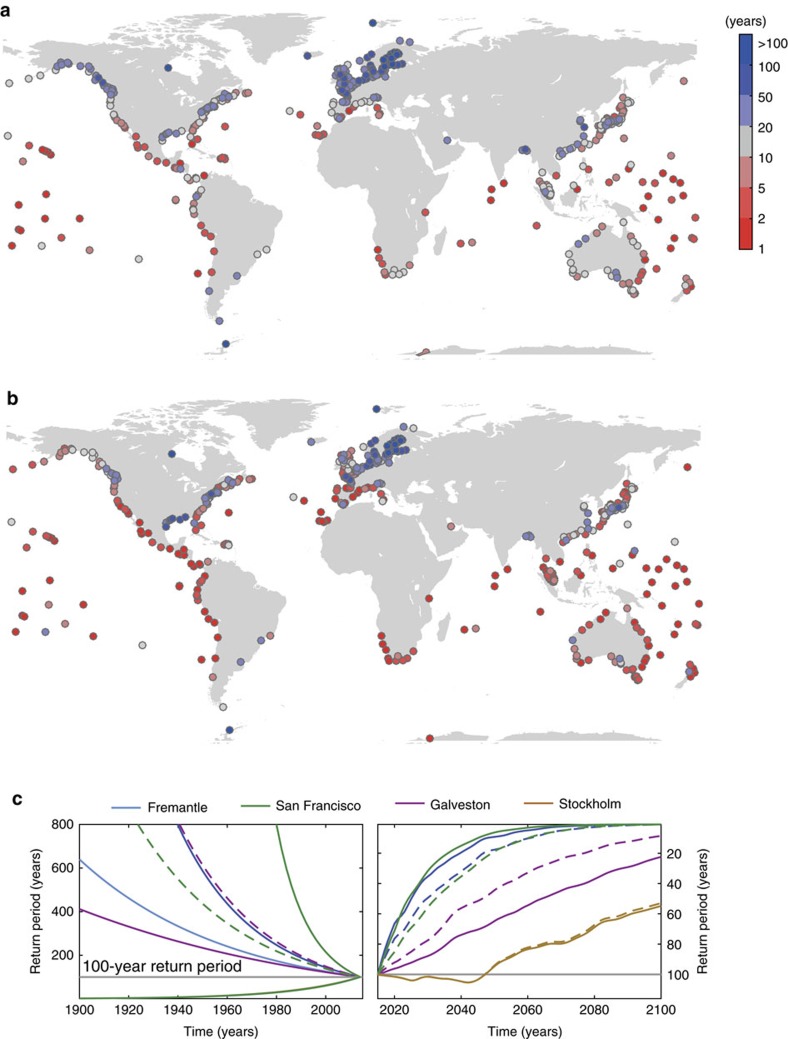
Changes in return periods due to sea-level rise when using different extreme value analysis models. (**a**,**b**) Return period in 2050 (assuming regional relative sea-level rise (SLR) under Representative Concentration Pathway (RCP) 4.5 scenario) of present-day 100-year water level when using (**a**) Gumbel distribution with annual maxima (GUM-AMAX) and (**b**) Generalized Pareto Distribution with 99th percentile threshold exceedances (GPD99). (**c**) Changes in return period of present-day 100-year water levels through time (1900 to 2100; RCP4.5 scenario) at four selected sites: Fremantle (blue; past SLR trend 1.73 mm per year), Galveston (red; past SLR trend 6.31 mm per year), San Francisco (green; past SLR trend 1.87 mm per year), and Stockholm (brown; past SLR trend −3.78 mm per year). Results are shown for GUM-AMAX (dashed lines) and GPD99 (solid lines); horizontal grey line represents the 100-year return period. Locations of the four sites can be seen in the inset in Fig. 1b.

Assuming that ESL follow a distribution with a shape parameter (for example GPD) also has significant implications for future changes in return periods (or exceedance probabilities) when distributions are vertically displaced with sea-level change, assuming that no or only negligible long-term trends in storminess exist^12^. Here we analyse changes in the return period by 2050 due to changing sea level^21^,^22^ under the Representative Concentration Pathway (RCP) 4.5 of what is currently being assumed a 100-year event; we compare results from GPD99, identified here as a good global approach to parameterize ESL, and the GUM-AMAX method used in previous global assessments^3^,^14^,^15^,^16^ (Fig. 4a,b). At most sites (85%), the shape parameter from the GPD99 method is negative. This indicates that the distribution is bounded (that is, it flattens in the tail) and changes in return periods occur faster with SLR as compared to the GUM-AMAX approach. The reverse occurs when the shape parameter is positive (that is, the distribution is unbounded), as sometimes found in regions prone to tropical cyclones. The GPD99 method leads to a sharper reduction in return periods (or stronger increase in frequencies of extreme events) on the west coast of the Americas and parts of Australia, the Mediterranean, northern Europe, and East Asia. At many sites what is currently a 100-year event will statistically occur at least once per year in 2050 according to both methods (37% of the sites with GUM-AMAX and 60% with GPD99).

For selected sites the changes through time in the return periods from 1900 through to 2100 are shown in Fig. 4c. For the past we assume that local sea level change was linear and use the respective observed relative trends for the 20th century^23^. For the future we use annual time series from 2015 to 2100 of regional relative SLR projections (ensemble mean under the RCP4.5 scenario) representative of the tide gauge locations^21^,^22^ (for both past and future we assume no change in storminess). San Francisco and Fremantle have negative shape parameters and hence the return periods associated with the present-day 100-year water levels have decreased faster with the GPD99 method in the past and will continue to do so with future SLR. Galveston has an unbounded distribution and changes occur faster when using the GUM-AMAX approach for both past and future. Results for Stockholm highlight the importance of vertical land movement. Due to strong land uplift in the area as a result of Glacial Isostatic Adjustment^24^, water levels that are currently assumed to be exceeded on average once every 100-years, previously would have been expected to occur every year at the start of the 20th century, according to both EVA methods. Over the next few decades, however, accelerated regional SLR under the RCP4.5 scenario will outpace the ongoing land movement and at the end of the century the present-day 100-year level will be exceeded (on average) approximately every 50 years.

### Uncertainties from storm surge models

So far we discussed ESL point estimates at tide gauge locations, but broad-scale impact and adaptation assessments require such information continuously for all coastal areas. With tide gauges providing only point data and the application of remote sensing techniques for spatially continuous observation of ESL events at its infancy^25^ we have to rely on models to simulate water level data for all coastal grid points, which can then be parameterized with one of the EVA methods discussed above. For a long time, the only global data set with such information was the DINAS-COAST (D-C) data set, created more than a decade ago with a simple empirical model^26^,^27^. Recently, the first global dynamic surge model was developed and used to produce a 35-year global tide and surge reanalysis (GTSR)^14^. In both cases the GUM-AMAX approach was used to derive return water levels and we can compare the results obtained from the simulations to the GUM-AMAX estimates (for consistency) derived here from observations.

**Figure 5:**
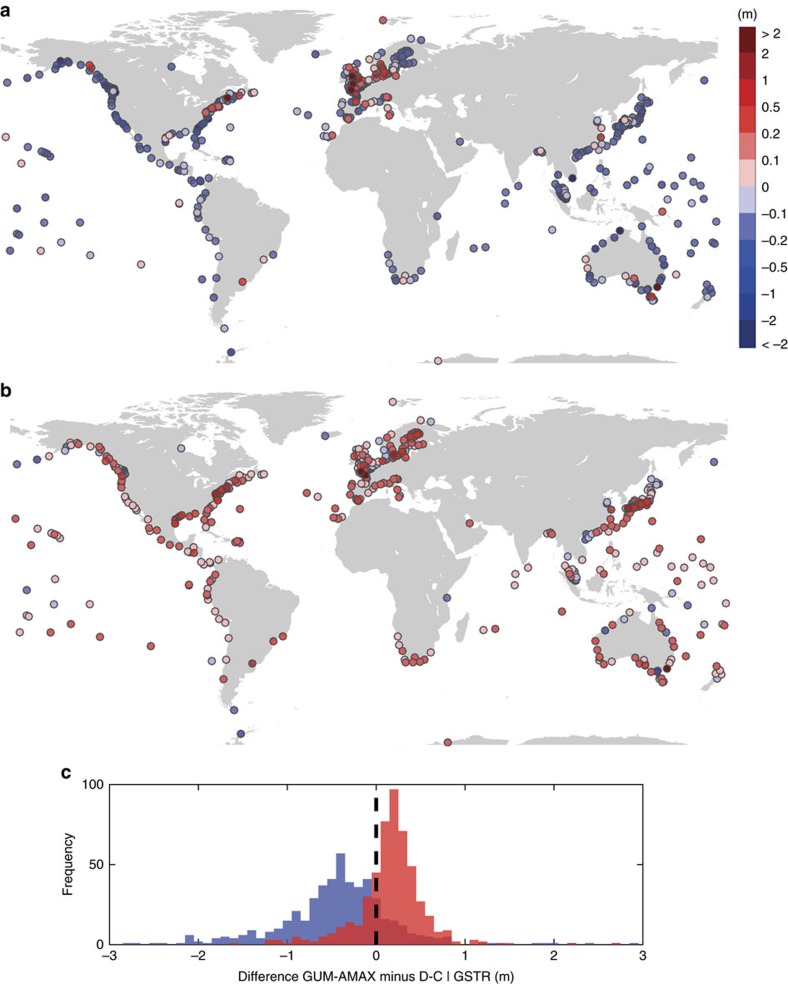
Offsets between return water levels derived from observations and models. (**a**,**b**) Differences in 100-year return water levels obtained from tide gauge observations with the Gumbel distribution and annual maxima (GUM-AMAX) method and those from the (**a**) DINAS-COAST (D-C) and (**b**) Global Tide and Surge Reanalysis (GTSR) data sets (negative values indicate that models overestimate extreme sea levels). (**c**) Histograms of differences between 100-year return water levels from observations and D-C (blue) and GTSR (red).

We find that D-C overestimates 100-year return water levels at the majority of sites, except for parts of northern Europe and the northeastern United States where it underestimates them (Fig. 5a). The average absolute error is 64 cm for the 100-year water levels (s.d. 88 cm) and 69 cm for the 1,000-year water levels (s.d. 109 cm). GTSR generally underestimates ESL (Fig. 5b), but has much smaller errors compared to the observations (Fig. 5c). Improvements are most notable in the western Pacific, Indian Ocean, northern Europe, and along the US west coast. The average absolute error decreases to 33 cm (s.d. 58 cm) for the 100-year return water levels and 41 cm (s.d. 78 cm) for the 1,000-year return water levels. Both models capture the spatial variability of extremes reasonably well but with offsets. This leads to hit rates of only 16% for D-C and 22% for GTSR where the 100-year return water levels obtained from the models lie within the 95% confidence levels of the GUM-AMAX estimates from the observations (Supplementary Fig. 4). The errors in both models reach several meters in regions with complex tidal regimes (for example, English Channel or Bristol Channel).

One way to correct for these offsets would be to use the observations and carefully interpolate the bias between tide gauge sites. This procedure has already been used to correct the output from regional storm surge models for EVA^28^,^29^,^30^. It can be done with shorter records to further improve the global coverage; for example, close to 800 sites in the GESLA-2 database^19^ provide 5 years (or more) of sea level data (Supplementary Fig. 1b). The results of impact and adaptation assessments will also depend on how coastal defenses are represented. Due to lack of data they are often reported as a standard of protection (that is, a return period)^1^,^5^, so as water levels change defense heights are automatically adjusted, and the results will change less than expected. In general, the effects on calculated flood damages and adaptation costs due to these offsets will be larger in studies where defenses are excluded or assumed to have fixed heights.

### SLR versus ESL uncertainties

**Figure 6:**
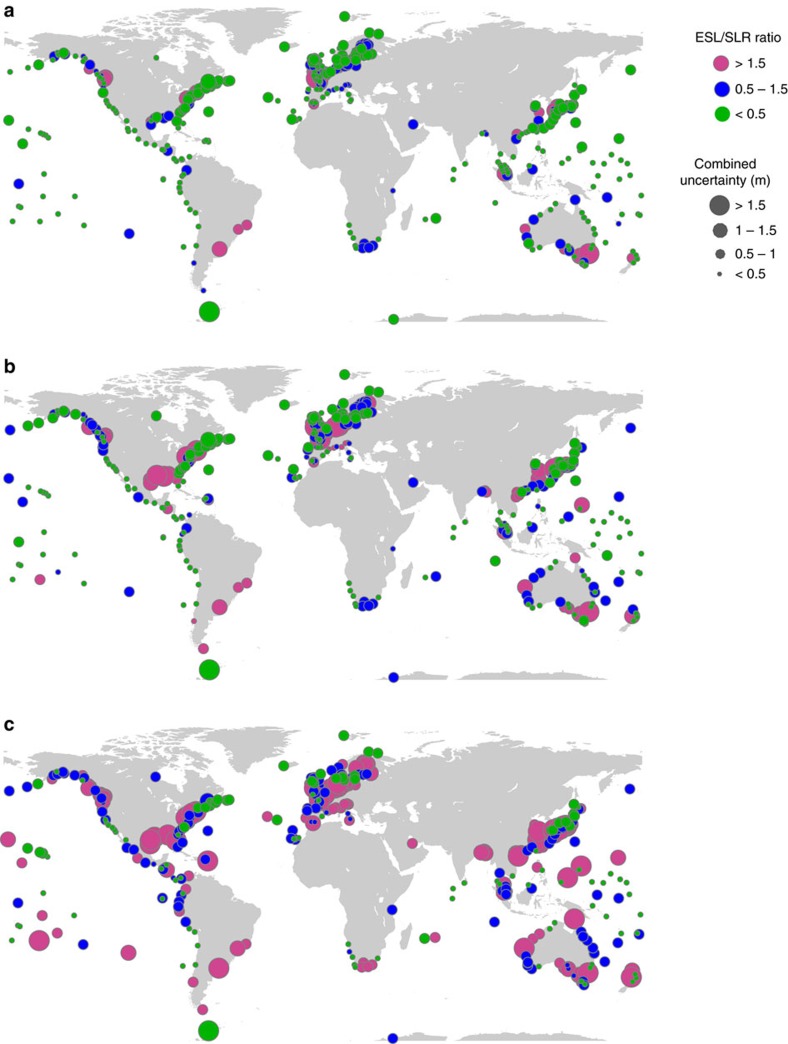
Combined regional sea-level rise and extreme sea level uncertainties. Combined uncertainties (5–95% range; see circle sizes for magnitude) in future sea-level rise (SLR) projections (Representative Concentration Pathway 4.5 scenario and 2081–2100 mean minus 1986–2005 mean) and present-day extreme sea level estimates and their relative importance (see colour coding) for the (**a**) 10-year, (**b**) 100-year and (**c**) 1,000-year return periods.

**Figure 7:**
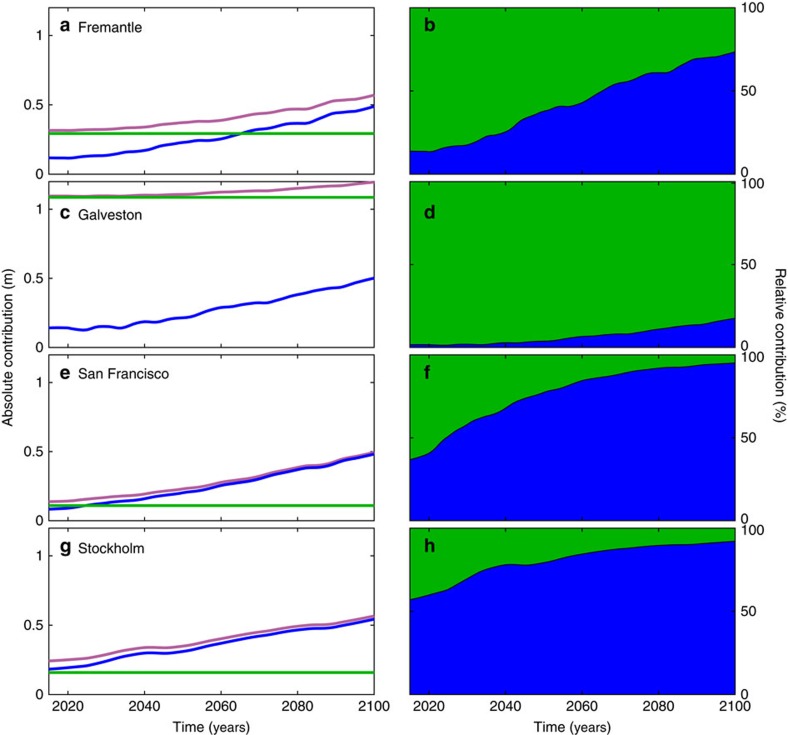
Temporal changes in extreme sea level (here for the 100-year events) and sea-level rise uncertainty contribution. (**a**,**c**,**e**,**g**) Changes through time (2015–2100) in the combined (red) and individual sea-level rise (SLR) (blue) (Representative Concentration Pathway 4.5 scenario) and extreme sea level (ESL) (green) uncertainties for the 100-year events. (**b**,**d**,**f**,**h**) Changes in the relative contribution of ESL (green; 100-year events) and SLR (blue) uncertainties through time. Results are shown for the same four sites as in Fig. 4c, with their locations depicted in Fig. 1b.

When we compare the combined uncertainties (Methods section) in present-day ESL estimates due to different EVA methods and storm surge model offsets to future regional SLR uncertainties, we find that the former dominate over the latter in many (high risk) regions such as Europe, East Asia, and the eastern and northwestern United States (Fig. 2b,c). Fig. 6 shows the magnitude of the combined (5–95% range; Methods section) present-day ESL and future SLR^21^,^22^ (RCP4.5 scenario and 2081–2100 mean minus 1986–2005 mean) uncertainties for different return periods. This highlights how the relative importance of ESL uncertainties increases for more extreme events. At the same time, their relative importance decreases when focusing on time periods farther into the future (Fig. 7). The ESL uncertainties derived here are for present-day climate; they may change in the future as more data becomes available, but the results in Fig. 1 for some of the longest records show that large uncertainties may persist. Hence we assume constant ESL uncertainties in the future, whereas uncertainties in SLR projections increase throughout the century and become more important. Note that ESL uncertainties from insufficient record lengths are not included in Figs 2c, 6, and , because they are only available at 43 sites. Across those 43 sites the average error when using 35 years of data (ie, the length of the GTSR model hindcast) instead of 70 years are 3, 7 and 13 cm for the 10-, 100- and 1,000-year return water levels, respectively (Table 1).

In addition to uncertainties at individual sites, discussed thus far, Table 1 summarizes uncertainties in global average SLR projections (in total and from individual components) from the 5th Assessment Report (AR5) of the Intergovernmental Panel on Climate Change (IPCC)^3^. Many previous broad-scale impact studies used such global average projections^31^. To put our ESL results into context, we calculate the average ESL uncertainties across all tide gauge sites for the different types of uncertainties, separately and combined (Methods), and for the 10-, 100- and 1,000-year return periods. Similarly to what was found for many regions, the combined global average uncertainties in contemporary ESL estimates are larger than the uncertainties in global SLR projections for events with longer return periods (Table 1). For most climate scenarios, present-day ESL uncertainties are also in the order of or larger than the expected global SLR itself, which would be 0.5 m (or less) by 2100 if we achieve the Paris agreement goals^32^,^33^. Other local and global SLR projections (for example, Table 13.6 in ref. 3 and ref. 34) exist and may have smaller or larger uncertainties than the ones we concentrate on. For example, the 5–95% ranges in local and global SLR projections from ref. 34 are larger than the ones considered here due to wider tails in the distributions of the ice-sheet components^35^.

Our analysis ignores potential future changes in the storm surge climate, because these have to date only been assessed at local and regional scales, sometimes with opposing outcomes. Hence, it was concluded in the IPCC AR5 that there is currently ‘low confidence’ in the results^3^. However, for Europe, as an example, changes in the 100-year and 1,000-year return water levels by the end of the century^36^ can be expected to be (on average) an order of magnitude smaller than uncertainties in the present-day ESL estimates, even under high emission scenarios (here RCP8.5) where changes in the storm surge climate are largest (Supplementary Fig. 5). Locally, and in other regions changes may be more significant, for example due to increased Atlantic hurricane activity^37^. In addition, significant decadal fluctuations in the storm surge climate superimposed onto these long-term trends have been observed in the past^38^,^39^,^40^ and will continue into the future.

In conclusion, our results highlight the necessity to: carefully assess contemporary ESL with appropriate EVA methods, such as GPD99 which is identified here as the preferred approach to parameterize extreme water levels in broad-scale studies; make use of the improved computational capabilities and global reanalysis, and apply advanced dynamical models to simulate coastal sea levels, such as GTSR; extend model hindcasts and continue data archeology^41^,^42^ to obtain long enough time series for the robust estimation of distribution parameters and return water levels; and exploit the rich and constantly growing (in time and space) observational data base, such as GESLA-2 (ref. 19), to remove model bias wherever possible, for example, through spatial bias interpolation. Finally, uncertainties inherent to both future sea-level projections and present-day (and future) ESL estimates need to be understood and combined. Otherwise, the important recent and ongoing improvements in narrowing the uncertainties and providing more robust sea-level projections are in danger of being of little benefit for broad-scale impact and adaptation assessments.

## Methods

### Data processing

Tide gauge data were obtained from the GESLA-2 database^19^. There were often several files for the same site. Sometimes these were provided by the same source and cover different time periods; in these cases we merged the files to obtain the longest possible records. In other cases data was provided by different sources covering similar time periods (often with datum offsets); in those instances we only kept the file with the longer record. Most time series have hourly data but some have higher sampling frequencies. We interpolated all records to hourly resolution, for consistency. Data that were flagged because it contained suspicious outliers or datum shifts etc. were removed before the analysis. We account for non-stationarity resulting from SLR and inter-annual and longer-term sea level variability in a non-parametric way by subtracting the annual average sea level on a year-by-year basis. That is the same approach as used in ref. 14 and preferred over removing linear trends which do not capture variability and acceleration patterns that are evident in the tide gauge records. We also subtract the mean over the last 19 years from the (de-trended) data sets so that our return water level estimates are offset to present-day mean sea level.

### Extreme value analysis

We use three different distribution functions that are commonly applied in (coastal) hydrology and other fields to analyse environmental data sets and are implemented in infrastructure design concepts, namely Gumbel, GEV and GPD^17^. The Gumbel and GEV distributions were fitted to annual maxima time series of total still water levels (GUM-AMAX and GEV-AMAX). The GEV was also used with monthly maxima (GEV-MMAX), as well as time series comprising the 2–10 largest values in each year (GEV-r2, GEV-r3, …, GEV-r10). The GPD is typically used when extreme samples were derived with a peaks-over-threshold approach; here we consider thresholds between the 98th and 99.75th percentiles in 0.25 percentile increments (GPD98, GPD98.25, …, GPD99.75). To assure that all identified extreme events are independent we use a decluster time of 3 days between events, which is the approximate time most storm events influence water levels at the coast^36^,^43^. For all models distribution parameters were derived using the asymptotically optimal Maximum Likelihood Estimator (MLE). Other parameter estimation methods exist, such as the Method of Moments and *L*-Moments, but are not considered here because we assume that for the sample sizes included in our analysis the effects would be negligible compared to the key uncertainties that are discussed and because MLE is superior in most situations^44^.

In total, this leads to a set of 20 representative EVA methods that we apply globally. Additional methods exist that treat tidal and storm surge components separately, for example, the Skew Surge Joint Probability Method^28^ or the approach introduced in ref. 45 where tide and surge values are resampled and then combined to obtain a larger set of extreme events. However, these methods have only been applied and verified in certain regions and are not yet mature enough for global assessments.

At all sites distributions were fitted to the full data sets; we realize that in many regions extreme sea levels can emerge due to both tropical and extra-tropical cyclones and that these should ideally be treated as two populations and analysed separately^29^ or with mixed distributions. However, this is currently not feasible at the global scale due to data and model restrictions, but the development of a global dynamic surge model^14^ was an important first step to achieve this goal.

### Inter-model uncertainties from extreme value analysis

In obtaining inter-model uncertainties from the EVA methods we follow the approach used by the IPCC in its 5th Assessment Report (AR5) to allow direct comparison of future SLR and present-day ESL uncertainties. For a range of relevant return periods we obtain the associated extreme water levels with the EVA methods outlined above and fit a Gaussian distribution from which we calculate the 90% confidence levels (or 5–95% ranges).

### Uncertainties imposed by the length of available data sets

Uncertainties that arise from the available record lengths are assessed at tide gauge sites with at least 70 years of (nearly) complete data, that is, 43 sites in total. The GPD99 method is first used to calculate return water levels from the 70-year long data sets and the results from this are used as baseline or ‘reference truth’. Next, the time series are shortened by one year each time step and changes in the return water levels are assessed, until only the most recent 20 years of data are left.

### Combining uncertainties

Throughout the paper different types of uncertainties (for example, 5 to 95% ranges in present-day ESL estimates and SLR projections) are combined using the root of the sum of squares (RSS) as follows:


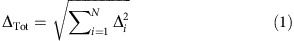


Where Δ_Tot_ is the combined uncertainty and *N* is the number of individual uncertainties Δ_*i*_ that are combined.

### Data availability

The data used in the present study is publically available from http://www.gesla.org/ and doi: 10.4121/uuid:aa4a6ad5-e92c-468e-841b-de07f7133786, and from the corresponding author on reasonable request.

## Additional information

**How to cite this article:** Wahl, T. *et al*. Understanding extreme sea levels for broad-scale coastal impact and adaptation analysis. *Nat. Commun.* **8,** 16075 doi: 10.1038/ncomms16075 (2017).

**Publisher’s note:** Springer Nature remains neutral with regard to jurisdictional claims in published maps and institutional affiliations.

## Supplementary Material

Supplementary Information

Peer Review File

## Figures and Tables

**Table 1:** Global mean uncertainties in future sea level rise projections and present-day extreme sea level estimates.

	**Component**	**Source**	**RCP2.6**	**RCP4.5**	**RCP8.5**
Uncertainties in future global SLR projections (cm)	Thermal expansion*	Atmosphere–Ocean General Circulation Models	8	9	12
	Land water storage*	Hydrological models	10	10	10
	Glaciers*	Glacier models	12	13	14
	Greenland ice sheet surface mass balance*	Ice sheet models	6	8	13
	Antarctic ice sheet surface mass balance*	Ice sheet models	4	4	6
	Greenland ice sheet rapid dynamics*	Ice sheet models	5	5	5
	Antarctic ice sheet rapid dynamics*	Ice sheet models	17	17	17
	Total SLR projection†		**29**	**31**	**37**
					
Uncertainties in present-day ESL estimates (cm)	Tide and surge	EVA method‡ 10|100|1,000 years	7|22|60		
	Tide and surge	Record length§ 10|100|1,000 years	3|7|13		
	Tide and surge	Hydrodynamic model|| 10|100|1,000 years	26|33|41		
	Total present-day ESL estimates¶	10|100|1,000 years	**27|40|74**		

The 5 to 95% range in each component and total future global sea-level rise (SLR) in 2081–2100 under the Representative Concentration Pathway (RCP) 2.6, RCP4.5 and RCP8.5 scenarios is compared to (scenario independent) uncertainties in present-day extreme sea level (ESL) estimates.

^*^Global mean 5 to 95% inter-model uncertainty range in projections of different sea level components (ref. 3; Table 13.5).

^†^Uncertainties in total SLR projections (ref. 3; Table 13.5); because of the way uncertainties are treated this is not the root of the sum of squares of uncertainties in individual components and also not the mean of the regional uncertainties shown in Fig. 2b (see ref. 3 for details).

^‡^Global mean 5 to 95% inter-model uncertainty range in ESL estimates for different return periods.

^§^Average uncertainty across 43 sites for different return periods when using 35 years of data (ie, the length of the Global Tide and Surge Reanalysis (GTSR) instead of 70 years.

^||^Mean absolute differences in ESL estimates from observations and GTSR for different return periods.

^¶^Combined (quasi-)global average (from 510 and 43 tide gauges, respectively) uncertainties in present-day ESL estimates. Different types of uncertainties were combined using the root of the sum of squares (Methods section). Bold entries represent total uncertainties.
