# Supplementary material for: Understanding extreme sea levels for broad-scale coastal impact and adaptation analysis
Source: Nat Commun. 2017 Jul 7;8:16075. doi: 10.1038/ncomms16075 (PMC5504349; doi:10.1038/ncomms16075)
Supplement: Supplementary Information [file ncomms16075-s1.pdf]

File name: Supplementary Information
Description: Supplementary Figures

File name: Peer Review File
Description:

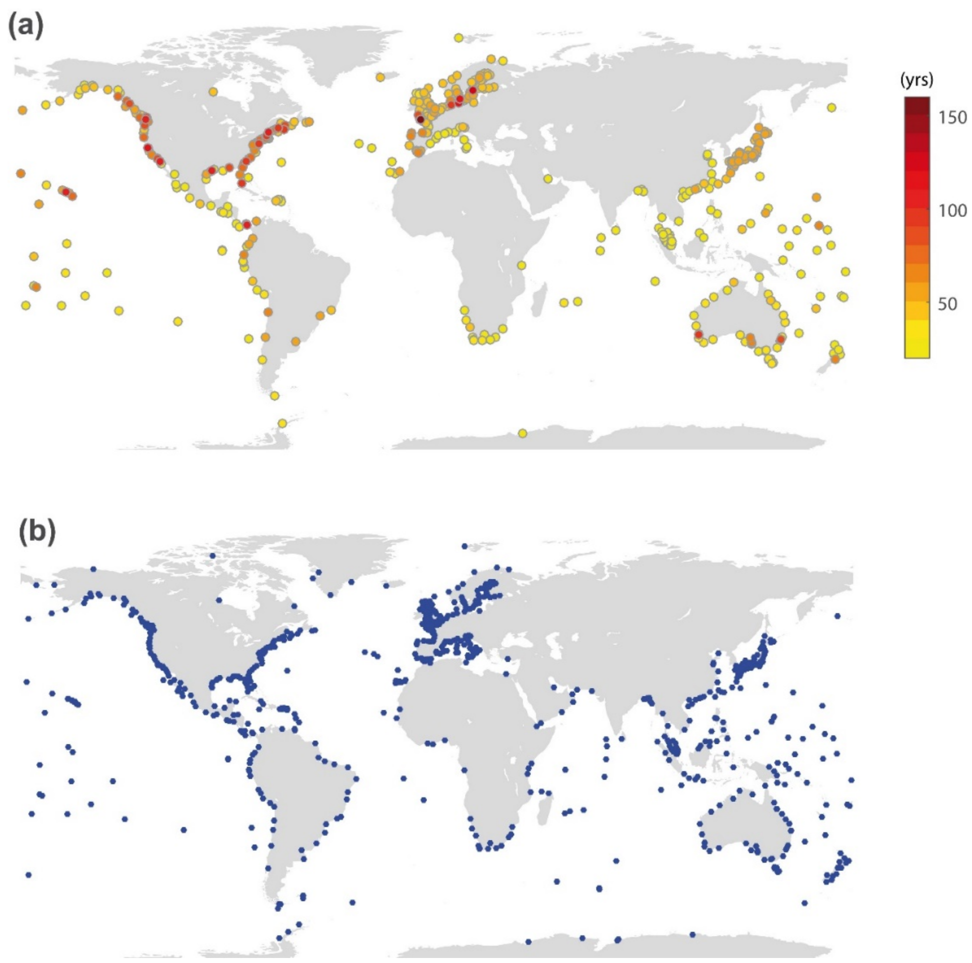

Supplementary Figure 1 | Data availability from the GESLA-2 data base
(a) Length of tide gauge records at sites with at least 20 years of nearly complete data (i.e., at least 75% of hourly values available in each year). **(b)** Locations of sites with at least 5 years of data.

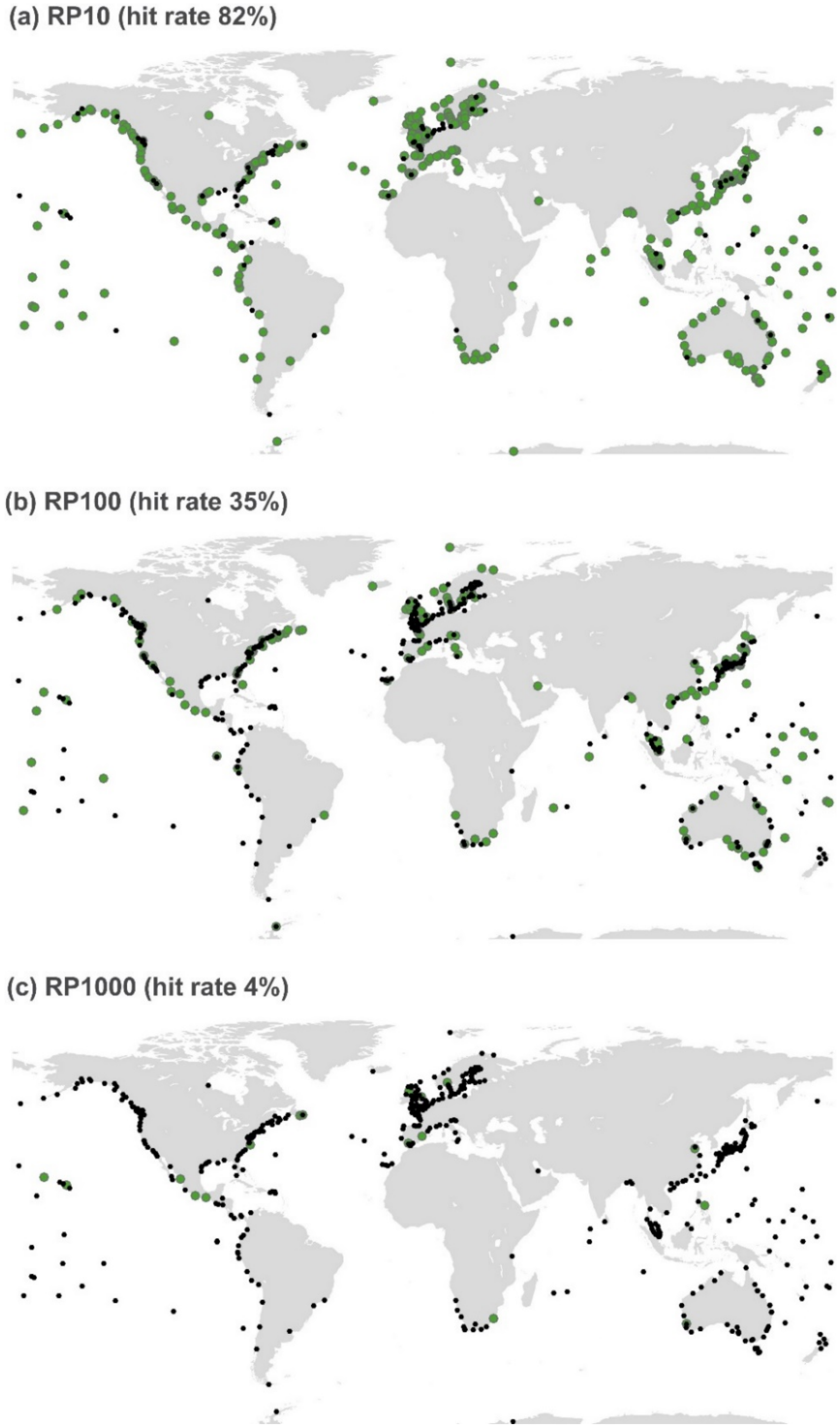

Supplementary Figure 2 | Comparison of return water levels derived with different extreme value methods

Hit rates (green) where the results from all extreme value methods lie within the 95% confidence levels of the Gumbel annual maxima (GUM-AMAX) method for the **(a)** 10-year, **(b)** 100-year, and **(c)** 1,000-year events. Black dots denote locations where results from at least one extreme value analysis (EVA) method lie outside the GUM-AMAX confidence levels.

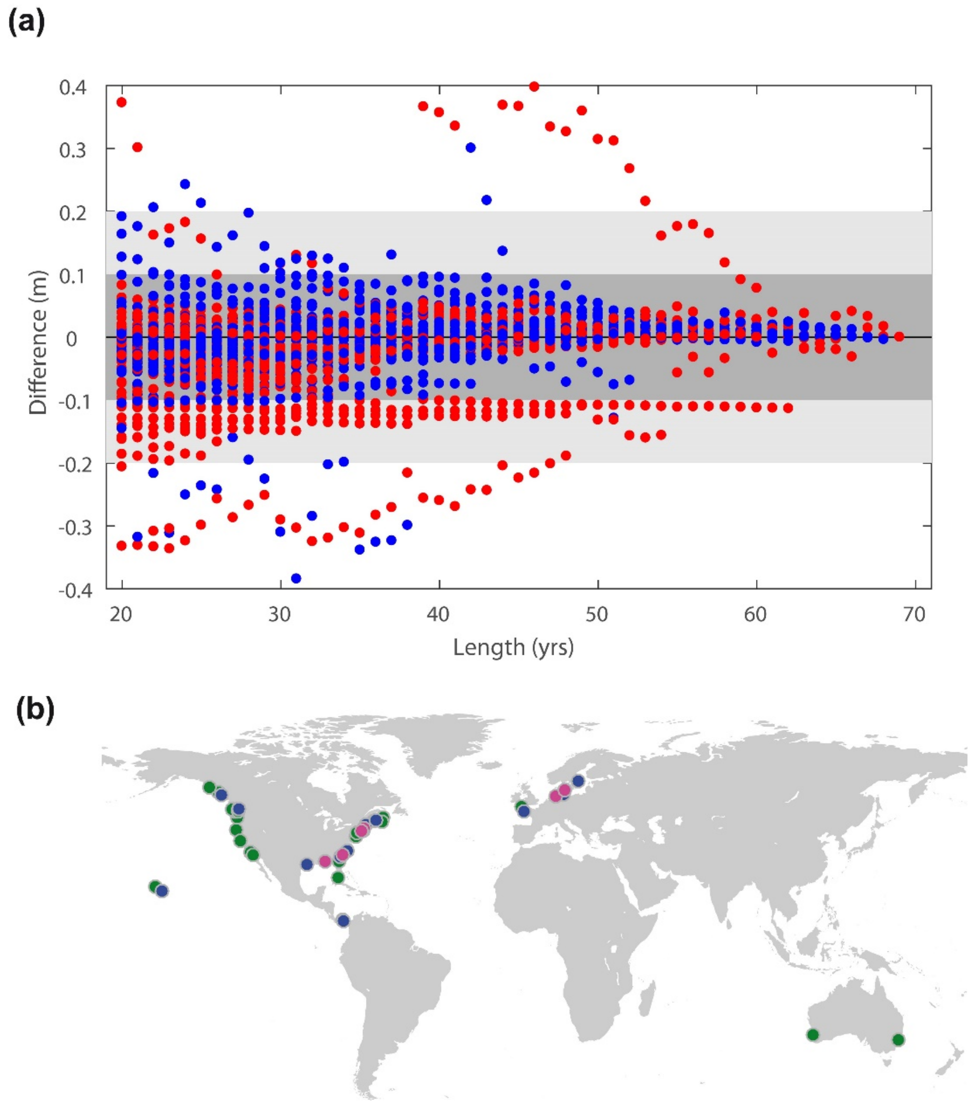

Supplementary Figure 3 | Impact of record length on return water level estimates

(a) Relative changes in the 100-year return water levels when time series are shortened by one year each time step, starting with 70 years and stopping at 20 years. Only tide gauges with at least 70 years of data are considered; blue dots indicate that 95% confidence intervals obtained for the full records lie within the 95% confidence intervals of the truncated data, red dots indicate they lie outside; shaded bands represent errors of 10 and 20 cm. **(b)** Tide gauges where the relative changes in the 100-year return water levels never exceed 10 cm (green), never exceed 20 cm (blue), and exceed 20 cm for at least one time step (purple).

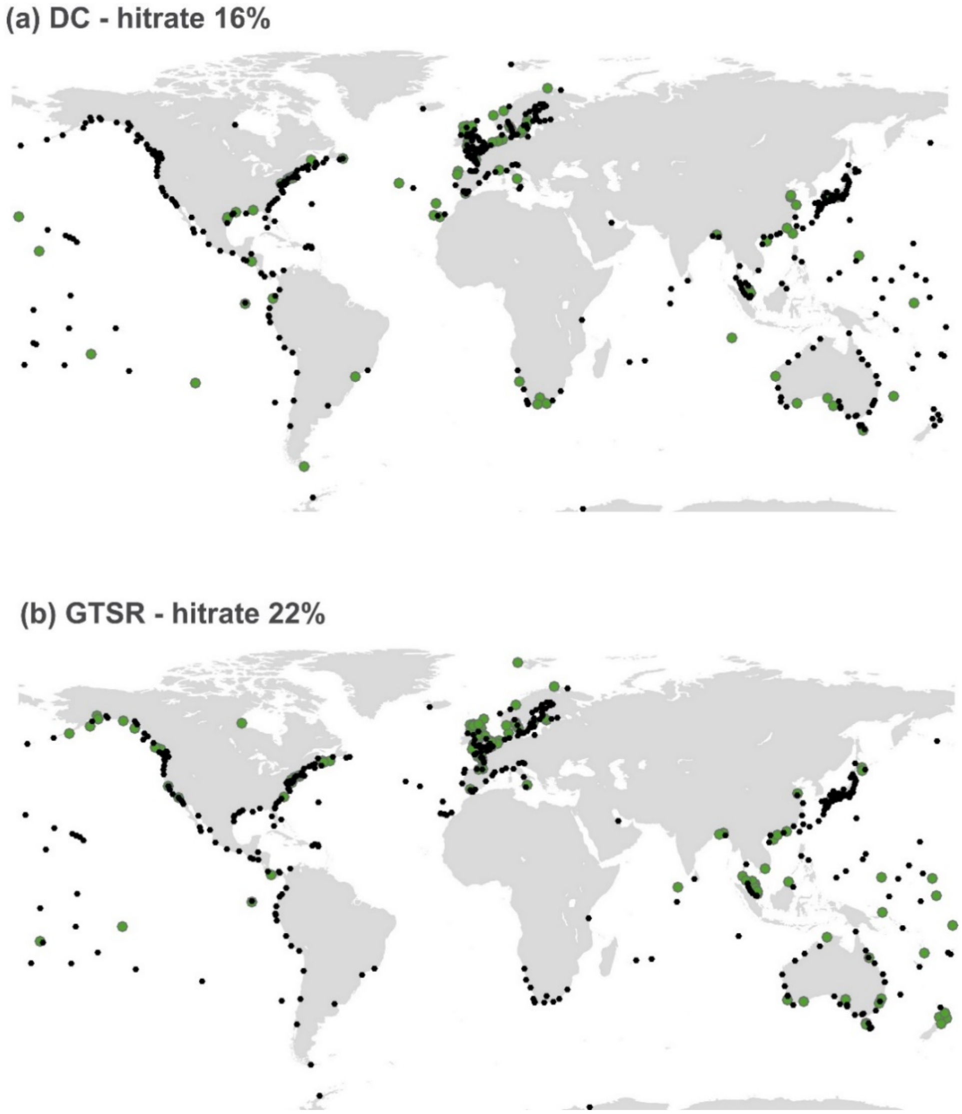

Supplementary Figure 4 | Comparison of return levels obtained from observations and models
Hit rates (green) where the 100-year return water levels obtained with **(a)** D-C and **(b)** GTSR lie within the 95% confidence levels of the estimates from the observations (GUM-AMAX approach). Black dots denote locations where D-C or GTSR results lie outside the GUM-AMAX confidence levels obtained from analysing observed data.

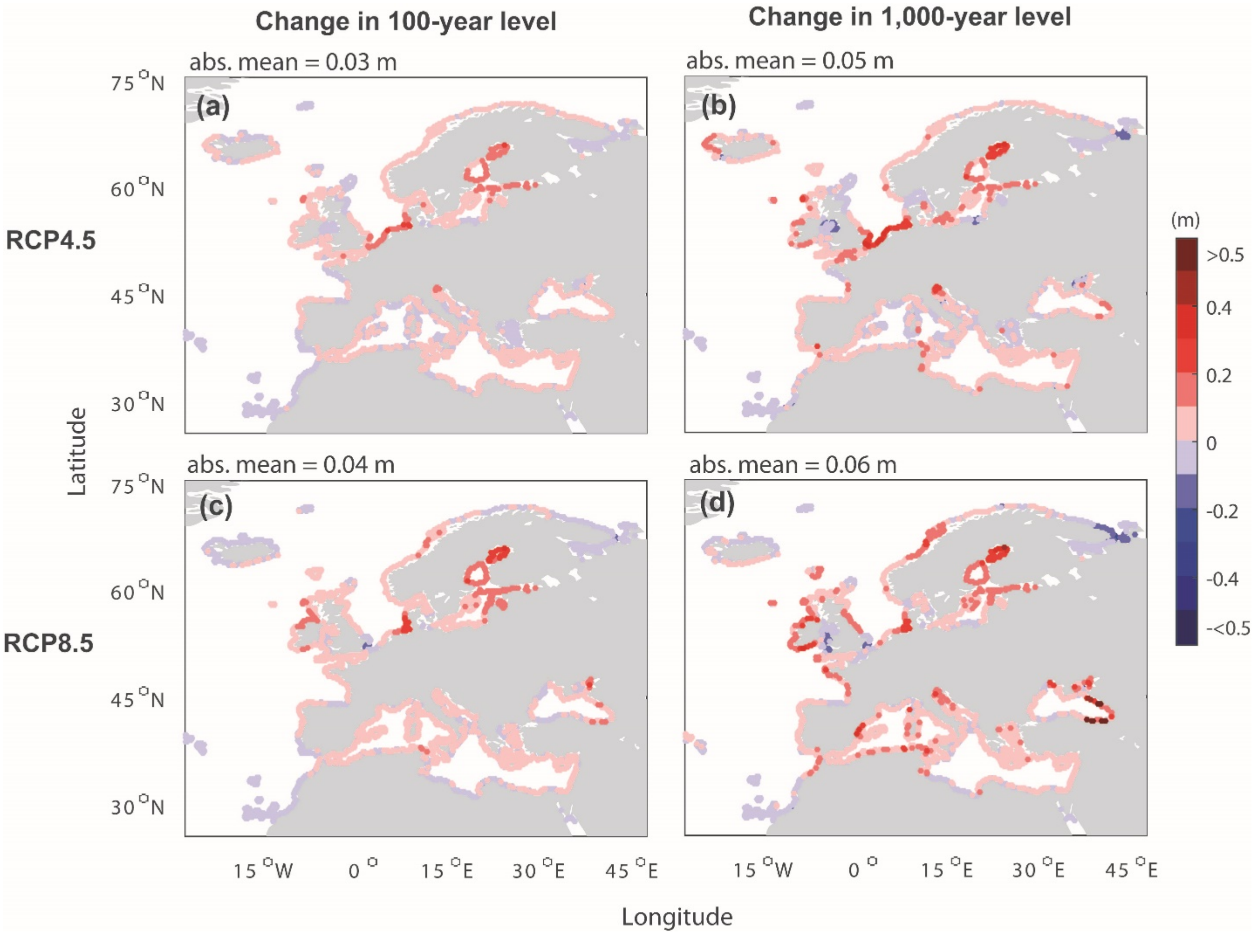

Supplementary Figure 5 | Future changes in return water levels (as derived in ref. 36)
(a, c) changes in the 100-year storm surge return water levels in Europe under the RCP4.5 (a) and RCP8.5 (c) scenarios. **(b, d)** changes in the 1,000-year storm surge return water levels in Europe under the RCP4.5 (b) and RCP8.5 (d) scenarios. Mean absolute differences are listed above the respective panels.
