# Supplementary material for: Understanding extreme sea levels for broad-scale coastal impact and adaptation analysis
Source: Nat Commun. 2017 Jul 7;8:16075. doi: 10.1038/ncomms16075 (PMC5504349; doi:10.1038/ncomms16075)
Supplement: Peer Review File [file ncomms16075-s2.pdf]

Reviewers' Comments:

Reviewer #1 (Remarks to the Author)

Overall Review

The manuscript address a very relevant question - the quantification of trends in GPP and WUE occurred during the last three decades (1982-2011) and the attribution of the trend to specific factors e.g., increase in CO2, changes in L (leaf area index), D (vapor pressure deficit) and the ratio between evaporation from canopy interception and total evapotranspiration. While trends in WUE and to a less extent GPP have been already analyzed in several articles (Saurer et al. 2004; Keenan et al. 2013; Frank et al 2015; Xue et al. 2015), here the authors used an analytical framework (Equation (1)) and a combination of various databases from remote sensing observations, reanalysis, upscaled products (e.g., MTE) and land surface models to infer WUE directly and compute GPP as the product of E (evapotranspiration) and WUE. The manuscript is well written and presented. The quantification of global scale GPP, WUE and their respective trends is a particularly interesting topic and the magnitude of the presented trends (Line 97, 101, 103) is in agreement with our understanding of the biosphere response to climate change.
All the results follow directly from the derivation of equation (1). Therefore, insofar equation (1) is accepted all the results (Fig. 2, 3 and 4) can be derived in a straightforward manner. The trend in E is weak (Line 101), changes in fe are insignificant (Line 116), thus all changes in WUE and GPP should be related to trends in Ca, L and D and mostly to Ca, which underwent the most clear and undebatable positive trend in the last 30 years.

Said that, equation (1) is derived under a series of restrictive assumptions (Supp. Mat. Line 31-44): (i) the partitioning of transpiration and soil evaporation follows Beer's law, while in reality the problem is much more complex (e.g., Schlesinger and Jasechko 2014); (ii) stomatal conductance and net assimilation are linked using a very specific model, (iii) the parameter k and g1 are static, and most important (iii) ecosystem GPP and E are approximated with leaf-scale GPP and E (Line 45-46, Line 244 and Line 32-34 in Supp. Material). This last assumption is likely the most restrictive because for instance in Eq. (1) a change in leaf area index would uniquely reflect in the partition between evaporation and transpiration and not on GPP directly. The model is also limited because cannot account for changes in internal CO2 concentration (Ci), which is implicitly assumed to be always proportional to Ca, the reason why the result that WUE and Ca scale equally (Line 213) is implicit in the methodology.
All these assumptions left me quite doubtful on the real effectiveness of Eq (1) but I would be very satisfied if Eq. (1) will work well despite these assumptions. Unfortunately the validation of the model does not fully dissipate my doubts. The model is able to explain less than 50% (R2=0.46, Line 66) of the variability in the observed WUE at 123 eddy covariance tower (Figure 1a), the mean trend in ecosystem WUE is almost twice (1.85) larger than the observed trend (Line 68-70) and the mean global WUE is 1.26 times larger than estimates from Land surface model and MTE (Line 78-80). The magnitude of the trend in GPP and WUE also differ considerably from the study of Keenan et al. 2013. Keenan et al. 2013 found, based on flux-tower observations, a 4.6% per 1% increase in Ca (Medlyn and De Kauwe, 2013) and not 1% per 1% as the authors wrote in Line 214.

I am well aware of the considerable uncertainties in the observations and I would not expect or ask for any perfect match but I am not sure the validation can be considered satisfactory to the point Eq. (1) can be used for all the subsequent discussion. In this article, validation is more important than in other contexts (e.g., the land surface models of Fig. S6) because the model is an analytical derivation and does not have any mechanistic structure on it. I would urge the authors to give more compelling motivations why the reader should trust the results despite all these issues and how/why their results differ from previous estimates of trends in WUE and GPP. Are the

trends in GPP and WUE expected to be less sensitive to the assumptions in Eq. (1) then the magnitude themselves? Are these trends robust to the scatter of Fig. 1a. Maybe yes, but this argument needs to be explicit in the paper. Please also note that Eq. (1) is a simplification of our current understanding of WUE, while the unexplained variability could be in fact the most important aspect of changes in WUE, the one we still not fully understand and that may provide a future picture quite different from what we expect. I think this aspect needs a different emphasis in the article.

Specific comments

Line 21. I would use a "may have consequences" since there is still some debate on the effect of elevated CO2 in affecting runoff and streamflow (e.g., Huntington 2008).

Line 35. Beyond soil, I would also suggest to mention forest demography, nutrients, and atmospheric feedbacks.

Line 40-42. This sentence is probably too optimistic, I concur that estimates of E from precipitation and streamflow, if the two above variables are reliably measured, can be quite accurate in the long-term (E of multiple years). However, at the annual scale it is likely not the case because changes in storage in the basin (groundwater, soil, eventually snow) can affect the computed E and E trends. Differences among products in Fig. S2 are supporting my concern.

Line 88-89. I guess there is a bit of circular reasoning, the spatial patterns of the computed GPP should naturally follow the one of the products used to compute Eq. (1), which includes MTE and land surface model simulations. Therefore, the agreement between GPP spatial details should not be used as a mean of validation (Fig. S7).

Line 130, 131, 134 and 135. A minor detail, I found a bit confusing that the percentage of significant trends is not given on the total but on the subset of positive or negative trends.

Line 213-214. I believe this sentence is wrong, Keenan et al. 2013 found a much stronger trend in WUE rather than proportionality with Ca, actually this is the overall point of their article and the reason why it was published in Nature.

Line 230-232. This sentence is repetitive with what you stated previously.

Line 259-260. Is Ca used as spatial uniform in the entire Earth? Or geographical differences are preserved?

Line 287-290. The explanation on the partition of the different contributions remains unclear to me. Only reading the Supp. Material I could understand what the authors did.

Figure (4). In the caption, it should be "leaf area index (e)"

References

Frank, D. C. et al. (2015), Water-use efficiency and transpiration across European forests during the Anthropocene, Nat. Clim. Chang., 5, 579-583, doi:10.1038/nclimate2614.

Xue, B.-L., Q. Guo, A. Otto, J. Xiao, S. Tao, and L. Li (2015), Global patterns, trends, and drivers of water use efficiency from 2000 to 2013, Ecosphere, 6, 174,

Keenan, T. F., D. Y. Hollinger, G. Bohrer, D. Dragoni, J. W. Munger, H. P. Schmid, and A. D.

Richardson (2013), Increase in forest water-use efficiency as atmospheric carbon dioxide concentrations rise, Nature, 499, 324-7, doi:10.1038/nature12291.

Saurer, M., R. T. W. Siegwolf, and F. H. Schweingruber (2004), Carbon isotope discrimination indicates improving water-use efficiency of trees in northern Eurasia over the last 100 years, Glob. Chang. Biol., 10, 2109-2120, doi:10.1111/j.1365-2486.2004.00869.x.

Schlesinger, W. H., and S. Jasechko (2014), Transpiration in the global water cycle, Agric. For. Meteorol., 189-190, 115-117.

Medlyn, B., and M. De Kauwe (2013), Biogeochemistry: Carbon dioxide and water use in forests, Nature, 499, 287-289, doi:10.1038/nature12411.

Huntington, T.G., 2008. CO2-induced suppression of transpiration can not explain increasing runoff. Hydrol. Process. 22, 311-314.

Reviewer #2 (Remarks to the Author)

Review of Recent increases in terrestrial carbon uptake at little cost to the water cycle for Nature Communications.

The paper describes a model of optimal stomatal conductance used to estimate ecosystem water use efficiency (WUE). The stomatal conductance equations and parameters have been applied lately to land surface models, but this is a novel application in that several observation datasets are used as inputs in the model to predict global WUE. Based on seven datasets of global evaporation (E), an ensemble of global GPP is generated. This is used to show increases in GPP since 1982, due to increases in WUE and leaf area index (L).

The strength of this paper is the application of the optimality model, which has recently been used and suggested as an improvement over previous empirical stomatal conductance models. Another strength is the large number of datasets used to give an uncertainty range, which is key to include for this difficult to estimate value. This is a potentially very useful dataset and model of WUE.

Although I think the paper could make a valuable contribution, I can see a few weaknesses, which might make the paper unsuitable for Nature Communications in its current form. I will explain further here and provide suggestions when possible.

Major Comments

1. Pertaining to ability of the WEC model to reproduce observed trends in WUE.

I am not fully convinced that the model is able to capture the observed trends in WUE at Fluxnet sites. The observed mean trend is 20.4±7.4 mg C mm-1 H2O year-1, and I disagree that this is "consistent" with the model-predicted trend of 37.0±2.7 mg C mm-1 H2O year-1 (Lines 67-70), as the latter is nearly twice as high as the observations.

One problem is the significance of the trends shown in Figure 1b. It's very difficult to tell how much to trust the trend lines given the large variation in individual sites. So can we even say with confidence that WUE at the sites is increasing? And the same for the WEC model results? For the sites shown in Figure S5, it would be useful to include the slope of the trend lines and whether they are significantly different from zero.

Also, in the SI, it is stated that WUE increased by 35.9±23.0 mg C mm-1 H2O year-1 in the model and by 13.6±20.2 mg C mm-1 H2O year-1 in the observations (Lines 150-154). Why are these numbers different from those provided in the main text?

I think there are important implications of the WEC model overpredicting increase in WUE over the

study period. This means the predicted increases in GPP are possibly to high. A useful paper to compare to is Large divergence of satellite and Earth system model estimates of global terrestrial CO2 fertilization by WK Smith et al. They found many more regions of decreased NPP than found for GPP in this study (obviously you cannot make a direct comparison between NPP and GPP, but it's likely the large-scale patterns of change should be similar). Another check could come from the GPP based on upscaled Fluxnet data – are trends in GPP similar?

Another question I have is: How sensitive is the WEC model to the leaf area index, L? The assumption behind Eq. 5 is that ET dominates for high LAI canopies and decreases quickly as L decreases. Is there any data to back up this statement, or is it possible that the effect of L saturates quickly, so for high L it doesn't really matter (once the ground is covered by a tree, the evaporation from soils will not easily get into the atmosphere whether the tree has L of 4 or 7, for example). To me this is important, because the increases in L over the study period mean that increases in D are overwhelmed, so the WUE increases. However, this seems to be in contrast to the findings of Smith et al 2015, where NPP sensitivity to VPD was shown to increase over the same time period (1982-2011).

Minor comments

1. Lines 80-82: It would be useful to summarize in 1-2 sentences the broad spatial patterns of WUE. Also, there is a large divergence in global patterns of WUE from the land surface models shown in Figure S6 (especially in high latitudes).

2. Lines 140-142: Are panels c and d switched in Figure 3? In the figure, there is a large decrease in GPP in central Africa, but the text states the opposite.

3. Lines 183-184: This is the first time the parameter g1 is mentioned in the main text, I think it should be explained a bit so readers do not necessarily have to go through the SI find out what it is.

4. Lines 207-209: This suggests that tropical forest GPP is very sensitive to Ca. A recent study also found large increases in tropical tree WUE, but no concurrent increases in tree growth (van der Sleen et al. 2014). However as both of these studies suggest large increases WUE, the relationship between these results would be worth including in the discussion.

4. Lines 211-212: Can the observed effect on NPP from FACE be explicitly stated?

5. The assumption in moving from Eq. 1 to Eq. 3 (in the SI) is valid for the growing season only – are all model equations only calculated during the growing season as well?

6. There is a similar paper which was recently published in ESD by Dekker et al. Using a different approach to calculating WUE, the authors found larger changes in fractional WUE over the 20th century than suggested by the optimality approach used in this study. It would be interesting to know how these results fit in the context of the sensitivities found in that study, which also used tree ring data.

References:

van der Sleen et al 2014: No growth stimulation of tropical trees by 150 years of CO2 fertilization but water-use efficiency increased, Nature Geoscience, DOI: 10.1038/NGEO2313

Smith et al 2015: Large divergence of satellite and Earth system model estimates of global terrestrial CO2 fertilization, Nature Climate Change, DOI: 10.1038/NCLIMATE2879

Dekker et al 2016: Spatial and temporal variations in plant water-use efficiency inferred from tree-ring, eddy covariance and atmospheric observations, Earth System Dynamics, DOI: 10.5194/esd-

7-1-2016.

Reviewer #3 (Remarks to the Author)

The manuscript is original and presents an interesting new approach. WUE as an emergent ecosystem property is getting a lot of exposure lately (see also Reichstein et al 2014 PNAS, which, as a side note, should be referenced in this manuscript), and this manuscript provides an elegant methodology to use WUE as a predictive variables.

There is an argument for and against the approach taken here. There could be something intrinsic and physically driven in WUE, and if that is the case the approach presented here could be extremely useful. However it is also possible that WUE is the emergent result of multiple plant and meteorological processes that couple the atmosphere to canopy transpiration and carbon uptake. If that is the case, any model that is made to fit the current observed WUE is essentially over-fitted because it ignores all the independent processes whose dynamics could change in complex ways as a result of climate change.

In the introduction the authors present the climate change and carbon fertilization responses of global vegetation (L17 and L22) as these are well known and accepted and reference 3 papers, where, in fact, there is a large debate and many papers each year provide the results of many long-term observation and synthesis papers and large scale FACE experiments with no uniform conclusion. You should revise this section, to emphasize the lack of consensus, and then your next sentence will actually make sense "Therefore, quantification of the response of the water and carbon cycles − which are closely coupled as both diffuse through leaf stomata − to global warming and rising Ca is very complex, and much needed."
[Notice that I edited that sentence somewhat, it was badly phrased and had some grammatical errors].

One key problem I have with this approach is the assumption that (L40-42) "On the contrary, ecosystem water use, i.e. E, which can be estimated from catchment hydrological observations (including precipitation and streamflow), is quite accurate." I totally do not agree that E estimated from catchment hydrological observations is "quite accurate". I have seen many, and in my experience they are all far from accurate. In fact, the runoff and soil moisture are the least observed variables from remote sensing, and there are very few satellite product and very coarse datasets for these, as compared for example to vegetation greenness and GPP that is derived from it. It is not obvious that a method based on estimated E values has an upfront advantage relative to other methods that are based on vegetation greenness.

And an editorial side comment to this sentence (L40-42), it is not clear what this is "on the contrary" for? Contrary to estimates of ecosystem GPP? Contrary to global scale estimates of WUE?

The validity of the analytical WUE model – I am impressed by the r^2 of 0.68 of the annual WUE, tough Nash-Sutcliffe model efficiency of 0.34 is not so great. I would not predict an r^2 as high even for the comparison between the mean 0.5x0.5 deg E from the global data sources you used and direct observations of evapotranspiration from the flux towers (this will be a nice exercise to check).

The high r^2 is particularly surprising given that the method used for its calculation of WUE is extremely simplistic. It neglects canopy structure (k is constant), reduces plant heterogeneity to a single variable g1 and driven by the SYNMAP PFTs. In my experience, most flux sites have their footprints over vegetation composition very different than the generalization of SYNNMAP for that are, and I know that WUE is variable between species of the same PFT in the same site. Your formulation also neglects stomata dynamics which determine when and at what D water is evaporated and carbon is assimilated, with big differences between days of wet/dry soil. A change

in stomata opening toward the morning (a common effect of dry soil) can reduce WUE as more carbon uptake is done earlier in the day when radiation is limiting. Finally it is driven by highly inaccurate estimates of E. I am also confused as to what D you are using? The mean monthly D? There are strong diurnal fluctuations in D and the values that are relevant to WUE are those present during the time the plants are actively transpiring.
A small "cheat" is using the observed D for the site comparison. It makes sense, however, it hinders the validation of the global approach. If you had used the 0.5x0.5 deg D from the global datasets, how much would it reduce the accuracy of the WUE estimation?
Another cheat is throwing all the sites where WUE is high. I accept removing sites with observation problems, such as the sites with energy closure of less than 75%. But why kick out all the sites with high WUE? This will directly affect the bias of your model and make it look better than it is.
Given all that, I want to see more to be convinced that your formulation actually predicts the correct WUE for the correct reasons.
You write in the appendix that WUE is calculated monthly, but you only show the annual means. How does the Monthly fair?

Most importantly, how does your model do in predicting the change in WUE? In sites where there is a long-term trend in WUE, did your model find this trend? Over all sites you report a predicted increase of WUE 37+-2.7 per year, and claim it fits an observed 20+-7.4 per year. I do not agree. Your estimate is far out of the range, and almost twice as large as the observation. I conclude that your model has a large bias in the trend of WUE, much larger than its bias in the mean WUE. There could be many reasons for this. I would suspect missing mechanisms. Models could be over parameterized and represent the means during a certain time very well but will fail predicting long term trends. Specifically, can you translate figure S5 to a scatterplot of the observed vs predicted slopes of WUE change over time? In at least 4 of the sites you predict the opposite trend to the observed. I am curious as to how it will look overall.
Similarly, I want to see a validation of E. All flux sites directly measure E, but you use E from global datasets. Are these anything close to the flux sites? Do they capture the long term trends in E as observed in the flux sites?
A related question – how did you choose the sites?
I know many more sites in the Ameriflux dataset that have more than 7 years of data. But they are not used for the trend evaluation. Why?

It will be reasonable to add in one supplement a cite table listing all the sites you used, their lat-long, the period of data they have (only years with high data quality that were actually used in the analysis), what dataset you got them from (Ameriflux, Fluxnet, ChinaFlux…), what was the vegetation PFT you assigned to it (g1). Without this information it'll be impossible to repeat the work you did.

Finally, the manuscript is written rather poorly. I am not a native English speaker and tend not to complain about language, but even I got disturbed by the many grammatical error and awkward sentencing throughout.

I was editing the grammar but gave up after 2 pages.
Here are some corrections that I suggest:

L9 does not commensurate
L11 per unit of water used (i.e. water use efficiency).
L12 canopy leaf area index, and negatively
L13 suggest that rising atmospheric
L16 Global climate change, caused mainly by
L21 consequences to the hydrological cycle
L24-25 Understanding how the coupled water and carbon cycles will change under future climate and rising

L28-31 Ecosystem WUE, defined as GPP per unit ecosystem water loss via E, is one of the most important functional properties of ecosystems that drive terrestrial carbon and water exchanges with the atmosphere4,5,15. Analytical models based on the optimization theory can explain leaf WUE, defined as leaf photosynthetic carbon uptake per unit water loss via transpiration, variations with plant function types and climate16,17. However, understanding of ecosystem WUE is still very limited5,15,18,19.

L36-30 It is really hard to figure out what you are trying to say here. What is "prior"?

L50 studies on WUE (e.g. 5,15,19) [ref numbers in superscript]

L52 not requiring prior estimates

Responses to reviewer #1:

Overall Review

The manuscript address a very relevant question - the quantification of trends in GPP and WUE occurred during the last three decades (1982-2011) and the attribution of the trend to specific factors e.g., increase in CO2, changes in L (leaf area index), D (vapor pressure deficit) and the ratio between evaporation from canopy interception and total evapotranspiration. While trends in WUE and to a less extent GPP have been already analyzed in several articles (Saurer et al. 2004; Keenan et al. 2013; Frank et al 2015; Xue et al. 2015), here the authors used an analytical framework (Equation (1)) and a combination of various databases from remote sensing observations, reanalysis, upscaled products (e.g., MTE) and land surface models to infer WUE directly and compute GPP as the product of E (evapotranspiration) and WUE. The manuscript is well written and presented. The quantification of global scale GPP, WUE and their respective trends is a particularly interesting topic and the magnitude of the presented trends (Line 97, 101, 103) is in agreement with our understanding of the biosphere response to climate change.

All the results follow directly from the derivation of equation (1). Therefore, insofar equation (1) is accepted all the results (Fig. 2, 3 and 4) can be derived in a straightforward manner. The trend in E is weak (Line 101), changes in fe are insignificant (Line 116), thus all changes in WUE and GPP should be related to trends in Ca, L and D and mostly to Ca, which underwent the most clear and undebatable positive trend in the last 30 years.

A1. Said that, equation (1) is derived under a series of restrictive assumptions (Supp. Mat. Line 31-44): the partitioning of transpiration and soil evaporation follows Beer's law, while in reality the problem is much more complex (e.g., Schlesinger and Jasechko 2014);

Responses: We agreed that our equation (1) was derived based on a number of assumptions, which have been clearly stated in the supplementary materials, and noted by the reviewer. As stated in our eqn (1), the relationship between T/Et and *L* also depends on C_a, D, g_1 and k.

Findings from Schlesinger and Jasechko (2014) do not contradict our predicted dependence of T/ET on canopy leaf area index[1]. They found that T/ET was higher in high-LAI ecosystems, such as tropical rainforest than the low-LAI ecosystem, such as desert and shrub land, which is broadly consistent with our model predictions.

We agreed that partitioning of transpiration and soil evaporation is much more complex than Beer's law in reality, especial at short times scales (i.e. hourly or diurnal scales). However, this study is focused on monthly to annual time scales, at which Beer's law can provide reasonable and accurate partitioning between transpiration and soil evaporation (see Schulze et al. (1994)[2], Kelliher, et al. (1995)[3] and Wang et al., (2014)[4]).

We thanks the review for this insightful comment. In the revised manuscript, a short discussion on water use partitioning is provided in the supplementary material (see lines 51-55 in the Supplementary Section 1).

A2. (ii) stomatal conductance and net assimilation are linked using a very specific model,

Responses: The stomatal conductance model used in this study is similar to the Ball-Berry stomatal conductance model that has been widely adopted in most global land surface models. What is different is that parameters in our stomatal model have a meaningful ecological interpretation as stated by Medlyn et al. (2011)[5]; and were estimated using extensive field observations by Lin et al. (2015)[6], whereas the two empirical parameters in the Ball-Berry model as used in most global land models often have constant values globally for C3 or C4 plants.

We thanks the review for this insightful comment. A short discussion on stomatal conductance model is provided in the revised supplementary material (see lines 42-46 in the Supplementary Section 1).

A3. (iii) the parameter k and g1 are static,

Responses: The parameter *k*, i.e. light extinction coefficient within the canopy, is essentially related to vegetation canopy structure and leaf properties. Uncertainty of *k* within the range of 0.4 to 0.8 only has a small effect on the estimated trend of WUE, thus *k* is set as constant to keep a simple parameterization of the WUE equation.

Different values of g_1 used in this study capture the broad differences among different plant functional types, as stated by Lin et al. (2015)[6]. Lin et al. (2015)[6] also demonstrated a significant relationship (r^2 = 0.43)[7] between g_1 and two bioclimatic variables (i.e. moisture index and surface air temperature). We compared two different estimates of g_1 (including or excluding the additional dependence of g_1 on soil moisture index and surface air temperature), and found that the estimates of ecosystem WUE (mean value, spatial pattern and trend) are not significantly different. Furthermore, we chose not to include the additional dependence of g_1 on soil moisture index and surface air temperature in this study because (1) the relationship of g_1 with moisture index and temperature is not very robust (see Figure 4 of Lin et al. (2015)[6]); (2) to keep a simple parameterization of the WUE equation. In the revised manuscript, further discussion on different schemes for mapping global g_1 pattern is provided in the supplementary materials (Section 6.1).

A4. and most important (iv) ecosystem GPP and E are approximated with leaf-scale GPP and E (Line 45-46, Line 244 and Line 32-34 in Supp. Material). This last assumption is likely the most restrictive because for instance in Eq. (1) a change in leaf area index would uniquely reflect in the partition between evaporation and transpiration and not on GPP directly.

Responses: We scaled leaf-level WUE to canopy scale rather than scaled leaf-level GPP and E to canopy scale individually. Previous studies have shown that leaf WUE is quite independent of the growth environment[8] and can be scaled to individual trees[9, 10]. Thus we approximate the ecosystem transpiration WUE, i.e. GPP/Et, by leaf WUE.

In our study, LAI and ET are two inputs to our model. While it is true that leaf area index (LAI) affects ecosystem WUE by altering the partitioning of ET into E and T and thus WUE. LAI should also affect ET directly (see Schulze et al., (1994)[2], Kelliher, et al., (1995)[3] and Wang et al., (2014)[4]), and their dependence is assumed to be accounted for in the input data.

A5. The model is also limited because cannot account for changes in internal CO2 concentration (Ci), which is implicitly assumed to be always proportional to Ca, the reason why the result that WUE and Ca scale equally (Line 213) is implicit in the methodology.

Responses: It is true that our equation (1) assumes the WUE scales linearly with C_a, yet this is supported by field observations by Barton et al (2012)[10]. The dependence of WUE on C_a also varies with other factors, such as canopy LAI and vapour pressure deficit (D). That is why our trend of WUE increases faster than C_a (1.4% increase per 1% increase in C_a based our global estimate). We state the relationship between C_a and estimated WUE explicitly in the revised manuscript (see lines 233-235).

A6. All these assumptions left me quite doubtful on the real effectiveness of Eq (1) but I would be very satisfied if Eq. (1) will work well despite these assumptions. Unfortunately the validation of the model does not fully dissipate my doubts. The model is able to explain less than 50% (R2=0.46, Line 66) of the variability in the observed WUE at 123 eddy covariance tower (Figure 1a), the mean trend in ecosystem WUE is almost twice (1.85) larger than the observed trend (Line 68-70) and the mean global WUE is 1.26 times larger than estimates from Land surface model and MTE (Line 78-80).

Response: Thank you for the constructive comments on our model. In the revised manuscript, we provide further validation using the latest global flux dataset, i.e. FLUXNET2015, of which data availability is up to 2014 at some stations. Specifically, we use 243 station-years data to validate the model and long-term (greater than 6 years) annual data of 11 stations to validate the trends in annual WUE.

The validity of the model is strongly supported with the updated and expanded dataset. Previous validation of the trend was less satisfactory, primarily because the data used was only up to 2011 and observations of most of flux stations covered shorter periods only. The new validation results show that our model can capture both spatial (validation using all the station-years, Figure 1a) and temporal (trend of stations with long-term available data, Figure 1b) variability of ecosystem WUE observed at flux sites. The spatial validation is as good as in the earlier manuscript, however, the validation of trends is much improved over the previous one. Across the 11 stations with long-term annual data, the estimated mean trend is about 13.7±11.1 mg C mm^{-1} H_2O $year^{-1}$, and is very close to the observed trend of 12.6±11.4 mg C mm^{-1} H_2O $year^{-1}$. The linear correlation coefficient (r) between observed and estimated trends in annual WUE is about 0.74, with Nash-Sutcliffe model efficiency (NSE[11]) of 0.50, root mean squared error (RMSE) of 25.6 mg C mm^{-1} H_2O $year^{-1}$, mean error (ME) of 1.12 mg C mm^{-1} H_2O $year^{-1}$ and relative error (RE) of 8.9%. The updated results strongly support the analytical WUE model (i.e. equation (1) in the main text). We refer the reviewer to Section 3 of the supplementary materials for more details about the new validation.

A7. The magnitude of the trend in GPP and WUE also differ considerably from the study of Keenan et al. 2013. Keenan et al. 2013 found, based on flux-tower observations, a 4.6% per 1% increase in Ca (Medlyn and De Kauwe, 2013) and not 1% per 1% as the authors wrote in Line 214.

Response: We estimated that global WUE increased at a rate of 1.4% per 1% increase in C_a, the greater rate of WUE increase than C_a may result from changes in other factors, such as an increase in canopy LAI[12] and vapour pressure deficit.

The smaller trend in WUE reported in this study than that reported by Keenan et al. (2013)[13] could be resulted from differences in study regions and period of data used. The study of Keenan et al. (2013)[13] focused on the temperate and boreal forested sites only. In this study, we focused on the global scale and we showed that trend in WUE in the forested region is higher than that in other regions (see Figure 3b). This study also differs from Keenan et al. (2013)[13] in different periods of data used, which essentially lead to different definitions of WUE in terms of ecosystem water use. In Keenan et al. (2013)[13], annual WUE was estimated from rain-free summer days only and thus soil evaporation and canopy interception were excluded from analysis. While we used all year data and considered two more types of ecosystem water uses than that of Keenan et al. (2013)[13], i.e. soil evaporation and canopy interception (see equation (1) and Supplementary Section 1). Using the observed flux data from a close canopy site (AU-Tum), we demonstrated that annual trends in WUE are different by using different periods of data (see Figure R1 below). Figure R1 shows that annual trend in WUE at the AU-Tum site is about 13.0±2.2 mg C mm^{-1} H_2O $year^{-1}$ estimated from rain-free summer days only as in Keenan et al. (2013)[13], which is about two times larger than that (6.6±3.1 mg C mm^{-1} H_2O $year^{-1}$) estimated from the whole year data.

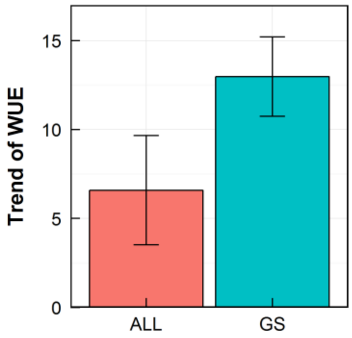

Figure R1 | Differences in observed trend of WUE at the Tumbarumba site (AU-Tum) estimated from all year data (ALL) and from growing-season data only (GS), which is defined as rain-free periods (by excluding rain days and the day following rain days) of summer months (Nov, Dec and Jan). The trend is in mg C mm^{-1} H_2O $year^{-1}$. The bars and error bars represent the mean and standard error from the 8 different estimates of GPP (see Supplementary Section 3 for more information about the data).

In the revised manuscript, we have deleted this statement in line 214 as suggested by the reviewer. Instead, we compared our trend estimate and attribution with results from LSMs by Huang et al., (2015)[14].

A8. I am well aware of the considerable uncertainties in the observations and I would not expect or ask for any perfect match but I am not sure the validation can be considered satisfactory to the point Eq. (1) can be used for all the subsequent discussion. In this article, validation is more important than in other contexts (e.g., the land surface models of Fig. S6) because the model is an analytical derivation and does not have any mechanistic structure on it. I would urge the authors to give more compelling motivations why the

reader should trust the results despite all these issues and how/why their results differ from previous estimates of trends in WUE and GPP.

Response: The updated validation shows that modelled trend of WUE agrees much better with the observed than the previous validation, as stated in our replies to previous comments.

In this study we further compared the magnitude, trends and global pattern of estimated WUE using equation (1) against other independent estimates including 6 land surface models and up-scaled global flux measurements (i.e. MTE results). All comparisons support that the WUE model captures satisfactorily the global annual WUE and its trend.

A9. Are the trends in GPP and WUE expected to be less sensitive to the assumptions in Eq. (1) then the magnitude themselves? Are these trends robust to the scatter of Fig. 1a. Maybe yes, but this argument needs to be explicit in the paper. Please also note that Eq. (1) is a simplification of our current understanding of WUE, while the unexplained variability could be in fact the most important aspect of changes in WUE, the one we still not fully understand and that may provide a future picture quite different from what we expect. I think this aspect needs a different emphasis in the article.

Response: Further analysis supports the reviewer's expectation that trends in estimated GPP and WUE is much less sensitive than the assumptions of equation (1) and input data than the magnitude themselves.

Some of the discrepancy between the estimated mean global WUE and those from LSMs and MET results from the uncertainty of input data, particularly vapour pressure deficit. For example, the mean annual WUE from 1982 to 2011 by our model varies from 1.64±0.02 for CRU-NCEP, 2.50±0.02 for PGF, and 2.09±0.03 g C mm^{-1} H_2O for WATCH 1982 to 2011. Although noticeable differences in magnitude of estimated mean global WUE from different datasets can be found, trends in global WUE is very close to each other and close to estimates from LSM and MTE methods and will not affect the conclusion of this study.

We have provided further discussion on the influences of uncertainties of the input dataset on the estimated WUE and GPP in the supplementary materials (Section 6.2).

Specific comments

A10. Line 21. I would use a "may have consequences" since there is still some debate on the effect of elevated CO2 in affecting runoff and streamflow (e.g., Huntington 2008).

Response: Agreed. Changes are made in the revised manuscript accordingly and the reference is cited to support this point (see lines 20-21).

A11. Line 35. Beyond soil, I would also suggest to mention forest demography, nutrients, and atmospheric feedbacks.

Response: Agreed. Changes are made in the revised manuscript accordingly (see lines 36-37).

A12. Line 40-42. This sentence is probably too optimistic, I concur that estimates of E from precipitation and streamflow, if the two above variables are reliably measured, can be quite accurate in the long-term (E of multiple years). However, at the annual scale it is likely not the case because changes in storage in the basin (groundwater, soil, eventually snow) can affect the computed E and E trends. Differences among products in Fig. S2 are supporting my concern.

Response: We thank the reviewer for this comment. We have revised this statement as "ecosystem water use, i.e. E, can be better constrained with widely available hydrological observations (including precipitation and streamflow) at catchment, regional and global scales, where direct measurements of E are not available" Please refer to lines 43-45 in the revised manuscript.

A13. Line 88-89. I guess there is a bit of circular reasoning, the spatial patterns of the computed GPP should naturally follow the one of the products used to compute Eq. (1), which includes MTE and land surface model simulations. Therefore, the agreement between GPP spatial details should not be used as a mean of validation (Fig. S7).

Response: This might be the case for the GPP estimated from the E_{MTE} dataset but not for GPP estimates from other six *E* datasets, since we did not use E from land surface models to estimate global GPP. Representation of WUE in most global land models is much more complex than our eqn(1), and the fact our predicted global pattern of GPP agrees well with GPP estimates using MTE method and from other LSM modelling (see Figure S5 in the revised manuscript) supports the validity of eqn(1). Therefore, it is not a circular reasoning.

A14. Line 130, 131, 134 and 135. A minor detail, I found a bit confusing that the percentage of significant trends is not given on the total but on the subset of positive or negative trends.

Response: Because of the positive and negative contributions by different factors to the overall positive trend of GPP and WUE, it is not meaningful to have only negative or positive percentage.

A15. Line 213-214. I believe this sentence is wrong, Keenan et al. 2013 found a much stronger trend in WUE rather than proportionality with Ca, actually this is the overall point of their article and the reason why it was published in Nature.

Response: Thank you for bringing this to our attention. We found a much smaller trend in WUE than that by Keenan et al. (2013) due to mainly different regions studied and different period of data used (also the response to A7 and Figure R1). In the revised manuscript, we have deleted this statement. Instead, we compared our estimation of trend and attributions with results from LSMs by Huang et al., (2015)[14] (see lines 233-236).

A16. Line 230-232. This sentence is repetitive with what you stated previously.

Response: We thank the reviewer. In the revised manuscript, we rewrite the last sentence as "Overall, this study provides a promising way to couple the carbon and water cycles via independent estimate of ecosystem WUE and our findings suggest that the recent increase in global carbon uptake is not at the cost of using more water but due to using water more efficiently."

A17. Line 259-260. Is Ca used as spatial uniform in the entire Earth? Or geographical differences are preserved?

Response: We apologize for this oversight. The C_a data used is annual mean values observed at the Mauna Loa site[15] and is spatially uniform, but vary from year to year. More details about the C_a data are provided in the revised manuscript. Please refer to lines 281-282.

A18. Line 287-290. The explanation on the partition of the different contributions remains unclear to me. Only reading the Supp. Material I could understand what the authors did.

Response: We apologize for this oversight. More details on the partition of the total trends to different factors are provided in the methods in the revised manuscript (see lines 311-319).

A19. Figure (4). In the caption, it should be "leaf area index (e)"

Response: Thank you for pointing out this typo. We corrected the "leaf area index (d)" to "leaf area index (e)".

References

Frank, D. C. et al. (2015), Water-use efficiency and transpiration across European forests during the Anthropocene, Nat. Clim. Chang., 5, 579-583, doi:10.1038/nclimate2614.

Xue, B.-L., Q. Guo, A. Otto, J. Xiao, S. Tao, and L. Li (2015), Global patterns, trends, and drivers of water use efficiency from 2000 to 2013, Ecosphere, 6, 174,

Keenan, T. F., D. Y. Hollinger, G. Bohrer, D. Dragoni, J. W. Munger, H. P. Schmid, and A. D. Richardson (2013), Increase in forest water-use efficiency as atmospheric carbon dioxide concentrations rise, Nature, 499, 324-7, doi:10.1038/nature12291.

Saurer, M., R. T. W. Siegwolf, and F. H. Schweingruber (2004), Carbon isotope discrimination indicates improving water-use efficiency of trees in northern Eurasia over the last 100 years, Glob. Chang. Biol., 10, 2109-2120, doi:10.1111/j.1365-2486.2004.00869.x.

Schlesinger, W. H., and S. Jasechko (2014), Transpiration in the global water cycle, Agric. For. Meteorol., 189-190, 115-117.

Medlyn, B., and M. De Kauwe (2013), Biogeochemistry: Carbon dioxide and water use in forests, Nature, 499, 287-289, doi:10.1038/nature12411.

Huntington, T.G., 2008. CO2-induced suppression of transpiration can not explain increasing runoff. Hydrol. Process. 22, 311-314.

Responses to reviewer #2:

Review of Recent increases in terrestrial carbon uptake at little cost to the water cycle for Nature Communications.

The paper describes a model of optimal stomatal conductance used to estimate ecosystem water use efficiency (WUE). The stomatal conductance equations and parameters have been applied lately to land surface models, but this is a novel application in that several observation datasets are used as inputs in the model to predict global WUE. Based on seven datasets of global evaporation (E), an ensemble of global GPP is generated. This is used to show increases in GPP since 1982, due to increases in WUE and leaf area index (L).

The strength of this paper is the application of the optimality model, which has recently been used and suggested as an improvement over previous empirical stomatal conductance models. Another strength is the large number of datasets used to give an uncertainty range, which is key to include for this difficult to estimate value. This is a potentially very useful dataset and model of WUE.

Although I think the paper could make a valuable contribution, I can see a few weaknesses, which might make the paper unsuitable for Nature Communications in its current form. I will explain further here and provide suggestions when possible.

Major Comments

B1. Pertaining to ability of the WEC model to reproduce observed trends in WUE.

I am not fully convinced that the model is able to capture the observed trends in WUE at Fluxnet sites. The observed mean trend is 20.4±7.4 mg C mm-1 H2O year-1, and I disagree that this is "consistent" with the model-predicted trend of 37.0±2.7 mg C mm-1 H2O year-1 (Lines 67-70), as the latter is nearly twice as high as the observations.

One problem is the significance of the trends shown in Figure 1b. It's very difficult to tell how much to trust the trend lines given the large variation in individual sites. So can we even say with confidence that WUE at the sites is increasing? And the same for the WEC model results? For the sites shown in Figure S5, it would be useful to include the slope of the trend lines and whether they are significantly different from zero.

Also, in the SI, it is stated that WUE increased by 35.9±23.0 mg C mm-1 H2O year-1 in the model and by 13.6±20.2 mg C mm-1 H2O year-1 in the observations (Lines 150-154). Why are these numbers different from those provided in the main text?

Response: Thank you for the constructive comment. Previous validation of the trend was not very good primarily due to limited number of sites with high quality and long term (>6 years) observations. The previous validation only used data up to 2011 and observations of most of flux stations begun after 2000. We updated the validation using the latest global flux dataset, i.e. FLUXNET2015, of which data availability is up to 2014 at some stations.

With FLUXNET2015 dataset, the agreement between estimated and observed WUE trends at 11 flux stations is significantly improved (see Figure 1b). At the 11 stations with long-term

annual data, mean estimated trend is about 13.7±11.1 mg C mm^{-1} H_2O $year^{-1}$ and observed trend is about 12.6±11.4 mg C mm^{-1} H_2O $year^{-1}$. The linear correlation coefficient (*r*) between observed and estimated trends in annual WUE is about 0.74, mean error (ME) of 1.12 mg C mm^{-1} H_2O $year^{-1}$ and relative error (RE) of 8.9%.

Please refer to Section 3 of the supplementary materials for more details about the new validation.

B2. I think there are important implications of the WEC model overpredicting increase in WUE over the study period. This means the predicted increases in GPP are possibly to high. A useful paper to compare to is Large divergence of satellite and Earth system model estimates of global terrestrial CO2 fertilization by WK Smith et al. They found many more regions of decreased NPP than found for GPP in this study (obviously you cannot make a direct comparison between NPP and GPP, but it's likely the large-scale patterns of change should be similar). Another check could come from the GPP based on upscaled Fluxnet data – are trends in GPP similar?

Response: Many thanks for the suggestion. We do not have good independent data to verify the trends in GPP and WUE, as currently available GPP and WUE estimates at global scale come from models with considerable uncertainties (see ref. 16, ref. 17, ref. 18, ref. 19, ref. 20, ref. 21, ref. 22). Across the 11 eddy flux sites, our model slightly over predicted the trend in WUE (~9%, see Figure 1b) compared with observed WUE trends, which is well within the uncertainty ranges in observations.

We agree with reviewer that trends of GPP and NPP are not comparable because of the additional process (autotrophic respiration is involved and varies with external drivers quite differently from GPP). Nonetheless, the global pattern of changes in NPP derived from satellite data (i.e. Figure 2c in Smith et al., (2015)[23]) is similar to that of our GPP changes (Figure 3a in our study). We both predict a negative trends in GPP and NPP in most of the arid regions such as the northeast China and Mongolia, the inland of Australia, Sahel region, southern part of the South American, and part of the western United States; and a positive trends in GPP and NPP in most of the boreal and tropical forests regions. At global scale, they reported that NPP increased over 1982-2011 based on the satellite data, which is consistent with the overall positive trend in GPP we derived in this study over the same period. Therefore results of our study are quite consistent with that by Smith et al., (2015)[23].

We haven't attempted to compare the GPP trend with the MTE dataset as it has been reported to underestimate the global GPP changes, because CO_2 fertilization effect has not been considered in the MTE GPP product (see ref. 14, ref. 24, ref. 25).

B3. Another question I have is: How sensitive is the WEC model to the leaf area index, L? The assumption behind Eq. 5 is that ET dominates for high LAI canopies and decreases quickly as L decreases. Is there any data to back up this statement, or is it possible that the effect of L saturates quickly, so for high L it doesn't really matter (once the ground is covered by a tree, the evaporation from soils will not easily get into the atmosphere whether the tree has L of 4 or 7, for example). To me this is important, because the increases in L over the study period mean that increases in D are overwhelmed, so the WUE increases. However, this seems to be

in contrast to the findings of Smith et al 2015, where NPP sensitivity to VPD was shown to increase over the same time period (1982-2011).

Response: The control of LAI on the partitioning between transpiration and soil evaporation has been studied before (see Schulze et al. 1994[2], Kelliher, et al.[3] and Wang et al., 2014[4]). Experimental studies suggest that soil evaporation is negligible when canopy is closed (L>3), but it is also true that the canopy interception ratio (evaporation from wet canopy to E) is larger for higher L (see ref. 26). Therefore, it does not necessarily mean that the fraction of water use for production (i.e. Et/E) follows the exponential relationship of the partitioning between transpiration and soil evaporation.

In this study, we focused on the trend in GPP and WUE. Globally we find that positive effect of increasing C_a is larger than the negative effect of VPD on WUE trend, and therefore GPP, which differs from the effects on NPP. However the trend of WUE and GPP can be different from NPP because of additional process (autotrophic respiration) is involved. Also see my reply to B2.

Minor comments

B4. 1. Lines 80-82: It would be useful to summarize in 1-2 sentences the broad spatial patterns of WUE. Also, there is a large divergence in global patterns of WUE from the land surface models shown in Figure S6 (especially in high latitudes).

Response: Thanks. As suggested, a more detailed summary about the estimated global WUE has been made in the revised manuscript. Please refer to lines 87-92.

B5. 2. Lines 140-142: Are panels c and d switched in Figure 3? In the figure, there is a large decrease in GPP in central Africa, but the text states the opposite.

Response: Thank you for pointing out this error. The order of the panels of Figure 3 is right, the error is in the text. The right statement should be "Central Africa experienced a decrease in GPP (Figure 3a) but an increase in WUE (Figure 3b), which is associated with a significant declining E trend in this region outweighing the increase in WUE (Figure 4a and 4b)." Please refer to lines 150-152.

B6. 3. Lines 183-184: This is the first time the parameter g1 is mentioned in the main text, I think it should be explained a bit so readers do not necessarily have to go through the SI find out what it is.

Response: The parameter g_1 firstly appeared in line 47, where a short note about this parameter is given as well. With regarding to the importance of this parameter, one sentence is added in the revised manuscript where the analytical model is introduced briefly. Please refer to lines 52-55.

B7. 4. Lines 207-209: This suggests that tropical forest GPP is very sensitive to Ca. A recent study also found large increases in tropical tree WUE, but no concurrent increases in tree

growth (van der Sleen et al. 2014). However as both of these studies suggest large increases WUE, the relationship between these results would be worth including in the discussion.

Response: Thank you for bringing this to our attention; we have cited this reference van der Sleen et al. (2014)[27] in the revised manuscript to support our conclusion.

B8. 4. Lines 211-212: Can the observed effect on NPP from FACE be explicitly stated?

Response: The ref. 28 reported that NPP increase in the forested FACE experiments are about 23% to an increase of CO_2 about 45% (from 380ppm to 550ppm). That is to say NPP increase from FACE experiments is about 5% per 10% increase in C_a. As suggest, we have stated the FACE experiment effects in the revised manuscript (see lines 230-233).

B9. 5. The assumption in moving from Eq. 1 to Eq. 3 (in the SI) is valid for the growing season only – are all model equations only calculated during the growing season as well?

Response: We apologize for this oversight. More details about how ecosystem annual WUE is estimated is provided in the supplementary materials. Please refer to lines 67-74 in the Section 1 of the supplementary materials for more details.

B10. 6. There is a similar paper which was recently published in ESD by Dekker et al. Using a different approach to calculating WUE, the authors found larger changes in fractional WUE over the 20th century than suggested by the optimality approach used in this study. It would be interesting to know how these results fit in the context of the sensitivities found in that study, which also used tree ring data.

Response: According to our results, we predict that global WUE has increased at a rate of 14% per 10% increase in C_a over the period of 1982-2011, which suggest a higher (~1.4) sensitivity than that derived from stomatal optimization theories. It is consistent with Dekker et al (2016)[25] that the sensitivity of ecosystem WUE to C_a is about 1.51±0.57 obtained from eddy flux and long-term tree ring records. In the revised manuscript, findings by Dekker et al (2016) is cited to support our conclusions (see lines 233-237 in the revised manuscript).

References:

van der Sleen et al 2014: No growth stimulation of tropical trees by 150 years of CO2 fertilization but water-use efficiency increased, Nature Geoscience, DOI: 10.1038/NGEO2313

Smith et al 2015: Large divergence of satellite and Earth system model estimates of global terrestrial CO2 fertilization, Nature Climate Change, DOI: 10.1038/NCLIMATE2879

Dekker et al 2016: Spatial and temporal variations in plant water-use efficiency inferred from tree-ring, eddy covariance and atmospheric observations, Earth System Dynamics, DOI: 10.5194/esd-7-1-2016.

Responses to reviewer #3:

C1. The manuscript is original and presents an interesting new approach. WUE as an emergent ecosystem property is getting a lot of exposure lately (see also Reichstein et al 2014 PNAS, which, as a side note, should be referenced in this manuscript), and this manuscript provides an elegant methodology to use WUE as a predictive variables.

There is an argument for and against the approach taken here. There could be something intrinsic and physically driven in WUE, and if that is the case the approach presented here could be extremely useful. However it is also possible that WUE is the emergent result of multiple plant and meteorological processes that couple the atmosphere to canopy transpiration and carbon uptake. If that is the case, any model that is made to fit the current observed WUE is essentially over-fitted because it ignores all the independent processes whose dynamics could change in complex ways as a result of climate change.

Response: Thank you for bringing this to our attention. Whether WUE can be considered as an emerging property also depends on the time scale and objective of the study, which is not the main focus of this work. We stated the objectives and underlying assumptions of model development explicitly in the revised manuscript (see Section 1.1 and 1.2 of Supplementary materials). The work by Reichstein et al (2014)[29] is very interesting, and is now cited in the revised manuscript to support the importance of this study and conclusions.

C2. In the introduction the authors present the climate change and carbon fertilization responses of global vegetation (L17 and L22) as these are well known and accepted and reference 3 papers, where, in fact, there is a large debate and many papers each year provide the results of many long-term observation and synthesis papers and large scale FACE experiments with no uniform conclusion. You should revise this section, to emphasize the lack of consensus, and then your next sentence will actually make sense "Therefore, quantification of the response of the water and carbon cycles − which are closely coupled as both diffuse through leaf stomata − to global warming and rising Ca is very complex, and much needed."

[Notice that I edited that sentence somewhat, it was badly phrased and had some grammatical errors].

Response: We thank the reviewer for the constructive suggestions. We rewrote the second half of the first paragraph as suggested. Please refer to lines 21-28 in the revised manuscript.

C3. One key problem I have with this approach is the assumption that (L40-42) "On the contrary, ecosystem water use, i.e. E, which can be estimated from catchment hydrological observations (including precipitation and streamflow), is quite accurate." I totally do not agree that E estimated from catchment hydrological observations is "quite accurate". I have seen many, and in my experience they are all far from accurate. In fact, the runoff and soil moisture are the least observed variabwhreles from remote sensing, and there are very few satellite product and very coarse datasets for these, as compared for example to vegetation greenness

and GPP that is derived from it. It is not obvious that a method based on estimated E values has an upfront advantage relative to other methods that are based on vegetation greenness.

Response: Thank you for the insightful comment. Given the eddy flux towers only have a footprint of 1 km^2 or so, and most of those towers are located on flat terrain, many water catchments are much larger, and can be as large as the order of 10^4 km^2. Globally hydrological measurements including runoff and precipitation are widely available. What we argue here is that those long-term hydrological measurements should be helpful in constraining the estimates of GPP at a spatial scale larger than the footprints of flux towers if a strong connection between water and carbon fluxes can be found, as did in this study.

We agree that "is quite accurate" is an overstatement and has been revised as "ecosystem water use, i.e. *E*, can be better constrained with widely available hydrological observations (including precipitation and streamflow) at catchment, regional and global scales, where direct measurements of E are not available". Please refer to lines 38-45 in the revised manuscript.

C4. And an editorial side comment to this sentence (L40-42), it is not clear what this is "on the contrary" for? Contrary to estimates of ecosystem GPP? Contrary to global scale estimates of WUE?

Response: Thank you for pointing out this oversight. With regard to "on the contrary", we meant that estimates of ecosystem E at large spatial scale (>1km^2) can be derived from direct hydrological observations but ecosystem GPP at large and long-tern scales is unobservable and estimation of ecosystem GPP relies heavily on land surface models (also see our response to C2). We have revised the sentence to make it clear. Please refer to lines 38-45 in the revised manuscript.

C5. The validity of the analytical WUE model – I am impressed by the r^2 of 0.68 of the annual WUE, tough Nash-Sutcliffe model efficiency of 0.34 is not so great. I would not predict an r^2 as high even for the comparison between the mean 0.5x0.5 deg E from the global data sources you used and direct observations of evapotranspiration from the flux towers (this will be a nice exercise to check).

Response: Thanks for the comment. The global E data products we used have been evaluated comprehensively before, including against flux tower data (see original references of E_{MTE}[24, 30], E_{GLEAM}[31, 32], E_{CSIRO}[33], $E_{WB\text{-}MTE}$[34], E_{MERRAa}[35], E_{MERRAs}[36], and E_{ERA}[37]).

C6. The high r^2 is particularly surprising given that the method used for its calculation of WUE is extremely simplistic. It neglects canopy structure (k is constant), reduces plant heterogeneity to a single variable g1 and driven by the SYNMAP PFTs. In my experience, most flux sites have their footprints over vegetation composition very different than the generalization of SYNNMAP for that are, and I know that WUE is variable between species of the same PFT in the same site. Your formulation also neglects stomata dynamics which determine when and at what D water is evaporated and carbon is assimilated, with big differences between days of wet/dry soil. A change in stomata opening toward the morning

(a common effect of dry soil) can reduce WUE as more carbon uptake is done earlier in the day when radiation is limiting. Finally it is driven by highly inaccurate estimates of E. I am also confused as to what D you are using? The mean monthly D? There are strong diurnal fluctuations in D and the values that are relevant to WUE are those present during the time the plants are actively transpiring.

Response: We agree with the referee fully that parameterization of vegetation properties is one of significant uncertainties in estimating global ecosystem WUE. This is also true for other carbon related quantities[29, 38]. In this study, two other different global land cover maps are collected to test the influences of different land cover on the estimated WUE. The two land cover maps are MODIS land cover map and the vegetation cover map used for the CABLE land surface model (see ref. 39). Results show that there is no significant difference in estimated global WUE patterns, magnitude and trends globally from three different land cover maps, but there is significant difference in some limited area regionally. Finally, the global synergetic land cover product, i.e. SYNMAP, is adopted as its overall advantages for carbon cycle modelling comparing with other land cover products (see ref. 38). An extended discussion on this is provide in the supplementary materials (see Section 6.1).

Our model is developed for annual time scale and is therefore not applicable to sub-diurnal time scale. We have now stated this very clearly in the revised manuscript and a short discussion on this potential limitation has been provided in the revised manuscript (see lines 200-208)

C7. A small "cheat" is using the observed D for the site comparison. It makes sense, however, it hinders the validation of the global approach. If you had used the 0.5x0.5 deg D from the global datasets, how much would it reduce the accuracy of the WUE estimation?

Another cheat is throwing all the sites where WUE is high. I accept removing sites with observation problems, such as the sites with energy closure of less than 75%. But why kick out all the sites with high WUE? This will directly affect the bias of your model and make it look better than it is.

Given all that, I want to see more to be convinced that your formulation actually predicts the correct WUE for the correct reasons.

Response: We also conducted the validation using *D* identified from global dataset, with which validation degraded slightly in bias about 3% and in linear correlation about 0.06 compared with that using site observed D as shown in Figure 1a. We used site observed *D* for validation as spatial resolution of global datasets (0.5 degree) is much larger than the footprint of flux towers and *D* is one of most commonly meteorological variables that provided with the flux observation.

We updated the validation using the latest global flux dataset, i.e. FLUXNET2015. Detailed criteria for selecting data are provided in the Section 3 of the supplementary materials. We have not tried to exclude high observed annual WUE values (> 6 gC/mmH_2O) in current validation as none of the observed annual WUE is larger than 6 gC/mmH_2O in the current dataset. Previously, annual WUE larger than 6 gC/mmH_2O as excluded, as those high values were expected to result from observational errors. It is not needed in the updated FLUX2015 dataset.

C8. You write in the appendix that WUE is calculated monthly, but you only show the annual means. How does the Monthly fair?

Response: This study focused on the changes in annual ecosystem WUE, which is aggregated from monthly estimates of different components of equation (1) with a few assumptions. Monthly WUE is not the focus of this study and thus is not analysed and studied. We now provide more details in the last paragraph of supplementary section 1.1.

C9. Most importantly, how does your model do in predicting the change in WUE? In sites where there is a long-term trend in WUE, did your model find this trend? Over all sites you report a predicted increase of WUE 37+-2.7 per year, and claim it fits an observed 20+-7.4 per year. I do not agree. Your estimate is far out of the range, and almost twice as large as the observation. I conclude that your model has a large bias in the trend of WUE, much larger than its bias in the mean WUE. There could be many reasons for this. I would suspect missing mechanisms. Models could be over parameterized and represent the means during a certain time very well but will fail predicting long term trends. Specifically, can you translate figure S5 to a scatterplot of the observed vs predicted slopes of WUE change over time? In at least 4 of the sites you predict the opposite trend to the observed. I am curious as to how it will look overall.

Response: We have redone the validation using the latest global flux data (i.e. FLUXNET2015), and the agreement between the predicted and observed trends agree much better.

We agree that previous trend validation is not very good, largely due to the limited number of sites with high quality and long-term observations. We updated the validation work in the revised manuscript using the latest global flux dataset, i.e. FLUXNET2015, with data available up to 2014 at some stations. Now, we use 243 station-years data to validate the model and long-term (greater than 6 years) annual data of 11 stations are used to validate the trends in annual WUE. The spatial validation is as good as in the previous manuscript, however, the validation of trend is much better in the revised analysis than in the previous one (see Figure 1b). At the 11 stations with long-term annual data, mean estimated trend is about 13.7±11.1 mg C mm^{-1} H_2O $year^{-1}$ and observed trend is about 12.6±11.4 mg C mm^{-1} H_2O $year^{-1}$. The linear correlation coefficient (*r*) between observed and estimated trends in annual WUE is about 0.74, with Nash-Sutcliffe model efficiency (NSE[11]) of 0.50, root mean squared error (RMSE) of 25.6 mg C mm^{-1} H_2O $year^{-1}$, mean error (ME) of 1.12 mg C mm^{-1} H_2O $year^{-1}$ and relative error (RE) of 8.9%. We refer the reviewer to Section 3 of the supplementary materials for more details about the new validation.

As suggested, we replotted the estimated and observed trends at different stations using scatter plot as shown in Figure 1b in the revised manuscript.

C10. Similarly, I want to see a validation of E. All flux sites directly measure E, but you use E from global datasets. Are these anything close to the flux sites? Do they capture the long term trends in E as observed in the flux sites?

Response: We agree that trend in E is also of crucial importance to understand changes in the coupled water and carbon cycles. Validation of global E datasets against flux measurements has been published before (see original references of E_{MTE}[24, 30], E_{GLEAM}[31, 32], E_{CSIRO}[33], E_{WB-MTE}[34], E_{MERRAa}[35], E_{MERRAs}[36], and E_{ERA}[37]).

We found that global trend in GPP is much larger than that of E, which indicates that trend in GPP is largely contributed by WUE (~90%) rather than E. There is a general agreement that trend in global E is very small comparing with mean annual E[30, 33, 40]. Therefore, we used global E datasets directly and assumed that these 7 diagnostic and reanalysis E datasets can provide us the state of art of understanding about how terrestrial evaporation is changed over the past three decades.

C11. A related question – how did you choose the sites?

I know many more sites in the Ameriflux dataset that have more than 7 years of data. But they are not used for the trend evaluation. Why?

Response: The criteria for selecting flux station data are provided in Section 3 of the supplementary materials. Basically, we considered the length of available records, quality of the data (e.g. complete annual records, high quality observed data), and available data for energy closure corrections. Given that the purpose of our study is to examine annual trends in WUE, flux stations that are not meeting these criteria are excluded from the study.

C12. It will be reasonable to add in one supplement a cite table listing all the sites you used, their lat-long, the period of data they have (only years with high data quality that were actually used in the analysis), what dataset you got them from (Ameriflux, Fluxnet, ChinaFlux…), what was the vegetation PFT you assigned to it (g1). Without this information it'll be impossible to repeat the work you did.

Response: As suggested, a table of the sites used for validation is provided in the supplementary information (i.e. Table S2).

C13. Finally, the manuscript is written rather poorly. I am not a native English speaker and tend not to complain about language, but even I got disturbed by the many grammatical error and awkward sentencing throughout.

I was editing the grammar but gave up after 2 pages.

Here are some corrections that I suggest:

L9 does not commensurate

L11 per unit of water used (i.e. water use efficiency).

L12 canopy leaf area index, and negatively

L13 suggest that rising atmospheric

L16 Global climate change, caused mainly by

L21 consequences to the hydrological cycle

L24-25 Understanding how the coupled water and carbon cycles will change under future climate and rising

L28-31 Ecosystem WUE, defined as GPP per unit ecosystem water loss via E, is one of the most important functional properties of ecosystems that drive terrestrial carbon and water exchanges with the atmosphere4,5,15. Analytical models based on the optimization theory can explain leaf WUE, defined as leaf photosynthetic carbon uptake per unit water loss via transpiration, variations with plant function types and climate16,17. However, understanding of ecosystem WUE is still very limited5,15,18,19.

L36-30 It is really hard to figure out what you are trying to say here. What is "prior"?

L50 studies on WUE (e.g. 5,15,19) [ref numbers in superscript]

L52 not requiring prior estimates

Response: We appreciate all the language edits and have made the suggested changes in the revised manuscript. Meanwhile, the revised manuscript has been edited by native English speakers before submission.

References

[1]Schlesinger, W. H., and Jasechko, S. Transpiration in the global water cycle, *Agr. Forest Meteorol.* **189 – 190**, 115-117 (2014).

[2]Schulze, E. D., Kelliher, F. M., Korner, C., Jon, L., and Leuning, R. Relationships Among Maximum Stomatal Conductance, Ecosystem Surface Conductance, Carbon Assimilation Rate, and Plant Nitrogen Nutrition: A Global Ecology Scaling Exercise, *Annual Review of Ecology and Systematics* **25**, 629-660 (1994).

[3]Kelliher, F. M., Leuning, R., Raupach, M. R., and Schulze, E. D. Maximum conductances for evaporation from global vegetation types, *Agr. Forest Meteorol.* **73**, 1-16 (1995).

[4]Wang, L., Good, S. P., and Caylor, K. K. Global synthesis of vegetation control on evapotranspiration partitioning, *Geophys. Res. Lett.* **41**, 6753-6757 (2014).

[5]Medlyn, B. E., Duursma, R. A., Eamus, D., Ellsworth, D. S., Prentice, I. C., and Barton, C. V. M. *et al.* Reconciling the optimal and empirical approaches to modelling stomatal conductance, *Global Change Biol.* **17**, 2134-2144 (2011).

[6]Lin, Y., Medlyn, B. E., Duursma, R. A., Prentice, I. C., Wang, H., and Baig, S. *et al.* Optimal stomatal behaviour around the world, *Nature Clim. Change* **5**, 459-464 (2015).

[7]De Kauwe, M. G., Kala, J., Lin, Y. S., Pitman, A. J., Medlyn, B. E., and Duursma, R. A. *et al.* A test of an optimal stomatal conductance scheme within the CABLE land surface model, *Geosci. Model Dev.* **8**, 431--452 (2015).

[8]Wong, S. C., Cowan, I. R., and Farquhar, G. D. Stomatal conductance correlates with photosynthetic capacity, *Nature* **282**, 424-426 (1979).

[9]Wong, S. C., and Dunin, F. X. Photosynthesis and Transpiration of Trees in a Eucalypt Forest Stand: CO_2, Light and Humidity Responses, *Funct. Plant Biol.* **14**, 619-632 (1987).

[10]Barton, C. V. M., Duursma, R. A., Medlyn, B. E., Ellsworth, D. S., Eamus, D., and Tissue, D. T. *et al.* Effects of elevated atmospheric [CO_2] on instantaneous transpiration efficiency at leaf and canopy scales in Eucalyptus saligna, *Global Change Biol.* **18**, 585 – 595 (2012).

[11]Nash, J. E., and Sutcliffe, J. V. River flow forecasting through conceptual models part I — A discussion of principles, *J. Hydrol.* **10**, 282-290 (1970).

[12]Zhu, Z., Piao, S., Myneni, R. B., Huang, M., Zeng, Z., and Canadell, J. G. *et al.* Greening of the Earth and its drivers, *Nature Clim. Change* **in press** (2016).

[13]Keenan, T. F., Hollinger, D. Y., Bohrer, G., Dragoni, D., Munger, J. W., and Schmid, H. P. *et al.* Increase in forest water-use efficiency as atmospheric carbon dioxide concentrations rise, *Nature* **499**, 324 – 327 (2013).

[14]Huang, M., Piao, S., Sun, Y., Ciais, P., Cheng, L., and Mao, J. *et al.* Change in terrestrial ecosystem water-use efficiency over the last three decades, *Global Change Biol.* **21**, 2366 – 2378 (2015).

[15]Keeling, C. D., Bacastow, R. B., Bainbridge, A. E., Ekdahl, C. A., Guenther, P. R., and Waterman, L. S. *et al.* Atmospheric carbon dioxide variations at Mauna Loa Observatory, Hawaii, *Tellus* **28**, 538-551 (1976).

[16]Le Quere, C., Raupach, M. R., Canadell, J. G., and Marland Et Al., G. Trends in the sources and sinks of carbon dioxide, *Nat. Geosci.* **2**, 831-836 (2009).

[17]Field, C. B., Jackson, R. B., and Mooney, H. A. Stomatal responses to increased CO_2: implications from the plant to the global scale, *Plant, Cell & Environment* **18**, 1214-1225 (1995).

[18]Gregory, J. M., Jones, C. D., Cadule, P., and Friedlingstein, P. Quantifying Carbon Cycle Feedbacks, *J. Climate* **22**, 5232-5250 (2009).

[19]Medlyn, B. E., Zaehle, S., De Kauwe, M. G., Walker, A. P., Dietze, M. C., and Hanson, P. J. *et al.* Using ecosystem experiments to improve vegetation models, *Nature Clim. Change* **5**, 528-534 (2015).
[20]Gedney, N., Cox, P. M., Betts, R. A., Boucher, O., Huntingford, C., and Stott, P. A. Detection of a direct carbon dioxide effect in continental river runoff records, *Nature* **439**, 835-838 (2006).
[21]Piao, S., Friedlingstein, P., Ciais, P., de Noblet-Ducoudré, N., Labat, D., and Zaehle, S. Changes in climate and land use have a larger direct impact than rising CO_2 on global river runoff trends, *P. Natl. Acad. Sci. Usa.* **104**, 15242 -15247 (2007).
[22]Huntington, T. G. CO_2-induced suppression of transpiration cannot explain increasing runoff, *Hydrol. Process.* **22**, 311-314 (2008).
[23]Smith, W. K., Reed, S. C., Cleveland, C. C., Ballantyne, A. P., Anderegg, W. R. L., and Wieder, W. R. *et al.* Large divergence of satellite and Earth system model estimates of global terrestrial CO2 fertilization, *Nature Clim. Change* **6**, 306-310 (2016).
[24]Jung, M., Reichstein, M., Margolis, H. A., Cescatti, A., Richardson, A. D., and Arain, M. A. *et al.* Global patterns of land-atmosphere fluxes of carbon dioxide, latent heat, and sensible heat derived from eddy covariance, satellite, and meteorological observations, *J. Geophys. Res.* **116**, G00J07 (2011).
[25]Dekker, S. C., Groenendijk, M., Booth, B. B. B., Huntingford, C., and Cox, P. M. Spatial and temporal variations in plant water-use efficiency inferred from tree-ring, eddy covariance and atmospheric observations, *Earth Syst. Dynam.* **7**, 525--533 (2016).
[26]Miralles, D. G., Gash, J. H., Holmes, T. R. H., de Jeu, R. A. M., and Dolman, A. J. Global canopy interception from satellite observations, *Journal of Geophysical Research: Atmospheres* **115**, D16122 (2010).
[27]van der Sleen, P., Groenendijk, P., Vlam, M., Anten, N. P. R., Boom, A., and Bongers, F. *et al.* No growth stimulation of tropical trees by 150 years of CO_2 fertilization but water-use efficiency increased, *Nat. Geosci.* **8**, 24-28 (2015).
[28]Norby, R. J., DeLucia, E. H., Gielen, B., Calfapietra, C., Giardina, C. P., and King, J. S. *et al.* Forest response to elevated CO_2 is conserved across a broad range of productivity, *P. Natl. Acad. Sci. Usa.* **102**, 18052 -18056 (2005).
[29]Reichstein, M., Bahn, M., Mahecha, M. D., Kattge, J., and Baldocchi, D. D. Linking plant and ecosystem functional biogeography, *Proceedings of the National Academy of Sciences* **111**, 13697 -13702 (2014).
[30]Jung, M., Reichstein, M., Ciais, P., Seneviratne, S. I., Sheffield, J., and Goulden, M. L. *et al.* Recent decline in the global land evapotranspiration trend due to limited moisture supply, *Nature* **467**, 951-954 (2010).
[31]Miralles, D. G., De Jeu, R. A. M., Gash, J. H., Holmes, T. R. H., and Dolman, A. J. Magnitude and variability of land evaporation and its components at the global scale, *Hydrol. Earth Syst. Sc.* **15**, 967-981 (2011).
[32]Miralles, D. G. Global land-surface evaporation estimated from satellite-based observations, *Hydrol. Earth Syst. Sc.* **15**, 453 (2011).
[33]Zhang, Y., Peña-Arancibia, J. L., McVicar, T. R., Chiew, F. H. S., Vaze, J., and Liu, C. *et al.* Multi-decadal trends in global terrestrial evapotranspiration and its components, *Sci. Rep.-UK* **6**, 19124 (2016).
[34]Zeng, Z., Wang, T., Zhou, F., Ciais, P., Mao, J., and Shi, X. *et al.* A worldwide analysis of spatiotemporal changes in water balance-based evapotranspiration from 1982 to 2009, *Journal of Geophysical Research: Atmospheres* **119**, 1186-1202 (2014).
[35]Rienecker, M. M., Suarez, M. J., Gelaro, R., Todling, R., Bacmeister, J., and Liu, E. *et al.* MERRA: NASA's Modern-Era Retrospective Analysis for Research and Applications, *J. Climate* **24**, 3624-3648 (2011).

[36]Reichle, R. H., Koster, R. D., De Lannoy, G. J. M., Forman, B. A., Liu, Q., and Mahanama, S. P. P. *et al.* Assessment and Enhancement of MERRA Land Surface Hydrology Estimates, *J. Climate* **24**, 6322-6338 (2011).
[37]Dee, D. P., Uppala, S. M., Simmons, A. J., Berrisford, P., Poli, P., and Kobayashi, S. *et al.* The ERA-Interim reanalysis: configuration and performance of the data assimilation system, *Q. J. Roy. Meteor. Soc.* **137**, 553-597 (2011).
[38]Jung, M., Henkel, K., Herold, M., and Churkina, G. Exploiting synergies of global land cover products for carbon cycle modeling, *Remote Sens. Environ.* **101**, 534-553 (2006).
[39]Wang, Y. P., Kowalczyk, E., Leuning, R., Abramowitz, G., Raupach, M. R., and Pak, B. *et al.* Diagnosing errors in a land surface model (CABLE) in the time and frequency domains, *Journal of Geophysical Research: Biogeosciences* **116**, G01034 (2011).
[40]Miralles, D. G., van den Berg, M. J., Gash, J. H., Parinussa, R. M., de Jeu, R. A. M., and Beck, H. E. *et al.* El Niño – La Niña cycle and recent trends in continental evaporation, *Nature Clim. Change* **4**, 122-126 (2014).

Reviewers' Comments:

Reviewer #1 (Remarks to the Author)

The authors addressed satisfactorily most of the previous comments I made. A major revision in the manuscript is the use of a more accurate dataset (FLUXNET2015), which covers an extended period, for the validation of the analytical model (Eq. 1). The station selection and the way trends in annual WUE (Fig 1b) are compared is also different. This substitution of dataset improved the model validation and the confidence on the study. Of course, this is only related to the use of the dataset since ultimately the presented model and assumptions are the same. The unexplained variability remain close to 50% (Line 70 and Line 76), which I think needs a more explicit mention in the discussion.

I also see that trends in estimated GPP and WUE are much less sensitive to the assumptions of equation (1) and input data than the magnitude themselves (Supp, material section 6.2). I believe this is a point in favor of the study that should also be stated explicitly in the manuscript and not only implicitly in Figure 2a,b. I appreciated the discussion about the potential limitations of the analytical model and land surface models with regards to LAI (Line 240-243) but I would also suggest to state explicitly that a change in leaf area index cannot affect GPP directly in the analytical model. This is important and is not even discussed in the rebuttal, where only the ET dependence on LAI through input data is mentioned.

A potential explanation for the difference with the results of Keenan et al. 2013 is provided only in the rebuttal letter, and substantially the issue is omitted from the article but I conform to the authors' choice.

SPECIFIC COMMENTS

Line 40. I respectfully disagree that is "difficult" to identify key controls on change in WUE from land-surface models. These are mechanistic models, therefore their results should be traceable to some physical principle, process interaction or parameters. If this is not typically done, it is a different story.

Line 72-74. From the uncertainty bounds of trends in ecosystem WUE (close to 100% of the mean) provided in this version of the article, it emerges that site estimates of this quantity are very poorly constrained by current data. In a way, I am not surprised by this uncertainty, but I believe this is important and needs to be explicitly stated in the article.

Line 202-204. I respectfully disagree on the fact that nutrient availability or soil water changes necessarily act on temporal scales finer than the year. Actually, I would argue that the presented approach neglect fine temporal scale and also long-term adaptations (e.g., long-term forest demography or long-term nutrient limitations that may affect GPP), in a certain way it is mostly appropriate for intermediated temporal scales. Being optimistic, the analyzed 1982-2011 period, although quite long, is probably an upper limit of these intermediate scales.

Reviewer #2 (Remarks to the Author)

Second review of Recent increases in terrestrial carbon uptake at little cost to the water cycle for Nature Communications.

The updated manuscript has used a new dataset for evaluation of the WUE model. The improved statistics have helped the manuscript and I am more satisfied with this version. However I have some responses to my initial comments below (keeping the labeling format from the Authors' rebuttal), and some further questions. Overall though I think this manuscript would be suitable for Nature Communications if some further clarifications can be made.

B1: It's great to see that the new Fluxnet dataset has improved the evaluation of the WEC model. I am satisfied with this part of the manuscript and happy with the additional statistical information that is provided.

B2: Thank you for the detailed response. Much of my previous comment is no longer relevant since the large bias in WEC trend at the Fluxnet sites is reduced.

B3: (pasting author's response here for reference)
"Response: The control of LAI on the partitioning between transpiration and soil evaporation has been studied before (see Schulze et al. 19942, Kelliher, et al.3 and Wang et al., 20144). Experimental studies suggest that soil evaporation is negligible when canopy is closed (L>3), but it is also true that the canopy interception ratio (evaporation from wet canopy to E) is larger for higher L (see ref. 26). Therefore, it does not necessarily mean that the fraction of water use for production (i.e. Et/E) follows the exponential relationship of the partitioning between transpiration and soil evaporation."
I still have two questions/concerns about the role of increasing L in the WUE trends:
1. As the authors state in the response, the effect of L on soil evaporation saturates for LAI greater than ~3, implying that Et/E should also level out at this point. It makes sense that fE should also increase for higher L, but wouldn't this be accounted for in the evaporation datasets used to calculate fE? So my question is: How robust is the contribution of increasing LAI to the WUE trend? Are the LAIs above 3-4 artificially affecting results?
2. The trends obtained with the two LAI datasets are very different (17.5±1.9 mg C mm-1 H2O year-1 for GLASS and 10.0±1.6 mg C mm-1 H2O year-1 for GIMMS) (Supplemental Material). Is the step change around 2000 in GLASS strongly affecting the trend? Is this change a result of sensor changes or possibly due to something real? If it's the former, it's concerning that a sensor-related trend is strongly affecting the estimated GPP trend. I would like to see some more discussion of these uncertainties and their effects on the results. There should be some mention of the effect of different datasets in the main text. The fact that so many datasets have been used is a strength of the study, as is the ability to directly give uncertainty bounds on the estimated GPP and WUE and GPP trends (the usefulness of the WEC model in providing quantifiable uncertainty is already mentioned at line 199 of the main text).

I'm satisfied with author responses to my minor comments.

In the new manuscript, I have a few new comments and questions:
1. Looking at the review from Reviewer 3, I agree with their comment C2. I don't think the Friedlingstein et al. 2001 paper is adequate reference for GPP declining in the future, since that was from one model only. There are more recent references available such as the results from the last two C4MIP studies (e.g. Arora et al. 2013 Carbon–Concentration and Carbon–Climate Feedbacks in CMIP5 Earth System Models, Journal of Climate).

2. I would like to confirm that the two trends calculated by the WEC model are correct: 13.7 as calculated at the 11 Fluxnet sites, and 13.7 calculated based on all of the global datasets. It is odd that these are the same given they cover different spatial and temporal space, so worth double-checking.

3. The figures all look very good, but could units be added to the axes in Figure 1?

Reviewer #3 (Remarks to the Author)

The writing style of the manuscript has improved.
However, the evaluation of the model and the goodness of fit are still troubling in my mind. This is a very simplistic model, and the authors seems to rush to total global conclusions, which I am not convinced that are accurate or valid (e.g., GPP globally increased by 0.83±0.26 Pg C year^−2).

Particularly because the model is so simplistic and based on very spatially and temporally coarse data sources, I am having a hard time to accept the generality of the model validation with regards to spatial variation and accuracy, and its ability to predict trends.

Specifically,
1) I still do not accept the site list. Not sure if it was cherry-picked or just some strangely selected subset, or some strange data shortage in Fluxnet I am unaware of, but I am aware of MANY more FLUXNET stations with valid data and even with valid long-term data than listed here. For a quick example, compare with the list of sites that were included in the Keenan et al 2013 Paper (Figures S1 and S2), most of the sites with long-term observations that were used there are not included here (none is used for long term trend comparison), and none of the core 6 American sites Keenan focused on (Figure 3) are included here.
Are you using the processed La Thuile Dataset (there will be less observations there, and it arbitrarily stops the analysis at some point mid 2000) or the core Fluxnet data? Did you check Ameriflux for some site/years data missing in Fluxnet? Did you notice that among the sites you did chose, there are many agricultural sites and even some irrigated ones (Mead, Twitchell), for which your formulation must be totally wrong (unless you include irrigation in precip and include runoff from the field)? Similarly for wetland sites.

2) Your site selection is very sparse in many places and ecosystem types. For example you have only 2 tropical sites. Similarly, none in the arctic (Boreal and tundra ecosystems). While that is an unfortunate problem of global observation, I am having a hard time being convinced that your global projections that include large contributions and trends of these ecosystems have anything to do with reality. Many sites that you did not include in the validation contain observations for these latitudes and ecosystems.

3) What is "corrected LE data"? What kind of correction did you apply? Is it possible that your correction changed the energy closure of the site?

4) You are still using site data to calculate your model WUE that you then compare to observed. This is not acceptable. Your model validation should be based only on data that is available to the global product. Otherwise, your model is only valid where site measurements exist, and in these places, we have measurements of GPP and do not need your model. So, please use the global model temperature to determine the season, use the global models data for air temp and humidity to determine D, and use Ca from NOAA Mauna Loa site, as you do in the global model. I do not agree with the argument from the response letter that the site's footprint is small as a reason to use the observed data. While the footprint is small, it is assumed representative of the entire ecosystem around the tower. And while this assumption is not perfect, it is as reasonable as (and totally equivalent to) the assumptions taken by the gridded global datasets you use, which assume within-grid-cell uniformity of all environmental variables.

5) Please provide the coefficient of determination (R^2) and slope of the fit curve for figures 1a and 1b in the figure caption as well. Currently the text lists only the correlation coefficient (r) in the text. r is misleadingly high. If you square it (as you should) you'll get a goodness of fit of around r^2=0.45, and a Nash-Sutcliffe of 0.34, which are rather on the low side.
6) Is it possible that the entire trend validation is hinging on one outlier with very low WUE (fig 1b)? Seems to me that if you remove that one site, there will be no relationship at all.

Other comments:
Equation 5 in SI1 would be easier to figure out if you write it as: Et/(Et+Es)=1-exp(-kL)

Table S2 – please provide a way to relate the IGBP code to the vegetation type (and g1 values) that was used from Table S

Reviewer #1:

A1. The authors addressed satisfactorily most of the previous comments I made. A major revision in the manuscript is the use of a more accurate dataset (FLUXNET2015), which covers an extended period, for the validation of the analytical model (Eq. 1). The station selection and the way trends in annual WUE (Fig 1b) are compared is also different. This substitution of dataset improved the model validation and the confidence on the study. Of course, this is only related to the use of the dataset since ultimately the presented model and assumptions are the same. The unexplained variability remain close to 50% (Line 70 and Line 76), which I think needs a more explicit mention in the discussion.

Responses: We thank the reviewer for this insightful comment. As suggested, we have now stated explicitly the unexplained variance associated with our model (see lines 281-283) in the revised manuscript.

A2. I also see that trends in estimated GPP and WUE are much less sensitive to the assumptions of equation (1) and input data than the magnitude themselves (Supp, material section 6.2). I believe this is a point in favor of the study that should also be stated explicitly in the manuscript and not only implicitly in Figure 2a,b. I appreciated the discussion about the potential limitations of the analytical model and land surface models with regards to LAI (Line 240-243) but I would also suggest to state explicitly that a change in leaf area index cannot affect GPP directly in the analytical model. This is important and is not even discussed in the rebuttal, where only the ET dependence on LAI through input data is mentioned.

Responses: We thank the reviewer for the constructive comment. One more paragraph was added in the revised manuscript to discuss the influences of the uncertainty in different input datasets on the estimated magnitude and trends in WUE and GPP (see lines 251-266). The sensitivity of estimated WUE and GPP to LAI by the proposed method is now discussed (see lines 237-246).

A3. A potential explanation for the difference with the results of Keenan et al. 2013 is provided only in the rebuttal letter, and substantially the issue is omitted from the article but I conform to the authors' choice.

Responses: Essentially, estimated trend of this study is smaller than that derived by Keenan et al. 2013 likely due to different definition of the WUE and/or studied regions. We explained in the first round of revision and now we discussed this issue in the revised manuscript (see lines 283-288).

SPECIFIC COMMENTS

A4. Line 40. I respectfully disagree that is "difficult" to identify key controls on change in WUE from land-surface models. These are mechanistic models, therefore their results

should be traceable to some physical principle, process interaction or parameters. If this is not typically done, it is a different story.

Responses: Thanks for your insightful comments. We tried to argue that uncertainty in the WUE estimated by the land surface models are large due to significant uncertainties in both estimated GPP and E. We rewritten this sentence in the revised manuscript (see lines 38-41).

A5. Line 72-74. From the uncertainty bounds of trends in ecosystem WUE (close to 100% of the mean) provided in this version of the article, it emerges that site estimates of this quantity are very poorly constrained by current data. In a way, I am not surprised by this uncertainty, but I believe this is important and needs to be explicitly stated in the article.

Responses: The large uncertainty in the trends of observed WUE is (see Figure 1b) mainly resulted from two sources. One is that the 11 studied sites cover 4 different vegetation types and each vegetation type has different magnitudes of trends in WUE. The other reason is that one of the 11 sites has a significant negative trend in WUE. If the site with negative trend is excluded, the estimated uncertainty of the annual WUE trend is significantly reduced. The average observed and estimated trends of the 10 sites become 22.3±6.5 and 21.2±9.0 mg C mm^{-1} H_2O $year^{-1}$, respectively.

We agree with the reviewer that evidence of positive trend in WUE from observed flux data subjects to significant uncertainty, which is explicitly stated in the revised manuscript (see lines 283-288).

A6. Line 202-204. I respectfully disagree on the fact that nutrient availability or soil water changes necessarily act on temporal scales finer than the year. Actually, I would argue that the presented approach neglect fine temporal scale and also long-term adaptations (e.g., long-term forest demography or long-term nutrient limitations that may affect GPP), in a certain way it is mostly appropriate for intermediated temporal scales. Being optimistic, the analyzed 1982-2011 period, although quite long, is probably an upper limit of these intermediate scales.

Responses: We agree with the reviewer's insightful comment. We now included this valuable comment about time scale in the Discussion (see lines 273-279 in the revised manuscript).

Reviewer #2:

Second review of Recent increases in terrestrial carbon uptake at little cost to the water cycle for Nature Communications.

The updated manuscript has used a new dataset for evaluation of the WUE model. The improved statistics have helped the manuscript and I am more satisfied with this version. However I have some responses to my initial comments below (keeping the labeling format from the Authors' rebuttal), and some further questions. Overall though I think this manuscript would be suitable for Nature Communications if some further clarifications can be made.

B1. B1: It's great to see that the new Fluxnet dataset has improved the evaluation of the WEC model. I am satisfied with this part of the manuscript and happy with the additional statistical information that is provided.

B2: Thank you for the detailed response. Much of my previous comment is no longer relevant since the large bias in WEC trend at the Fluxnet sites is reduced.

Responses: Thank you.

B3: (pasting author's response here for reference)

"Response: The control of LAI on the partitioning between transpiration and soil evaporation has been studied before (see Schulze et al. 19942, Kelliher, et al.3 and Wang et al., 20144). Experimental studies suggest that soil evaporation is negligible when canopy is closed (L>3), but it is also true that the canopy interception ratio (evaporation from wet canopy to E) is larger for higher L (see ref. 26). Therefore, it does not necessarily mean that the fraction of water use for production (i.e. Et/E) follows the exponential relationship of the partitioning between transpiration and soil evaporation."

I still have two questions/concerns about the role of increasing L in the WUE trends:

B2. As the authors state in the response, the effect of L on soil evaporation saturates for LAI greater than ~3, implying that Et/E should also level out at this point. It makes sense that fE should also increase for higher L, but wouldn't this be accounted for in the evaporation datasets used to calculate fE? So my question is: How robust is the contribution of increasing LAI to the WUE trend? Are the LAIs above 3-4 artificially affecting results?

Responses: According to our proposed WUE model, the influence of leaf area index (*L*) on ecosystem WUE is accounted in the second term of the equation (1) directly (i.e. $[1 - exp(-kL)]$) and in the third term indirectly (i.e. $(1 - f_{E_i})$). The second term predicts that ecosystem WUE increases with *L* and this relationship saturates quickly when *L* > 3. The third term can lower ecosystem WUE as *L* increases because canopy interception increases with *L*.

Essentially, the second and third terms together represent the fraction of transpiration (E_t) to total evaporation (i.e. f_{E_t}, transpiration ratio) as:

$$f_{E_t} = \frac{E_t}{E} = \frac{E_t}{E_t + E_s} \frac{E_t + E_s}{E_t + E_s + E_i} = \frac{E_t}{E_t + E_s}\left(1 - \frac{E_i}{E}\right) = [1 - exp(-kL)](1 - f_{E_i})$$

To demonstrate the control of LAI on ecosystem WUE, we plot the relationship between mean annual leaf area index (L) and transpiration ratio (f_{E_t}) based on the leaf area index data from GIMMS LAI3g and interception ratio data from E_{GLEAM} and in Figure R1.

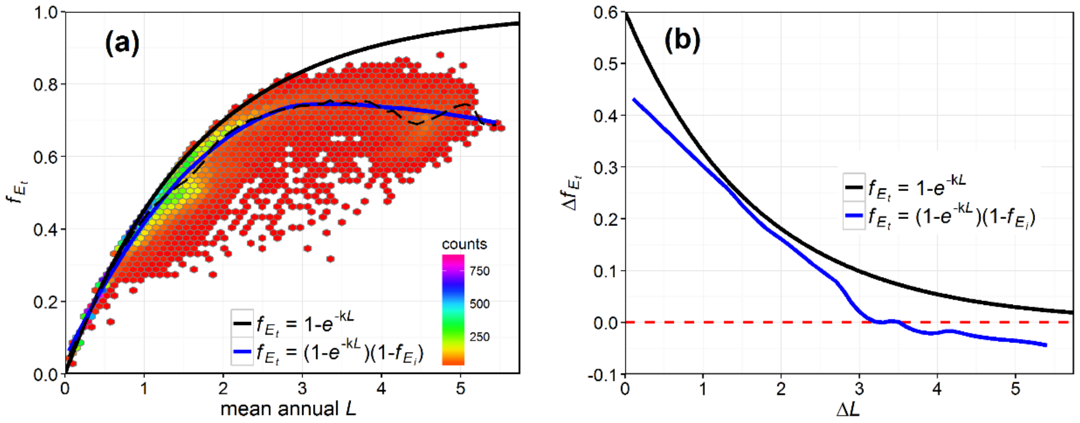

Figure R1 | The control of leaf area index (L) on ecosystem WUE through the transpiration ratio (f_{E_t}). (a) Hexagon binning plot showing relationship between mean annual L and f_{E_t} over all the vegetated land cells. The colour of the hexagon indicates the number of land cells. The broken black line is the median of f_{E_t} by binning mean annual L with a step of 0.1, and the blue line is smoothed median f_{E_t} (i.e. broken line) using non-parametric local regression method (i.e. LOESS). On average, the blue line reflects the control of L on the estimated ecosystem WUE, i.e. $f_{E_t} = [1 - exp(-kL)](1 - f_{E_i})$. The solid black line shows the relationship between f_{E_t} and L by neglecting the interception ratio, i.e. $f_{E_t} = [1 - exp(-kL)]$. (b) The derivative lines of two different forms of f_{E_t} in the subplot (a).

Figure R1 shows that our model predicts that the control of L on ecosystem WUE increases with L to about ~3 but decreases with L when L is larger than 3. Figure R1 also suggests that the control of L on ecosystem WUE does not saturate when the L > 3 as predicted by the term $[1 - exp(-kL)]$ alone. Therefore, our estimated WUE and GPP are not artificially affected by the adopted first order partitioning principle between transpiration and soil evaporation (i.e. $1 - exp(-kL)$) when LAI is above 3. The uncertainty of contribution of LAI to the trend in WUE and GPP are 4.3 mg C mm^{-1} H_2O year^{-1} and 0.24 Pg C year^{-2}, compared to the mean contribution of 7.4 mg C mm^{-1} H_2O year^{-1} and 0.41 Pg C year^{-2}, respectively.

In the revised manuscript, we discussed the control of leaf area index on the modelled ecosystem WUE and provided the figure R1 in the supplementary materials. Please refer to lines 237-246.

B3. 2. The trends obtained with the two LAI datasets are very different (17.5±1.9 mg C mm-1 H2O year-1 for GLASS and 10.0±1.6 mg C mm-1 H2O year-1 for GIMMS) (Supplemental Material). Is the step change around 2000 in GLASS strongly affecting the trend? Is this change a result of sensor changes or possibly due to something real? If it's the former, it's concerning that a sensor-related trend is strongly affecting the estimated GPP trend.

I would like to see some more discussion of these uncertainties and their effects on the results. There should be some mention of the effect of different datasets in the main text. The fact that so many datasets have been used is a strength of the study, as is the ability to directly give uncertainty bounds on the estimated GPP and WUE and GPP trends (the usefulness of the WEC model in providing quantifiable uncertainty is already mentioned at line 199 of the main text).

Responses: We have communicated with the leading author of GLASS LAI products. The step change around 2000 is likely due to the algorithms to fuse products from two different sensors. But this problem is mainly limited to tropical regions. As suggested, one more paragraph is added in the revised manuscript to discuss the uncertainties in different datasets, which has been discussed in the supplementary materials only in previous revision. Please refer to lines 251-266 in the revised manuscript.

I'm satisfied with author responses to my minor comments.

In the new manuscript, I have a few new comments and questions:

B4. Looking at the review from Reviewer 3, I agree with their comment C2. I don't think the Friedlingstein et al. 2001 paper is adequate reference for GPP declining in the future, since that was from one model only. There are more recent references available such as the results from the last two C4MIP studies (e.g. Arora et al. 2013 Carbon–Concentration and Carbon–Climate Feedbacks in CMIP5 Earth System Models, Journal of Climate).

Responses: Thank you for bringing this to our attention. We have cited this references in the revised manuscript to support this study.

B5. 2. I would like to confirm that the two trends calculated by the WEC model are correct: 13.7 as calculated at the 11 Fluxnet sites, and 13.7 calculated based on all of the global datasets. It is odd that these are the same given they cover different spatial and temporal space, so worth double-checking.

Responses: These two average values happen to be the same. However, they have different variabilities and represent two different study regions.

B6. 3. The figures all look very good, but could units be added to the axes in Figure 1?

Responses: Thanks. As suggested, the unit is provided in the Figure 1.

Reviewer #3:

The writing style of the manuscript has improved.

C1. However, the evaluation of the model and the goodness of fit are still troubling in my mind. This is a very simplistic model, and the authors seems to rush to total global conclusions, which I am not convinced that are accurate or valid (e.g., GPP globally increased by 0.83±0.26 Pg C year^−2). Particularly because the model is so simplistic and based on very spatially and temporally coarse data sources, I am having a hard time to accept the generality of the model validation with regards to spatial variation and accuracy, and its ability to predict trends.

Responses: The evidence we presented show a broad agreement between our model simulations and observations for annual WUE across 243 site-years and for the WUE tends of 11 sites. We now added some discussions about the applicability of our model across different time scales, as suggested by reviewer 1 (see our response to A6). In addition, and although not proof, our finding for a significant increased GPP is consistent with the evolution of the global carbon budget over the past 30 years which shows increased terrestrial carbon sink (see IPCC report, 2013).

Specifically,

C2. I still do not accept the site list. Not sure if it was cherry-picked or just some strangely selected subset, or some strange data shortage in Fluxnet I am unaware of, but I am aware of MANY more FLUXNET stations with valid data and even with valid long-term data than listed here. For a quick example, compare with the list of sites that were included in the Keenan et al 2013 Paper (Figures S1 and S2), most of the sites with long-term observations that were used there are not included here (none is used for long term trend comparison), and none of the core 6 American sites Keenan focused on (Figure 3) are included here.

Responses: The selected sites for model validation were in fact not cherry-picked. The criteria used for selecting sites are clearly stated in the Section 3 of the Supplementary Materials.

Here we demonstrate the changes in flux data selected for model validation after the following three basic criteria are applied:

(A). Based on the data quality information in the FLUXNET2015 dataset, site-year with >80% of high-quality observed or gap-filled data of hourly GPP and LE;
(B). At least one pair of annual corrected LE and GPP are available;
(C). Energy closure bias is within 20%, i.e. $0.8 < LE_{corrected}/LE < 1.2$, where LE is observed latent flux and and the $LE_{corrected}$ is corrected LE.

High quality data of annual GPP and LE is used as one of the selection criteria because we define the annual WUE in this study as the ratio of annual total GPP and annual total evapotranspiration (E), which makes this study different from other studies that annual WUE

is defined using summer months (or growing season) data, therefore some of the data used by Keenan et al. (2013) were not used in this study.

Correction of LE is necessary as lack of energy closure is quite common in observed latent fluxes using eddy covariance techniques. If flux observation underestimates LE by 20%, it can lead to a significant overestimation of WUE by up to 25%. The corrected LE data are provided in the FLUXNET2015 dataset, which is based on the energy balance closure ratio and keep the Bowen ratio unchanged.

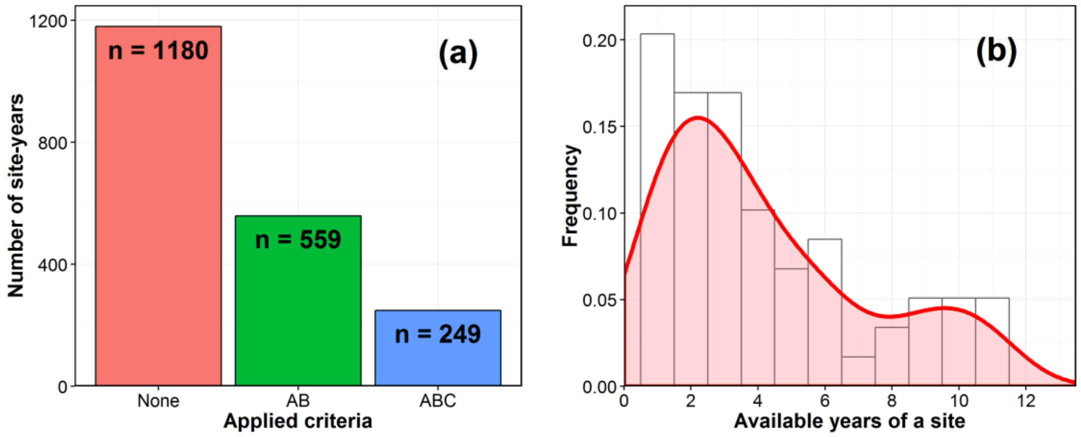

Figure R2 | Data available for model validation as affected by different selection criteria. (a) Changes of the available site-year data by applying criteria none, AB or ABC. The first column shows the total 1180 site-years of data available in the FLUXNET2015 dataset. The 2nd and 3rd bars indicate the number of site-years left after applying selection criteria A and B (i.e. AB) and all the three basic criteria (i.e. ABC), respectively. (b) Fraction of site with the length of data in year after three selection criteria are applied.

Figure R2 suggests that we have used as much data as possible in the latest global flux dataset for validating the simple WUE model. Figure R2 shows that only 249 site-years data are left for model validation after considering the three basic criteria described above (Figure R2a), of which the majority of the sites only have shorter than 6 years of data for trend validation (Figure R2b). A few site-years data are further excluded by other selection criteria and available of other data sources for validation (see Supplementary Materials section 3 for more information). In summary, 55 sites and 243 site-years of data are used for model validation.

None of the seven core sites from the AmeriFlux used by Keenan et al 2013 Paper (i.e. US-Ha1, US-Ha2, US-LPH, US-Ho1, US-MMS, US-UMB and US-Bar) is included in this study due to (1) four of the seven sites (i.e. US-Ha2, US-LPH, US-Ho1 and US-Bar) are not included in the FLUXNET2015 dataset; (2) other two sites (i.e. US-Ha1 and US-UMB) are without available corrected LE or other energy fluxes observations to correct LE; and (3) the rest site (i.e. US-MMS) has significant annual energy closure bias, of which average value of $LE_{corrected}/LE$ is 1.45 with a range from 1.40 to 1.52.

C3. Are you using the processed La Thuile Dataset (there will be less observations there, and it arbitrarily stops the analysis at some point mid 2000) or the core Fluxnet data? Did you check Ameriflux for some site/years data missing in Fluxnet? Did you notice that among the sites you did chose, there are many agricultural sites and even some irrigated

ones (Mead, Twitchell), for which your formulation must be totally wrong (unless you include irrigation in precip and include runoff from the field)? Similarly for wetland sites.

Responses: Previously, we used the La Thuile Dataset and collected additional sites and/or site-years data from Ameriflux and other regional flux networks that are not included in the La Thuile Dataset. Most of the sites from the La Thuile Dataset span a few years and are up to about 2011. We spent a lot of time to collect and process the data but data for trend validation was very limited (as provided in the first round of review). Fortunately, global FLUXNET team just released the latest collection of global flux data up to 2014 with standard data processing procedures applied to all the sites. That collection, or FLUXNET2015, includes most of the Ameriflux and La Thuile Dataset, and are used to validate the model in this version. Please refer to the site list of the FLUXNET2015 at http://fluxnet.fluxdata.org/sites/site-list-and-pages/. Site selection is based on the TIER TWO FULLSET of FLUXNET2015 dataset. Criteria for data selection please refer to Section 3 of supplementary materials and our response to C2.

The FLUXNET2015 datasets is the latest data product provided by the international flux community and used for this study. We did not try to include more data from other regional fluxnet communities in the validation for three reasons: (1) previously we found that discrepancy in the annual WUE derived from flux data processed by different groups are quite significant; (2) the FLUXNET2015 dataset is processed by applying same data processing protocol to all sites for consistency; and (3) the FLUXNET2015 dataset provides several estimates of GPP using different methods, which are very valuable for us to quantified the uncertainty in observed GPP and WUE.

We disagree with the review that the proposed WUE model for the agriculture or wetland sites are totally wrong. Our model is not based on the requirement of ecosystem total water use (i.e. E) to estimate WUE and thus is not subject to the issue that WUE might be biased by additional available water for ecosystem use (e.g., irrigation and runoff as mentioned by the reviewer). Furthermore, the number of agricultural sites we selected is relatively small (7 of 55 sites, see Supplementary Table S2). The estimated increasing trend in WUE and its attributions in the agricultural ecosystems are consistent with recently published literatures (e.g., Long et al. (2006); Lobell and Field (2008); Leakey et al. (2009); Drewry et al. (2010); Piao et al. (2010); Lobell et al. (2011); Deryng et al. (2016), *etc.*).

The seven E products used in this study are all diagnostic and reanalysis products, which are estimated based on a wide variety of remotely sensed and climate forcing datasets (see Jung et al. (2010); Miralles et al. (2010); Miralles et al. (2011); Miralles et al. (2014); Zeng et al. (2014); Martens et al. (2016); Zhang et al. (2016), *etc.*). Although uncertainty of E in the agricultural ecosystems from these products may be large, they are probably the best E products currently available at the global scale. In summary, this study may subject to some potential limitations due to human interferences on the carbon and water cycles but they are not significant enough to undermine the overall global trends in WUE and GPP reported in this study.

C4. 2) Your site selection is very sparse in many places and ecosystem types. For example you have only 2 tropical sites. Similarly, none in the arctic (Boreal and tundra ecosystems). While that is an unfortunate problem of global observation, I am having a hard time being convinced that your global projections that include large contributions

and trends of these ecosystems have anything to do with reality. Many sites that you did not include in the validation contain observations for these latitudes and ecosystems.

Responses: We tried to use as many sites as possible to validate the model (see our response to the comment C2 and C3). Unfortunately no data that meet our selection criteria are available for model validation from tropical or boreal forest regions. We have now stated this limitation explicitly in the revised manuscript (see lines 285-288).

C5. 3) What is "corrected LE data"? What kind of correction did you apply? Is it possible that your correction changed the energy closure of the site?

Responses: The corrected LE data means the observed site LE is corrected by energy closure ratio based on the assumption that observed Bowen ratio is correct. Correction of LE is necessary as lack of energy closure is quite common in observed latent flux using eddy covariance techniques. The corrected LE is provided in the FLUXNET2015 dataset and more information can be found at http://fluxnet.fluxdata.org/data/fluxnet2015-dataset/data-processing/. The corrected LE is explained and more information is provided in the revised supplementary file.

C6. 4) You are still using site data to calculate your model WUE that you then compare to observed. This is not acceptable. Your model validation should be based only on data that is available to the global product. Otherwise, your model is only valid where site measurements exist, and in these places, we have measurements of GPP and do not need your model. So, please use the global model temperature to determine the season, use the global models data for air temp and humidity to determine D, and use Ca from NOAA Mauna Loa site, as you do in the global model. I do not agree with the argument from the response letter that the site's footprint is small as a reason to use the observed data. While the footprint is small, it is assumed representative of the entire ecosystem around the tower. And while this assumption is not perfect, it is as reasonable as (and totally equivalent to) the assumptions taken by the gridded global datasets you use, which assume within-grid-cell uniformity of all environmental variables.

Responses: Three site observed variables are used to estimate ecosystem WUE at the site level, while the other four variables are the same with that used for estimating global WUE. The three site observed variables are air temperature (T_a), CO_2 concentration (C_a) and vapour pressure deficit (D). The first variable, i.e. T_a, is used as an indicator for growing season, while the last two variables, i.e. C_a and D, are the input for the WUE model. However, our validation is not very sensitive to whether the site data or global data are used for those three variables.

Figure R3 shows the relationship between two estimates of annual WUE across all flux sites we selected using two different data sources, i.e. three variables are site observed (as we did for validation) or from global datasets. The linear correlation coefficient (r) between observed and estimated annual WUE is about 0.93, with Nash-Sutcliffe model efficiency (NSE) of 0.83, root mean squared error (RMSE) of 0.46 g C mm^{-1} H_2O, mean error (ME) of −0.16 g C mm^{-1} H^2O and relative error (RE) of −7.9%. The slope of the regressed line between the two estimates passing through the origin is 0.92, with an adjusted R^2 of 0.97. Meanwhile, the linear correlations between gridded and site observed air temperature (T_a) and vapour pressure

deficit (*D*) are very high. The adjusted R^2 of the linear regression passing through the origin between gridded and site observed monthly *D* and T_a is 0.95 and 0.99, respectively. Therefore, we believe that use of global dataset for validation will not significantly change our validation results and the conclusion.

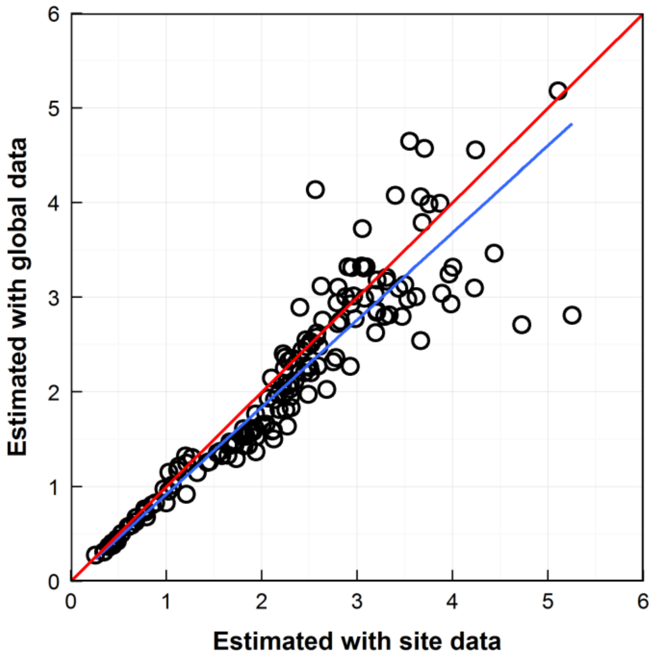

Figure R3 | Estimated annual WUE at flux sites using two different data sources. One is estimated using all the inputs identified from global datasets (as plotted on y-axis), and the other one with three variables observed at site level (as plotted on x-axis). The red line is 1:1 and the blue is the best-fitted linear regression passing through the origin using the least square method. The two annual WUE estimates is in g C mm^{-1} H_2O.

Three site observed variables are used for estimating site-level WUE because (1) they are routinely observed variables at flux sites and are readily available in the FLUXNET2015 sites; (2) site level observed data are more representative than global gridded data for site level conditions, although there is an assumption that meteorological conditions are uniform within the footprint of flux tower and within the grid cell of global forcing dataset; and (3) importantly, collected global forcing datasets are only up to 2011, while FLUXNET2015 dataset is up to 2014. The number of sites for validation will be reduced if global forcing data are used, especially for trend validation. Therefore, we still used site data of T_a, C_a and *D* for model validation.

C7. 5) Please provide the coefficient of determination (R^2) and slope of the fit curve for figures 1a and 1b in the figure caption as well. Currently the text lists only the correlation coefficient (r) in the text. r is misleadingly high. If you square it (as you should) you'll get a goodness of fit of around r^2=0.45, and a Nash-Sutcliffe of 0.34, which are rather on the low side.

Responses: As suggest, R^2 and slope of regressed line passing through the origin are provided in the revised manuscript (see lines 71-73 and 78-80) and also in the supplementary materials (see section 3.3).

C8. 6) Is it possible that the entire trend validation is hinging on one outlier with very low WUE (fig 1b)? Seems to me that if you remove that one site, there will be no relationship at all.

Responses: We agree that the including the outlier in the Figure 1b improved the overall statistic of the validation to some extent. Excluding this outlier, the mean observed and estimated trend in WUE of the remaining 10 sites are 22.3±6.5 and 21.2±9.0 mg C mm^{-1} H_2O $year^{-1}$, respectively, with a linear correlation coefficient (r) of 0.43, relative error (RE) of −5.1%, root mean squared error (RMSE) of 25.8 mg C mm^{-1} H_2O $year^{-1}$, mean error (ME) of −1.15 mg C mm^{-1} H_2O $year^{-1}$ and a negative of Nash-Sutcliffe model efficiency (NSE). It is obvious that r and NSE are degraded if that outlier is excluded, but other statistic measures do not change much. Nonetheless, we have no obvious reason to exclude this site from the validation. Both observed and estimated trends in WUE range from negative to positive, which is consistent with our global results that trends in WUE vary significantly from negative to positive regionally.

In the revised manuscript, we have now discussed the uncertainties in the annual WUE trend as estimated from observed flux data (see lines 281-288).

Other comments:

C9. Equation 5 in SI1 would be easier to figure out if you write it as: Et/(Et+Es)=1-exp(-kL)

Responses: Thanks. Change is made accordingly.

C10. Table S2 – please provide a way to relate the IGBP code to the vegetation type (and g1 values) that was used from Table S

Responses: As suggested, the website for detailed IGBP code is now provided and the g_1 values are now included in the table S2.

References

Deryng, D., Elliott, J., Folberth, C., Muller, C., Pugh, T.A.M. and Boote, K.J. et al., 2016. Regional disparities in the beneficial effects of rising CO_2 concentrations on crop water productivity. Nature Clim. Change, advance online publication.

Drewry, D.T., Kumar, P., Long, S., Bernacchi, C., Liang, X.Z. and Sivapalan, M., 2010. Ecohydrological responses of dense canopies to environmental variability: 2. Role of acclimation under elevated CO_2. J. Geophys. Res., 115(G4): G04023.

Jung, M., Reichstein, M., Ciais, P., Seneviratne, S.I., Sheffield, J. and Goulden, M.L. et al., 2010. Recent decline in the global land evapotranspiration trend due to limited moisture supply. Nature, 467(7318): 951-954.

Leakey, A.D.B., Ainsworth, E.A., Bernacchi, C.J., Rogers, A., Long, S.P. and Ort, D.R., 2009. Elevated CO_2 effects on plant carbon, nitrogen, and water relations: Six important lessons from FACE. J. Exp. Bot., 60(10): 2859 -2876.

Lobell, D.B. and Field, C.B., 2008. Estimation of the carbon dioxide (CO_2) fertilization effect using growth rate anomalies of CO_2 and crop yields since 1961. Global Change Biol., 14(1): 39-45.

Lobell, D.B., Schlenker, W. and Costa-Roberts, J., 2011. Climate Trends and Global Crop Production Since 1980. Science, 333(6042): 616 -620.

Long, S.P., Ainsworth, E.A., Leakey, A.D.B., Nösberger, J. and Ort, D.R., 2006. Food for Thought: Lower-Than-Expected Crop Yield Stimulation with Rising CO_2 Concentrations. Science, 312(5782): 1918.

Martens, B., Miralles, D.G., Lievens, H., van der Schalie, R., de Jeu, R.A.M. and Férnandez-Prieto, D. et al., 2016. GLEAM v3: Satellite-based land evaporation and root-zone soil moisture. Geosci. Model Dev. Discuss., 2016: 1-36.

Miralles, D.G., Gash, J.H., Holmes, T.R.H., de Jeu, R.A.M. and Dolman, A.J., 2010. Global canopy interception from satellite observations. Journal of Geophysical Research: Atmospheres, 115(D16): D16122.

Miralles, D.G., Holmes, T.R.H., De Jeu, R.A.M., Gash, J.H., Meesters, A.G.C.A. and Dolman, A.J., 2011. Global land-surface evaporation estimated from satellite-based observations. Hydrol. Earth Syst. Sc., 15(2): 453.

Miralles, D.G., van den Berg, M.J., Gash, J.H., Parinussa, R.M., de Jeu, R.A.M. and Beck, H.E. et al., 2014. El Niño – La Niña cycle and recent trends in continental evaporation. Nature Clim. Change, 4(2): 122-126.

Piao, S., Ciais, P., Huang, Y., Shen, Z., Peng, S. and Li, J. et al., 2010. The impacts of climate change on water resources and agriculture in China. Nature, 467(7311): 43-51.

Zeng, Z., Wang, T., Zhou, F., Ciais, P., Mao, J. and Shi, X. et al., 2014. A worldwide analysis of spatiotemporal changes in water balance-based evapotranspiration from 1982 to 2009. Journal of Geophysical Research: Atmospheres, 119(3): 1186-1202.

Zhang, Y., Peña-Arancibia, J.L., McVicar, T.R., Chiew, F.H.S., Vaze, J. and Liu, C. et al., 2016. Multi-decadal trends in global terrestrial evapotranspiration and its components. Sci. Rep.-UK, 6: 19124.

Reviewers' Comments:

Reviewer #1 (Remarks to the Author)

I am mostly fine with how the authors revised the manuscript and I appreciate some addition (e.g., Line 282-285). The presented analytical model is very simple, the assumptions are numerous and the overall explained variability in WUE and WUE trends is admittedly relatively low (Fig 1); however this is the first time hydrological variables are used to constrain WUE and GPP and the obtained results will likely contribute to the ongoing discussion about trends in WUE and global estimates of GPP.

Line 76. In this line I would suggest to delete the reference to Keenan et al. 2013, since it is misused and it is also contradictory with lines 283-285.

Line 88. I would not say that it is close but that it is 25% larger; this is another estimate, higher than previous estimates of global WUE.

Line 195. Beginning of the sentence "are" rather than "is".

Line 236. Following my previous comment, which was eluded, I would also state explicitly that a change in leaf area index cannot affect GPP directly in the analytical model.

Line 295. The sentence in the bracket is missing "there", e.g., "but there can be significant…"

Figure S6. I probably did not pay attention to the potential shape of fEt until it is has been presented explicitly in Fig. S6 and in the rebuttal letter. Such a strong and linear dependence between L and ecosystem transpiration divided by total evapotranspiration for $L<3$ is completely unsupported by observations (e.g., Wang et al 2014) and reinforces the idea that the assumptions of the analytical model are very strong. I hope this can be also highlighted.

Wang, L., S. P. Good, and K. K. Caylor (2014), Global synthesis of vegetation control on evapotranspiration partitioning, Geophys. Res. Lett., 41, 6753–6757, doi:10.1002/2014GL061439.

Reviewer #2 (Remarks to the Author)

I am satisfied with the authors responses to my questions and the additional explanation included in the text.

Reviewer #3 (Remarks to the Author)

With the current revision, I think we are getting closer, but it is also becoming clearer where are the core areas of disagreement.

I am willing to concede about the site list. You response convinced me that there was no intentional or unintentional cherry-picking. I am very familiar with fluxnet2015 and the process of its generation. The choice of which sites are included in FLUXNET2015 and which not, and for which sites the corrected evaporation was or was not released with the level2 dataset is rather arbitrary and represents a very restrictive subset of the globally available data. However, that process was not made by the authors. While lots of other data exist, and could have improved (or dis-validate) the evaluation of the trend estimation with your model, fluxnet2015 is the most advanced and expansive flux synthesis dataset to date. Considering the advantages of a uniform

analysis approach as presented by fluxnet over site-by-site data analysis, I can see the merit in restricting the site list to the fluxnet reanalysis.

Were I totally disagree with the authors is about using observed site-level data as model input in the model validation.
The key point here is that in this paper your model is not used as a site-level model. You apply it globally. therefore, in order to validate your global estimate, you must show that the result of the process you use in the global estimate is valid.
You create observation-driven prediction for site level data, and validate it with site observations. While it may infer of your model ability to predict local GPP given local observed forcing, it says nothing about the validity, accuracy or precision of the global approach that you use, which uses other data as input, which carry additional and presumably large sources of uncertainty.
In effect, your global gridded model already includes the model predictions for observed flux-site locations using only the global datasets. Please use these data in the manuscript for direct validation of your global estimate approach.
The argument that the global data availability only reaches 2011 is not a very strong one. You can get VPD, temp and precip estimates from ECMWF at relatively high resolution (much better than 0.5 degrees). Will just take a little bit more work to process...

Agricultural sites are more affected by management practices than by anything else (cropping cycle, tillage, fertilizer use...). None of the global data products you use as input to the global model land-use include agricultural management practices, and I therefore suspect that your predictive ability in the particular agricultural sites you use is strongly driven by incorporating the observations in these sites, and is not representative of global ability to predict GPP in agricultural fields. Another big problem with irrigated fields is that your E estimates are totally wrong for these fields. Your E is based on mass balance. If the irrigation water comes only from very near local sources it would have been fine, but if river, lake or reservoir water is used in irrigation are pumped from far watersheds, or from deep aquifers, the data you include for E will not include these water resources as an input for these location, and therefore, your estimate will be way off.

Finally, your main conclusion, highlighted in the abstract, results and discussion section is the bulk global number of GPP increase.
However, given the scarcity of the observation data that could validate this number, particularly with regards to your model ability to detect trends I find this number highly improper and unjustified. To a large degree (as you admit) the global trend you determine is driven by one extreme site observation. Further more, you have no more that 2 points for many ecosystem types.
You should consider a more extensive sensitivity analysis using a Monte Carlo approach of random re-sampling. For example, consider the spread of data among and within points of similar forest types in your figure 1b. If you draw a random sub-set from these points (including the error estimate for each point) you'll find that the slope of the decal trend may vary over a huge range. You should further carry this uncertainty to the global estimate, assuming each point in the global grid can vary within this range. You'll find that the error estimate you provide for your global estimate is highly unrealistic and underestimates the real uncertainty in your process.
It'll be better if your restrict your conclusions to the areas where you do have strong representation of observed data such as mid-latitude forests, and ignore other areas (tropical, boreal, agricultural, semi-arid...)

Furthermore, there are MANY papers that use remote sensing and global models to estimate the decadal changes in GPP. can you place your estimates within the spread of estimates by these other methods? Are you predicting something extreme and different, or are you within the commonly expected range?
That should be done for eco-regions, not only (or not at all) the global number.

Minor comment - in figure 1b please plot the actual trend model-observed trend line, and its

margins of error so that it could be compared with the 1:1 line. showing only the 1:1 line is misleading.

Figures 1c-d, 3 and 4 - please state clearly in the captions that these are model predictions. Currently it will mislead readers to assume these are observations.

Reviewer #1 (Remarks to the Author):

I am mostly fine with how the authors revised the manuscript and I appreciate some addition (e.g., Line 282-285). The presented analytical model is very simple, the assumptions are numerous and the overall explained variability in WUE and WUE trends is admittedly relatively low (Fig 1); however this is the first time hydrological variables are used to constrain WUE and GPP and the obtained results will likely contribute to the ongoing discussion about trends in WUE and global estimates of GPP.

A1. Line 76. In this line I would suggest to delete the reference to Keenan et al. 2013, since it is misused and it is also contradictory with lines 283-285.

Responses: Thanks. As suggested, reference to Keenan et al. 2013 was deleted in the revised manuscript.

A2. Line 88. I would not say that it is close but that it is 25% larger; this is another estimate, higher than previous estimates of global WUE.

Responses: Thanks. Change has been made as suggested. Please refer to lines 88-89 in the revised manuscript.

A3. Line 195. Beginning of the sentence "are" rather than "is".

Responses: Thanks for pointing out this typo. Change has been made as suggested.

A4. Line 236. Following my previous comment, which was eluded, I would also state explicitly that a change in leaf area index cannot affect GPP directly in the analytical model.

Responses: Thanks for this insightful comment. We revised the sentence by stating explicitly that change in L on GPP is indirectly accounted for in the data products of E and estimated WUE. Please refer to line 247 in the revised manuscript.

A5. Line 295. The sentence in the bracket is missing "there", e.g., "but there can be significant…"

Responses: Thanks. Change has been made as suggested.

A6. Figure S6. I probably did not pay attention to the potential shape of fEt until it is has been presented explicitly in Fig. S6 and in the rebuttal letter. Such a strong and linear dependence between L and ecosystem transpiration divided by total evapotranspiration for L<3 is completely

unsupported by observations (e.g., Wang et al 2014) and reinforces the idea that the assumptions of the analytical model are very strong. I hope this can be also highlighted.

Responses: The relationship in the Figure S6 is not a linear function of *L*. Wang et. al, 2014 reviewed the published papers on the relationship between T/E (i.e. transpiration/total E) and L and showed the relationship can be approximated as a nonlinear function of L, which is similar to what we used in this study. We also acknowledged that the relationship between T/E and L is more complex in reality and other factors may affects the partitioning (Wang et al., 2014). Changes have been made in lines 255-256, and lines 287-288 in the revised manuscript and lines 47-55 in the supplementary materials to make clear the control of leaf area index on E partition is much more complex in reality.

Wang, L., S. P. Good, and K. K. Caylor (2014), Global synthesis of vegetation control on evapotranspiration partitioning, Geophys. Res. Lett., 41, 6753–6757, doi:10.1002/2014GL061439.

Reviewer #2 (Remarks to the Author):

I am satisfied with the authors responses to my questions and the additional explanation included in the text.

We are pleased Reviewer#2 finds the revised version acceptable.

.

Reviewer #3 (Remarks to the Author):

With the current revision, I think we are getting closer, but it is also becoming clearer where are the core areas of disagreement.

C1. I am willing to concede about the site list. You response convinced me that there was no intentional or unintentional cherry-picking. I am very familiar with fluxnet2015 and the process of its generation. The choice of which sites are included in FLUXNET2015 and which not, and for which sites the corrected evaporation was or was not released with the level2 dataset is rather arbitrary and represents a very restrictive subset of the globally available data. However, that process was not made by the authors. While lots of other data exist, and could have improved (or dis-validate) the evaluation of the trend estimation with your model, fluxnet2015 is the most advanced and expansive flux synthesis dataset to date. Considering the advantages of a uniform analysis approach as presented by fluxnet over site-by-site data analysis, I can see the merit in restricting the site list to the fluxnet reanalysis.

Responses: Thanks.

C2. Were I totally disagree with the authors is about using observed site-level data as model input in the model validation.

The key point here is that in this paper your model is not used as a site-level model. You apply it globally. therefore, in order to validate your global estimate, you must show that the result of the process you use in the global estimate is valid.

You create observation-driven prediction for site level data, and validate it with site observations. While it may infer of your model ability to predict local GPP given local observed forcing, it says nothing about the validity, accuracy or precision of the global approach that you use, which uses other data as input, which carry additional and presumably large sources of uncertainty.

In effect, your global gridded model already includes the model predictions for observed flux-site locations using only the global datasets. Please use these data in the manuscript for direct validation of your global estimate approach.

The argument that the global data availability only reaches 2011 is not a very strong one. You can get VPD, temp and precip estimates from ECMWF at relatively high resolution (much better than 0.5 degrees). Will just take a little bit more work to process...

Responses: As suggested, validation results using global forcing datasets are provided in the revised manuscript. We collected latest version of CRU-NCEP dataset for the validation, of which available VPD is up to 2014. The validity of the analytical WUE model is well supported by the new validation results, although statistic measures degraded slightly compared with validation results using site data (i.e. results of previous version).

New results are provided in the Figure 1a and 1b in the revised manuscript. For the spatial validation (i.e. Figure 1a), the linear correlation between observed and estimated ecosystem WUE of 229 site-years is about 0.64, with a relative bias of −10.6% and mean error of −0.26 g C mm^{-1} H_2O. The slope of

the regressed line between observed and estimated annual WUE passing through the origin is 0.84, with an adjusted R^2 of 0.87. For the trend validation (i.e. Figure 1b), the estimated mean trend in ecosystem WUE is about 14.7±9.0 mg C mm^{-1} H_2O year^{-1} (mean ± 1 standard error), which is consistent with the in situ observed mean trend of 12.6±11.4 mg C mm^{-1} H_2O year^{-1}. The linear correlation coefficient between observed and estimated trends in annual WUE is about 0.93, with a relative bias of 17.2% and mean error of 2.16 mg C mm^{-1} H_2O year^{-1}. The slope of the regressed line between observed and estimated annual WUE passing through the origin is 0.78, with an adjusted R^2 of 0.85.

C3. Agricultural sites are more affected by management practices than by anything else (cropping cycle, tillage, fertilizer use...). None of the global data products you use as input to the global model land-use include agricultural management practices, and I therefore suspect that your predictive ability in the particular agricultural sites you use is strongly driven by incorporating the observations in these sites, and is not representative of global ability to predict GPP in agricultural fields. Another big problem with irrigated fields is that your E estimates are totally wrong for these fields. Your E is based on mass balance. If the irrigation water comes only from very near local sources it would have been fine, but if river, lake or reservoir water is used in irrigation are pumped from far watersheds, or from deep aquifers, the data you include for E will not include these water resources as an input for these location, and therefore, your estimate will be way off.

Responses: We thank the reviewer for this constructive comment. We agree with the reviewer that agricultural management practices affect WUE and GPP. For site specific or local studies, where information on agriculture management practices is available, it is desirable to incorporate these factors. However, the purpose of our study is to estimate regional and global WUE and GPP. At these scales, we believe that the influences of irrigation are not significant enough to undermine the overall trend in WUE and GPP for two reasons. One reason is that the fraction of irrigated land area and the contribution of GPP from irrigated areas to global GPP is very small. Irrigated land area takes only about 2% of terrestrial land area based on FAO's reported irrigated cultivated area (about 0.3 billion hectares). GPP of irrigated areas takes only about 2.5% of global annual GPP (see Table 1 of Beer et al. (2010)) assuming that the ratio of GPP of irrigated areas to that of global total croplands is the same as the ratio of irrigated area to total cropland area. The other reason is that the global diagnostic and reanalysis products of E used in this study can capture some of management effect on water use in agricultural ecosystems through using the remotely sensed canopy LAI, as we explained below. Therefore, the influence of uncertainty in GPP sourced from the irrigated cropland on global GPP and its trend is limited and thus it is very unlikely to alter the conclusion of this study.

To estimate WUE, we assumed that ecosystem WUE can be affected by two vegetation characteristics. One is the physiologic properties of vegetation, i.e. parameter g_1. The other one is the proportion of water used for carbon uptake, i.e. ratio of transpiration to total E (see Section 1 of Supplementary Materials). For agriculture land, our model has explicitly considered crop land use type and vegetation type (i.e. C3 or C4) for parameterization of g_1 (see Section 2.1 in the Supplementary Materials). The influences of agricultural management practices on WUE are considered indirectly through canopy leaf area index and interception ratio datasets, which determines the proportion of water use for carbon uptake and can account for some of the human interferences including cropping cycle, tillage and fertilizer use.

To estimate GPP, agricultural management practices can exert further influences on water use (i.e. E), especially irrigation. In this study, we used seven diagnostic or reanalysis E products, which are the

best global E products currently available at the global scale and represents our most advanced understandings about the global water cycle. The global E products used in this study can capture at least some of the management effects on water use in agricultural ecosystems as they are estimated in terms of both mass balance and energy balance with a wide variety of remotely sensed and ground-based observations of vegetation dynamics, soil moisture, surface temperature, surface radiation fluxes and meteorological forcing information (see Jung et al. (2010); Miralles et al. (2010); Miralles et al. (2011); Miralles et al. (2014); Zeng et al. (2014); Martens et al. (2016); Zhang et al. (2016), *etc.*).

We also agree with the reviewer that irrigation is an important management practice that can significantly affect E and GPP for irrigated agricultural areas. For this reason, sites with known irrigation are excluded for model validation. We checked whether any of the sites are under irrigation. There are 4 sites identified as being affected by irrigation. These 4 sites include two irrigated agricultural sites (US-Ne1 and US-Ne2), one wetland site (AU-Fog) and one rice paddy site (US-Twt), of which 14 station-years (*c.a.* 6% of the total station-years) were used for spatial validation previously. These 14 station-years are excluded for model validation. Therefore, the validation presented should not be influenced by the irrigation issue.

C4. Finally, your main conclusion, highlighted in the abstract, results and discussion section is the bulk global number of GPP increase.

However, given the scarcity of the observation data that could validate this number, particularly with regards to your model ability to detect trends I find this number highly improper and unjustified. To a large degree (as you admit) the global trend you determine is driven by one extreme site observation. Further more, you have no more that 2 points for many ecosystem types.

You should consider a more extensive sensitivity analysis using a Monte Carlo approach of random re-sampling. For example, consider the spread of data among and within points of similar forest types in your figure 1b. If you draw a random sub-set from these points (including the error estimate for each point) you'll find that the slope of the decal trend may vary over a huge range. You should further carry this uncertainty to the global estimate, assuming each point in the global grid can vary within this range. You'll find that the error estimate you provide for your global estimate is highly unrealistic and underestimates the real uncertainty in your process.

It'll be better if your restrict your conclusions to the areas where you do have strong representation of observed data such as mid-latitude forests, and ignore other areas (tropical, boreal, agricultural, semi-arid...)

Responses: Estimated global GPP and WUE are validated not only against the flux observations but also against multiple independent global estimates. We have used as many site-years of flux observations as possible for model validation, which is explained in previous replies and agreed by the reviewer (see C1). Considering the limited flux observations available for model validation, we also collected and used global estimates of GPP and WUE from six state-of-the-art land surface models (LSMs, see Table S5) and an empirical product based on the model tree ensemble (MTE) method (see Results and Discussions in the main text as well as Section 3 and 4 in the Supplementary Materials). Thus, the multiple lines of evidence we assess show a broad agreement with flux observations and independent global estimates of global water and carbon quantities (see also comments of the 1st and 2nd reviewer in the first round supporting the approach).

As for the sensitivity analysis, the reviewer suggested a very constructive approach to quantify the uncertainty in the global GPP trends based on the flux observations. However, the available data are too limited (as also pointed out by the reviewer) to do such extensive analysis. There are only 11 sites for trend validation with six different plant function types (PFTs), of which 1 PFT (evergreen needle leaf forest, (ENF)) has 3 sites, 3 PFTs (evergreen broad leaf forest (EBF), crop (CRO) and woody savannah (WSA)) have two sites and the other two PFTs (grass (GRA) and savannah (SAV)) have only one site. Sites with same PFT have very limited representativeness at the global scale (see Figure R1). For this reason, it would not be robust either to conduct Monte Carlo resampling (or any other resampling technique for sensitive analysis including bootstrapping) or to perform significance tests on a sample with only 3 data points available. We believe that available data are too limited to conduct an extensive sensitivity analysis to quantify the uncertainty in global GPP trend based on the flux data as suggested by the reviewer.

In our analysis, we estimate the uncertainty of our global estimates of WUE and GPP by using multiple independent datasets as inputs. There is an ensemble of 12 and 84 estimates of global WUE and GPP, respectively (see lines 97-98 in the main text). In response to the reviewer's concerns, we further justify our estimated trends in WUE and GPP at global scale against other independent estimates (see line 218-228 in the main text), and we have discussed explicitly the possible higher uncertainties associated with limited observations to make the readers aware of this limitation (see the lines 292-299 in the main text).

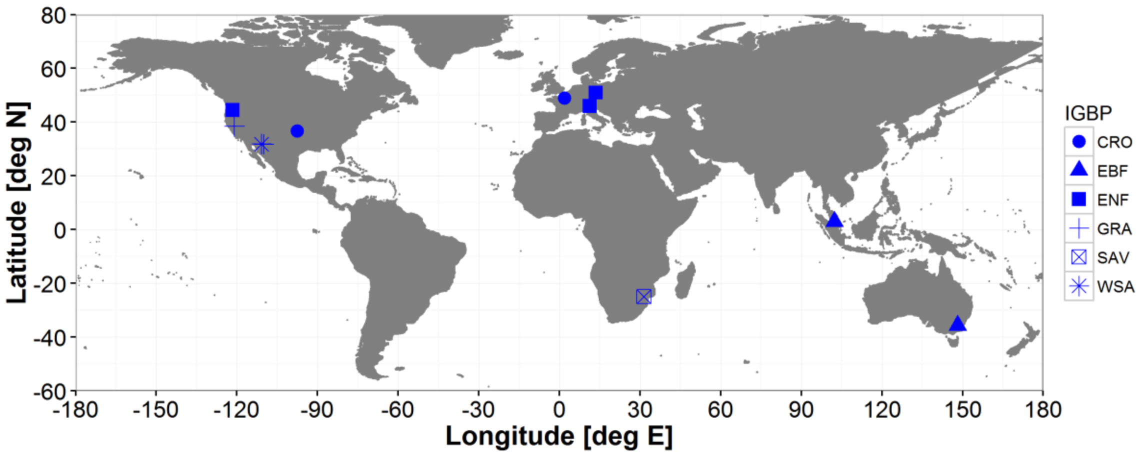

Figure R1 | Distribution of the 11 flux sites used for trend validation. Different shapes of the points represent different plant function types according to the IGBP classification (see Table S4).

C5. Furthermore, there are MANY papers that use remote sensing and global models to estimate the decadal changes in GPP. can you place your estimates within the spread of estimates by these other methods? Are you predicting something extreme and different, or are you within the commonly expected range? That should be done for eco-regions, not only (or not at all) the global number.

Responses: We thank the reviewer for this insightful comment. As suggested, we compared our estimated trends in GPP at global scale and also in different eco-regions with six LSMs in the revised manuscript. Please refers to lines 218-228 in the revised manuscript.

At the global scale, the WEC method estimates a higher mean value and wider range of increase rate in global GPP than that derived from six LSMs over the same period and other reported values. Results from the WEC method with an ensemble of 84 estimates show that global GPP has increased about 0.83±0.26 Pg C year^{-2} over 1982-2011 with a range of 0.33 ~ 1.30 Pg C year^{-2}. Based on the six LSMs from TrendyV3 modelling experiment (Le Quéré et al., 2014), global GPP has increased about 0.44±0.08 Pg C year^{-2} with a range of 0.32 ~ 0.57 Pg C year^{-2} over 1982 – 2011. Other independent studies reported that global GPP has increased from 0.2 to 0.66 Pg C year^{-2} during the past one or two decades. Anav et al. (2013) reported that trends in GPP of the CMIP5 models are from 0.2 to 0.66 Pg C year^{-2} over the period of 1986-2005; Anav et al. (2015) estimated that global GPP has increased at a rate from 0.28 to 0.62 Pg C year^{-2} over the period of 1990-2009 based on seven LSMs; and Jiang and Ryu (2016) reported that global GPP has increased at a rate of 0.27 and 0.35 Pg C year^{-2} over the period of 2001 to 2011 based on the MPI-BGC (Jung et al., 2009) and BESS (Jiang and Ryu, 2016) products, respectively. Basically, the WEC method estimated a larger increase in global GPP in the past three decades than other modelling results, which could be partially resulted from uncertainty in one of the leaf area index products (see the discussion on the uncertainty in LAI products in the discussion section in the main text and Section 6.2 in the Supplementary Materials). Based on the GIMMS LAI3g leaf area index product only, estimated global GPP trend by the WEC method is 0.59±0.12 Pg C year^{-2} (0.33 ~ 0.87 Pg C year^{-2}), which is very close to that derived from six LSMs and to other independent studies.

At 17 different ecoregions, Figure R2 shows that trends in GPP estimated by the WEC method fall within the ranges derived from six LSMs, except the temperate forest in north American (TempF-NAM) and boreal forest (BF) regions, where the WEC method estimated a larger increase rate in GPP than that derived from LSMs.

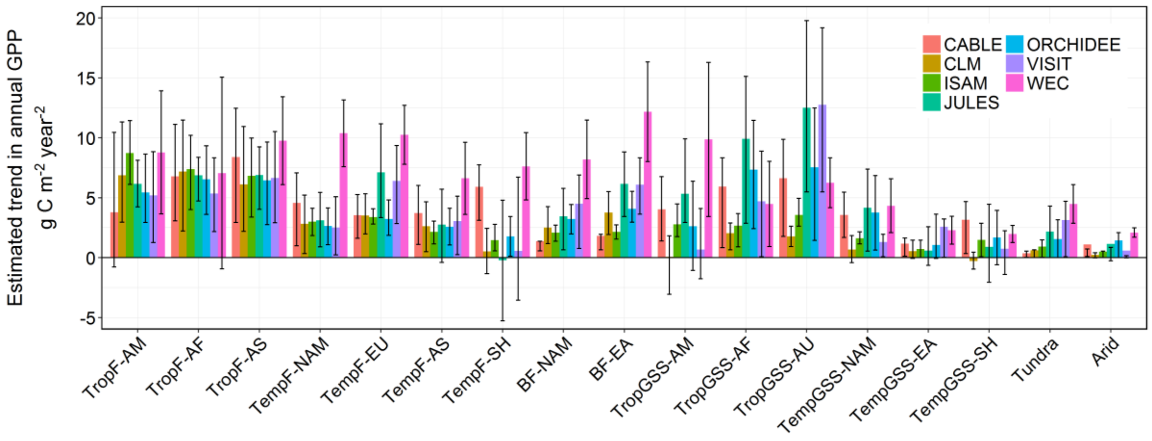

Figure R2 | Comparison of estimated trends reported in this study (i.e. WEC) with 6 LSMs in 17 different eco-regions over the period of 1982-2011. The ecoregions are defined in Table S3 and Figure S4 in the Supplementary Materials. The bars represent of the mean trends of all the grid cells within different ecoregions. The error-bars show the inter-quantile range of different LSMs and one standard deviation of 84 ensembles of the WEC methods, respectively.

Our current understanding about the change in global GPP is still very limited and depends largely on earth system models with significant uncertainty (Beer et al., 2010; Anav et al., 2013; Arora et al., 2013; Bodman et al., 2013; Anav et al., 2015; Medlyn et al., 2015; Jiang and Ryu, 2016). Here, we provide a new way to estimate global GPP and its changes in the past three decades based on the strong

coupling relationship between global carbon and water cycles, and estimated global GPP and its trends are consistent with that derived from very complex earth system models.

In the revised manuscript, we had added a short discussion on our estimated trend in GPP compared with other independent estimates (see lines 218 to 228). An extensive comparison of estimated trends in GPP reported in this study with others from LSMs at global and different eco-regions are provided in the Supplementary Materials (see Section 6.5).

C6. Minor comment - in figure 1b please plot the actual trend model-observed trend line, and its margins of error so that it could be compared with the 1:1 line. showing only the 1:1 line is misleading.

Responses: Thanks. As suggested, regression line between the observed and estimated WUE trend is added in Figure 1b.

C7. Figures 1c-d, 3 and 4 - please state clearly in the captions that these are model predictions. Currently it will mislead readers to assume these are observations.

Responses: We thanks the reviewer for pointing out this oversight. Now stated explicitly that these are estimated products. The caption of Figure 1 c and d has been revised as “estimated spatial details (0.5°×0.5°) of the global mean annual ecosystem WUE”. The caption of the Figure 3 has been revised as “Estimated spatial trends in GPP and WUE over 1982-2011”. The caption of the Figure 4 has been revised as “Estimated spatial variations of the contributions to trends of ecosystem GPP and WUE from different variables over 1982-2011”.

Anav, A., Friedlingstein, P., Beer, C., Ciais, P., Harper, A. and Jones, C. et al., 2015. Spatiotemporal patterns of terrestrial gross primary production: A review. Rev. Geophys., 53(3): 785-818.

Anav, A., Friedlingstein, P., Kidston, M., Bopp, L., Ciais, P. and Cox, P. et al., 2013. Evaluating the land and ocean components of the global carbon cycle in the CMIP5 earth system models. J. Climate, 26(18): 6801-6843.

Arora, V.K., Boer, G.J., Friedlingstein, P., Eby, M., Jones, C.D. and Christian, J.R. et al., 2013. Carbon – concentration and carbon – climate feedbacks in CMIP5 earth system models. J. Climate, 26(15): 5289-5314.

Beer, C., Reichstein, M., Tomelleri, E., Ciais, P., Jung, M. and Carvalhais, N. et al., 2010. Terrestrial gross carbon dioxide uptake: Global distribution and covariation with climate. Science, 329(5993): 834 -838.

Bodman, R.W., Rayner, P.J. and Karoly, D.J., 2013. Uncertainty in temperature projections reduced using carbon cycle and climate observations. Nature Clim. Change, 3(8): 725-729.

Jiang, C. and Ryu, Y., 2016. Multi-scale evaluation of global gross primary productivity and evapotranspiration products derived from Breathing Earth System Simulator (BESS). Remote Sens. Environ., 186: 528-547.

Jung, M., Reichstein, M. and Bondeau, A., 2009. Towards global empirical upscaling of FLUXNET eddy covariance observations: Validation of a model tree ensemble approach using a biosphere model. Biogeosciences, 6(10): 2001-2013.

Jung, M., Reichstein, M., Ciais, P., Seneviratne, S.I., Sheffield, J. and Goulden, M.L. et al., 2010. Recent decline in the global land evapotranspiration trend due to limited moisture supply. Nature, 467(7318): 951-954.

Le Quéré, C., Peters, G.P., Andres, R.J., Andrew, R.M., Boden, T.A. and Ciais, P. et al., 2014. Global carbon budget 2013. Earth Syst. Sci. Data, 6(1): 235-263.

Martens, B., Miralles, D.G., Lievens, H., van der Schalie, R., de Jeu, R.A.M. and Férnandez-Prieto, D. et al., 2016. GLEAM v3: Satellite-based land evaporation and root-zone soil moisture. Geosci. Model Dev. Discuss., 2016: 1-36.

Medlyn, B.E., Zaehle, S., De Kauwe, M.G., Walker, A.P., Dietze, M.C. and Hanson, P.J. et al., 2015. Using ecosystem experiments to improve vegetation models. Nature Clim. Change, 5(6): 528-534.

Miralles, D.G., Gash, J.H., Holmes, T.R.H., de Jeu, R.A.M. and Dolman, A.J., 2010. Global canopy interception from satellite observations. Journal of Geophysical Research: Atmospheres, 115(D16): D16122.

Miralles, D.G., Holmes, T.R.H., De Jeu, R.A.M., Gash, J.H., Meesters, A.G.C.A. and Dolman, A.J., 2011. Global land-surface evaporation estimated from satellite-based observations. Hydrol. Earth Syst. Sc., 15(2): 453.

Miralles, D.G., van den Berg, M.J., Gash, J.H., Parinussa, R.M., de Jeu, R.A.M. and Beck, H.E. et al., 2014. El Niño – La Niña cycle and recent trends in continental evaporation. Nature Clim. Change, 4(2): 122-126.

Zeng, Z., Wang, T., Zhou, F., Ciais, P., Mao, J. and Shi, X. et al., 2014. A worldwide analysis of spatiotemporal changes in water balance-based evapotranspiration from 1982 to 2009. Journal of Geophysical Research: Atmospheres, 119(3): 1186-1202.

Zhang, Y., Peña-Arancibia, J.L., McVicar, T.R., Chiew, F.H.S., Vaze, J. and Liu, C. et al., 2016. Multi-decadal trends in global terrestrial evapotranspiration and its components. Sci. Rep.-UK, 6: 19124.

Reviewers' Comments:

Reviewer #1 (Remarks to the Author)

I am satisfied of how the authors addressed the comments on the article and as I wrote previously this is the first time hydrological variables are used to constrain WUE and GPP and I am confident the obtained results will contribute to the ongoing discussion about trends in WUE and global estimates of GPP.

Line 247-248. Reading the text again, it is likely the authors are trying to avoid writing that LAI cannot affect GPP directly in their model. I would have appreciated a much more explicit statement, at least "only" should be added in line 247, e.g., "The impact of change in L on GPP is only indirectly accounted for in the data…"

Reviewer #3 (Remarks to the Author)

I am happy that the validation does not include site level observation any longer, and that irrigated sites were removed from the analysis. As for model validation, you only show validation for WUE, but your conclusions center on GPP. Please include a figure similar to 1.a that evaluates your model accuracy with respect to GPP. This is critical to establish confidence in your results.

I am still unhappy with the focus on the global number- 0.83±0.26 Pg C year−2.
First - why year^-2? Is that a linear trend of an exponential acceleration rate? Seems unreasonable. Hope it is a typo and should be year^-1 (for a linear trend). The same units are throughout. If it is an exponential acceleration, the consequences of what you claim to the projected GPP in the near future are shocking.
Regardless of the units, I have very little confidence in the accuracy of this number, but at the end it will become the thing everyone cites. As you admit "the available data are too limited ... to do such extensive [sensitivity] analysis...or to perform significance tests on a sample with only 3 data points available" - and if that is the case, how is it not too limiting to generate confidence in the global value you produce? It is unlikely that your parameterization is accurate and representative for any PFT that only have 1-3 sites of observed data. It will be much more constructive to focus on trends that the model indicates, and not focus on one highly inaccurate number.

Within that context, you have improved the discussion of your model predictions by relating it to other LSM predictions, which is good. I am a bit confuse by the writing in line 218-225. You state "the estimated trend in global GPP over 1982-2011 by the WEC method is from 0.33 ~ 1.30 Pg C year−2" which is in direct contradiction with the number which I know from the abstract: 0.83±0.26. I guess that 0.33 ~ 1.30 is the spatial range of the point-wise temporal trends (rather than mean+-1STD), and 0.83 is the global average. But if that is the case, are you comparing your full spatial rage of trends to the global means of the other models? that would be wrong. Taking the global means, your predictions 0.83 are 1.25-4 times higher than anything others have ever predicted (0.22-0.66). That is not a good sign and does not build my confidence in your number.
I also do not accept you interpretation that "Across different eco-regions, trends in GPP estimated by the WEC method agree well with those derived from six LSMs, except for the temperate forest in North American and boreal forest regions" - a quick look in figure R2 (in your response to review, which is also S8) shows large deviations for TempF-SH, TropGSS-AM, which I would hardly classify as "agree well". In fact, you are overestimating by 50-200% of the ensemble mean for all other models in all ecoregion (except TropGSS-AU, TropGSS-AF, TempGSS-EA and TempGSS-NAM).

It will be so much more productive to highlight what you find about the sensitivity of global GPP to water use, and phase down the emphasis on the one global number.

Minor comments:
Fig 1. caption: "Validation of ecosystem WUE..." instead of "validity"

Fig 2 - please add "WEC-modeled trends in ecosystem GPP...". Similar to my previous comments, there is currently no explicit statement that the data shown in this figure is modeled, and can be easily interpreted as observations.

L343 remove "." before "are used"

In several places, references are corrupt and listed as "ref. XX" instead of an subscript number.
For example - Line 56, Line 225

Reviewer #1 (Remarks to the Author):

I am satisfied of how the authors addressed the comments on the article and as I wrote previously this is the first time hydrological variables are used to constrain WUE and GPP and I am confident the obtained results will contribute to the ongoing discussion about trends in WUE and global estimates of GPP.

Line 247-248. Reading the text again, it is likely the authors are trying to avoid writing that LAI cannot affect GPP directly in their model. I would have appreciated a much more explicit statement, at least “only” should be added in line 247, e.g., “The impact of change in L on GPP is only indirectly accounted for in the data…”

Responses: Thanks. Change has been made as suggested. Please refer to line 247 in the revised manuscript.

Reviewer #3 (Remarks to the Author):

C1. I am happy that the validation does not include site level observation any longer, and that irrigated sites were removed from the analysis.

Responses: We are pleased that the reviewer finds the revision on model validation satisfactory.

C2. As for model validation, you only show validation for WUE, but your conclusions center on GPP. Please include a figure similar to 1.a that evaluates your model accuracy with respect to GPP. This is critical to establish confidence in your results.

Responses: At the global scale, estimated global GPP has been compared with other independently estimated mean values and trends from 1982-2011 (see lines 97-101 and 218-230 in the main text and Section 4.2 and 6.5 in the Supplementary Materials and Figure S6). The good agreements of estimated GPP in this study with other independent estimates at the global scale provide further confidence that the new WUE model represents a robust and quantitative measure of the functional coupling between the terrestrial water and carbon cycles, specifically GPP and E.

As suggested, we now added a comparison similar to Figure 1a but for GPP at the site level, please see Figure S5 and Section 3.4 in the Supplementary Materials.

Hope this change adequately addresses the reviewer comment to ensure GPP is appropriately validated against data at multiple levels.

C3. I am still unhappy with the focus on the global number- 0.83±0.26 Pg C year−2.

First - why year^-2? Is that a linear trend of an exponential acceleration rate? Seems unreasonable. Hope it is a typo and should be year^-1 (for a linear trend). The same units are throughout. If it is an exponential acceleration, the consequences of what you claim to the projected GPP in the near future are shocking.

Responses: Global annual GPP is a flux with a unit of Pg C year^{-1}, and thus the trend in annual GPP has to be in Pg C year^{-1}year^{-1} or Pg C year^{-2}. It is just a linear trend over time and not an acceleration rate. The unit of trend in global annual GPP in Pg C year^{-2} was used in several previously published papers (see Anav et al. (2013), Anav et al. (2015), Jiang and Ryu (2016), *et al*.).

C4. Regardless of the units, I have very little confidence in the accuracy of this number, but at the end it will become the thing everyone cites. As you admit "the available data are too limited ... to do such extensive [sensitivity] analysis...or to perform significance tests on a sample with only 3 data points available" - and if that is the case, how is it not too limiting to generate confidence in the global value you produce? It is unlikely that your parameterization is accurate and representative for any PFT that only have 1-3 sites of

observed data. It will be much more constructive to focus on trends that the model indicates, and not focus on one highly inaccurate number.

Responses: We acknowledge and say that "the available data are too limited ... to do such extensive [sensitivity] analysis...or to perform significance tests on a sample with only 3 data points available". This statement was made in response to the review's suggestion to quantify the uncertainty in the estimated trend in GPP of different PFTs using available eddy flux measurements. We would like to perform the analysis suggested, but this would require a much larger number of samples representing different PFTs to be statistically justified and meaningful. However, the current available flux data do not allow us to conduct such extensive sensitivity analysis, yet we have been able to show that the WUE model can well capture the spatiotemporal variability of observed WUE. At the global scale, estimated global WUE and GPP agree well with other published results in both magnitude and spatial patterns. Put it all together, make us conclude that the results of this study are consistent with established knowledge and its conclusions are robust.

We agree that uncertainty still exist in our parameterization scheme, although validity of the proposed model was supported by available observed data at site level and independent estimates at the global scale. We have explicitly stated this potential limitation in the main text (see lines 295-305 in the revised manuscript). We also thanks the reviewer's constructive comments to focus on trends rather than the absolute numbers. Therefore, a short discussion is provide on this issue in the revised manuscript; please refer to lines 230-233 in the revised manuscript.

C5. Within that context, you have improved the discussion of your model predictions by relating it to other LSM predictions, which is good. I am a bit confuse by the writing in line 218-225. You state "the estimated trend in global GPP over 1982-2011 by the WEC method is from 0.33 ~ 1.30 Pg C year−2" which is in direct contradiction with the number which I know from the abstract: 0.83±0.26. I guess that 0.33 ~ 1.30 is the spatial range of the point-wise temporal trends (rather than mean+-1STD), and 0.83 is the global average. But if that is the case, are you comparing your full spatial rage of trends to the global means of the other models? that would be wrong.

Responses: Both the range of 0.33 ~ 1.30 Pg C year^{-2} and mean value of 0.83±0.26 Pg C year^{-2} are the trends at the global scale (not point-wise spatial trends). These values were calculated based on an ensemble of 84 different global estimates (see lines 97-99 in the revised manuscript). So, we are comparing our estimates of global trends with other estimates at the global scale.

C6. Taking the global means, your predictions 0.83 are 1.25-4 times higher than anything others have ever predicted (0.22-0.66). That is not a good sign and does not build my confidence in your number.

Responses: The trend in global GPP over the period of 1982-2011 estimated in this study is indeed larger than those derived from land surface models (LSMs). We think there are two possible explanations, which are not mutually exclusive.

First, several studies published recently pointed out that current LSMs tended to underestimate the response of global carbon cycle and canopy leaf area index (LAI) over growing seasons to environmental changes, especially rising atmospheric CO_2 concentrations (see De Kauwe et al. (2013); Keenan et al. (2013); Zhu et al. (2016)). Thus, it is not outside of the possible that LSMs might underestimate the GPP trend at the global scale, which suggests that the larger trend in global GPP estimated in this study is possibly more reliable than that derived from complex LSMs. More importantly, uncertainty in the proposed WEC method is analytically tractable and results in this study are largely based on the data analysis, whereas results from LSMs usually dependent on the model structures, parameter choices and model calibrations (see Sitch et al. (2008); Wang et al. (2012); De Kauwe et al. (2013); Piao et al. (2013); Anav et al. (2015); *et. al.*) and their uncertainty cannot be quantified effectively as these models are too complex (Arora et al., 2013; Bodman et al., 2013; Friedlingstein et al., 2013; Medlyn et al., 2015).

Second, it is possible that the higher trend in global GPP estimated by the WEC method than that estimated by the LSMs could partially result from uncertainty in one of the LAI products we used. This limitation has been stated and discussed explicitly in the main text and in the Supplementary Materials (see the discussion on the uncertainty in LAI products in the Discussion section in the main text and Section 6.2 and 6.5 in the Supplementary Materials). The estimated trend of global GPP by the WEC method using GIMMS LAI3g product (i.e. the better LAI product) is 0.59±0.12 Pg C year^{-2} (0.33 ~ 0.87 Pg C year^{-2}), or is very close to that derived from six LSMs (0.44±0.08 Pg C year^{-2} with a range of 0.32 ~ 0.57 Pg C year^{-2}) and to other independent studies (0.2 ~ 0.66 Pg C year^{-2}). Estimates of GPP trends by WEC method using the other global LAI products (i.e. GLASS LAI) are still included in this study because (1) the systematic inconsistency in the GLASS LAI occurs only in limited regions, especially in the tropical regions; (2) GLASS LAI is one of important long-term global LAI products that is available for studying the long-term changes in vegetation characteristics globally, as used in many previous studies; and (3) estimated trends in global GPP and WUE from the GLASS LAI products showed consistent spatial patterns with these derived from the GIMMS LAI3g product, except the magnitude being larger.

C7. I also do not accept you interpretation that "Across different eco-regions, trends in GPP estimated by the WEC method agree well with those derived from six LSMs, except for the temperate forest in North American and boreal forest regions" - a quick look in figure R2 (in your response to review, which is also S8) shows large deviations for TempF-SH, TropGSS-AM, which I would hardly classify as "agree well". In fact, you are overestimating by 50-200% of the ensemble mean for all other models in all ecoregion (except TropGSS-AU, TropGSS-AF, TempGSS-EA and TempGSS-NAM).

Responses: Our previous claims on regional agreement were primarily based on the uncertainty range rather than mean trends. There is an ensemble of 84 estimates of global GPP and the mean and 1 standard deviation (mean ± 1sd) of all the 84 estimated are shown in Figure S8 as “WEC” results. Each of the 84 estimates can be considered as an independent estimates of either one of the 6 LSMs. Previously we thought that it is important to take account of uncertainties in comparing two or more estimates because of large uncertainties amongst different estimates from both WEC and LSMs in some regions. It hard to say a trend of 6±4 is significant different from that of 3±6, although the mean values of the two estimates differ by factor of 2. Therefore, trends in GPP of different eco-regions estimated by the WEC

methods can be largely considered in agreement with those from LSMs if the uncertainty of estimates from the WEC method (i.e. error-bar of the WEC in Figure S8) mainly overlaps with the uncertainty of the LSMs (i.e. the maximum range of the error-bar of 6 LSMs in Figure S8). We acknowledge and are in agreement with the reviewer that this does not necessarily mean that the average values agree well with each other, especially when trend of one LSM is significantly different from other models (such as trends of the TempF-SH from 6 LSMs shown in Figure S8).

Therefore, in the revised manuscript, the discussion on agreement has been modified to reflect the reviewer's comment and the discussion above: "Across different eco-regions, trends in GPP estimated by the WEC method agree well with those derived from six LSMs in term of the positive changes. Mean trends from the WEC method are close to those from the LSMs at different ecoregions, except for the temperate and boreal forests and Tundra regions, where the mean trends in GPP estimated using WEC method are much larger than those derived from LSMs".

C8. It will be so much more productive to highlight what you find about the sensitivity of global GPP to water use, and phase down the emphasis on the one global number.

Responses: This study is focusing on assessing the reliability of the WEC method by comparing our estimates with other independent estimates with uncertainties being quantified. The global number is only one of several important conclusions from this study, as stated in the abstract. We believe that by having responded to the above comments, the revised manuscript lends to highlight the most robust results and downplayed those with largest uncertainties, now more clearly stated than before.

Minor comments:

C9. Fig 1. caption: "Validation of ecosystem WUE..." instead of "validity"

Responses: Thanks. Change has been made as suggested.

C10. Fig 2 - please add "WEC-modeled trends in ecosystem GPP...". Similar to my previous comments, there is currently no explicit statement that the data shown in this figure is modeled, and can be easily interpreted as observations.

Responses: Thanks. Change has been made as suggested.

C11. L343 remove "." before "are used"

Responses: Thanks for pointing out this typo. Change has been made as suggested.

C12. In several places, references are corrupt and listed as "ref. XX" instead of an subscript number. For example - Line 56, Line 225

Responses: Format of the references in these two lines (i.e. line 56 and line 255) follows the routine reference style and format of Nature Communications. In these two lines (and other similar), multiple references are listed within brackets to redirect readers to find related information in these references. Under this circumstance, references are typically listed as "(see ref. 1, ref. 2, and ref. 3)" instead of using superscript numbers.

References:

Anav, A., Friedlingstein, P., Beer, C., Ciais, P., Harper, A. and Jones, C. et al., 2015. Spatiotemporal patterns of terrestrial gross primary production: A review. Rev. Geophys., 53(3): 785-818.

Anav, A., Friedlingstein, P., Kidston, M., Bopp, L., Ciais, P. and Cox, P. et al., 2013. Evaluating the land and ocean components of the global carbon cycle in the CMIP5 earth system models. J. Climate, 26(18): 6801-6843.

Arora, V.K., Boer, G.J., Friedlingstein, P., Eby, M., Jones, C.D. and Christian, J.R. et al., 2013. Carbon – concentration and carbon – climate feedbacks in CMIP5 earth system models. J. Climate, 26(15): 5289-5314.

Bodman, R.W., Rayner, P.J. and Karoly, D.J., 2013. Uncertainty in temperature projections reduced using carbon cycle and climate observations. Nature Clim. Change, 3(8): 725-729.

De Kauwe, M.G., Medlyn, B.E., Zaehle, S., Walker, A.P., Dietze, M.C. and Hickler, T. et al., 2013. Forest water use and water use efficiency at elevated CO_2: A model-data intercomparison at two contrasting temperate forest FACE sites. Global Change Biol., 19(6): 1759-1779.

Friedlingstein, P., Meinshausen, M., Arora, V.K., Jones, C.D., Anav, A. and Liddicoat, S.K. et al., 2013. Uncertainties in CMIP5 climate projections due to carbon cycle feedbacks. J. Climate, 27(2): 511-526.

Jiang, C. and Ryu, Y., 2016. Multi-scale evaluation of global gross primary productivity and evapotranspiration products derived from Breathing Earth System Simulator (BESS). Remote Sens. Environ., 186: 528-547.

Keenan, T.F., Hollinger, D.Y., Bohrer, G., Dragoni, D., Munger, J.W. and Schmid, H.P. et al., 2013. Increase in forest water-use efficiency as atmospheric carbon dioxide concentrations rise. Nature, 499(7458): 324 – 327.

Medlyn, B.E., Zaehle, S., De Kauwe, M.G., Walker, A.P., Dietze, M.C. and Hanson, P.J. et al., 2015. Using ecosystem experiments to improve vegetation models. Nature Clim. Change, 5(6): 528-534.

Piao, S., Sitch, S., Ciais, P., Friedlingstein, P., Peylin, P. and Wang, X. et al., 2013. Evaluation of terrestrial carbon cycle models for their response to climate variability and to CO2 trends. Global Change Biol., 19(7): 2117-2132.

Sitch, S., Huntingford, C., Gedney, N., Levy, P.E., Lomas, M. and Piao, S.L. et al., 2008. Evaluation of the terrestrial carbon cycle, future plant geography and climate-carbon cycle feedbacks using five Dynamic Global Vegetation Models (DGVMs). Global Change Biol., 14(9): 2015-2039.

Wang, Y.P., Lu, X.J., Wright, I.J., Dai, Y.J., Rayner, P.J. and Reich, P.B., 2012. Correlations among leaf traits provide a significant constraint on the estimate of global gross primary production. Geophys. Res. Lett., 39(19): L19405.

Zhu, Z., Piao, S., Myneni, R.B., Huang, M., Zeng, Z. and Canadell, J.G. et al., 2016. Greening of the Earth and its drivers. Nature Clim. Change, in press.

Reviewers' Comments:

Reviewer #3:
Remarks to the Author:
You addressed most of my comments.
However, I still do not agree that you have created strong enough confidence to generate a global estimate number. Given the very small number of tropical sites (3) and other ecosystems with only 0-3 sites represented, you simply do not have enough data to evaluate your model globally. I therefore do not agree that your estimate of 0.83+-0.26 makes any sense more than a random number.

You have added few words stating that there is variability and uncertainty, but you ignore it all, do not provide error estimates that incorporate this uncertainty and are still claiming confidence in your global number.

You claim in line 232 " our findings for a significant increase in global GPP are consistent" but admit in the reviewer response letter that the 0.83 estimate, in fact, is a gross overestimation. In the response (but not in the manuscript) you also write that the alternative estimate of 0.56+-12, which is based on a better LAI estimates, is more reasonable overall, and fits better with other global estimates. Please note that 0.56 is not in the range of the 0.83+-26 estimate. Why are you insisting on the high estimate then? And obviously, your error estimate is wrong (underestimated), as it does not include the realistic estimate.

I do not think we will agree on this. It may be a difference of opinion that the editor will have to decide about, but I do not believe that your global estimate is justified, or reasonable.

Reviewer #3 (Remarks to the Author):

C1. You addressed most of my comments.

Responses: We appreciate the efforts by the reviewer in assessing our manuscript, and we are pleased to know that the revised manuscript addressed most concerns.

C2. However, I still do not agree that you have created strong enough confidence to generate a global estimate number. Given the very small number of tropical sites (3) and other ecosystems with only 0-3 sites represented, you simply do not have enough data to evaluate your model globally. I therefore do not agree that your estimate of 0.83+-0.26 makes any sense more than a random number.

Responses: Currently, FLUXNET 2015 dataset is the most updated global flux dataset available to the public. In our study, we used ALL the flux data in FLUXNET 2015 dataset that meet our selection criteria. We acknowledge that the available observations is still limited for some ecosystem types at present, particularly for tropical and boreal forests. Our estimates will be improved in the future as more observations are taken and made available. I hope that the reviewer would agree with us that this is how we make progress in science.

In the revised manuscript, two changes have been made to further clarify the issue about the limited flux data for model validation and the related assessment of uncertainty. One is that we are now focusing more on the range of GPP trend estimates rather than on the mean value (see lines 109-120). The other one is we added more discussion on the limitations of available data for model validation (see lines 307-311).

C3. You have added few words stating that there is variability and uncertainty, but you ignore it all, do not provide error estimates that incorporate this uncertainty and are still claiming confidence in your global number.

Responses: We didn't choose to ignore the issue in our previous revision but what is proposed by the review cannot be performed with the available data. The uncertainty analysis suggested by the reviewer is not statistically meaningful given the relative small number of stations available per plant function type in the global flux dataset with our data selection criteria. In quantifying the uncertainty of GPP trend presented, we account for some additional uncertainties in meteorological forcing and model inputs including E, canopy interception and canopy LAI.

C4. You claim in line 232 " our findings for a significant increase in global GPP are consistent" but admit in the reviewer response letter that the 0.83 estimate, in fact, is a gross overestimation. In the response (but not in the manuscript) you also write that the alternative estimate of 0.56+-12, which is based on a better LAI estimates, is more reasonable overall, and fits better with other global estimates. Please note that 0.56 is not in the range of the 0.83+-26 estimate. Why are you insisting on the high estimate then? And obviously, your error estimate is wrong (underestimated), as it does not include the realistic estimate.

I do not think we will agree on this. It may be a difference of opinion that the editor will have to decide about, but I do not believe that your global estimate is justified, or reasonable.

Responses: We respect the reviewer's view and appreciate the critical comment here.

The estimated trends in global GPP of 0.83±0.26 Pg C $year^{-2}$ and 0.59±0.12 Pg C $year^{-2}$ come, respectively, from the mean value of an ensemble of 84 estimates of GPP (using two LAI products: GIMMS-LAI3g and GLASS) and of an ensemble of 42 estimates of GPP using the GIMMS-LAI3g LAI only.

The estimated trend of 0.59±0.12 Pg C $year^{-2}$ is considered a better estimate because spurious systematic inconsistency in the GLASS LAI product was identified (see section 6.2 of Supplementary Materials). This lower estimate also happens to be closer to other published estimates.

Thus, in response to the reviewer comment, we call this lower estimate (0.59±0.12 Pg C $year^{-2}$) our "best estimate" in the revised manuscript, based on the knowledge of the full ensemble of estimates and also on using GLASS LAI is less robust. Please refer to lines 109-114 in the revised manuscript.

To let readers have a clear understanding about the uncertainty in estimated global GPP trend in the revised manuscript, we clearly show both the full ensemble and the best estimate (see lines 109-114), and move the comparison of trends from our method with other independent estimates, from the Discussion to the Results (see lines 115-120); this allows the reader to have a more complete view of the uncertainties in one single place.
